# Data Assessment on the relationship between typical weather data and electricity consumption of academic building in Melaka

**DOI:** 10.1016/j.dib.2021.106797

**Published:** 2021-02-01

**Authors:** Afiqah Ngah Nasaruddin, Boon Tuan Tee, Musthafah Mohd Tahir, Md Eirfan Safwan Md Jasman

**Affiliations:** a Fakulti Kejuruteraan Mekanikal, Universiti Teknikal Malaysia Melaka, Hang Tuah Jaya, Durian Tunggal, Melaka 76100, Malaysia; b Centre for Advanced Research on Energy, Universiti Teknikal Malaysia Melaka, Hang Tuah Jaya, Durian Tunggal, Melaka 76100, Malaysia

**Keywords:** JASP software, Weather condition, Causal correlation, Typical weather data

## Abstract

Exposure to hot and humid weather conditions will often lead to consuming a vast amount of electricity for cooling. Heating, ventilation, and air conditioning (HVAC) systems are customarily known as the largest consumers of energy in institutions and other facilities which raises the question regarding the impact of the weather conditions to the amount energy consumed. The academic building is a perfect example where a constant fixed daily operating characteristic is measured by the hour, aside from the occasional semester break. Therefore, it can be assumed that the daily HVAC services on an academic facility will operate on a fixed schedule each day, having a similar pattern all year round. This article aims to present an analysis on the relationship between typical weather data by implying the test reference year (TRY) and academic building electricity consumption in an academic building located at Durian Tunggal, Melaka. Typical weather data were generated in representing the weather data between 2010 and 2018 using the Finkelstein–Schafer statistic (F-S statistic) in addition to a data set of electricity consumption. Descriptive analysis and correlation matrix analysis were conducted using JASP software for two sets of sample data; Set A and Set B, with data points of 12 and 108, respectively. The result showed an alternate result with a positive correlation between 1)mean temperature-electricity consumption, and 2)mean rainfall-electricity consumption for data Set A, and a negative correlation between 1)mean temperature-electricity consumption and 2)mean rainfall-electricity consumption for data Set B.

## Nomenclature

AWS: Automatic Weather System
BF: Bayes Factor
CDF: Cumulative Distribution Function
F-S: Finkelstein-Schafer
HVAC: Heating, Ventilation, and Air Conditioning
MST: Mountain Standard Time
TNB: Tenaga Nasional Berhad
TRY: Test Reference Year
UTeM: Universiti Teknikal Malaysia Melaka
WMO: World Meteorology Organization

## Specifications Table

**Table:** 

Subject area	Engineering
Specific subject area	Mechanical engineering: Causal Correlation between two variables, namely, electricity consumption and weather condition variables. The weather condition variables are narrowed down to three parameters: mean temperature, mean relative humidity and mean rainfall.
Type of data	Tables: a. Selection of the Test Reference Year (TRY) based on the FS value (mean temperature, mean relative humidity, mean rainfall). b. Descriptive statistic for correlation analysis for Set A and Set B. c. Overall correlation analysis for Set A and Set B. d. Scatter plot for the correlation analysis for Set A and Set B. Figures: a. Graph of the long-term cumulative distribution function (CDF) and short-term CDF for the three-weather parameters (mean temperature, mean relative humidity and mean rainfall) and electricity consumption between 2010-2018. b. Graph of the long-term CDF, best CDF, and worst CDF for the three-weather parameters (mean temperature, mean relative humidity and mean rainfall) and electricity consumption between 2010-2018. c. Flow of an automatic weather system (AWS) consisting of the acquisition electronic and AWS interface. d. Autonomous tipping bucket inside - top view and side view. e. Autonomous thermometer screen inside view and side view.
How data were acquired	a. Weather data were retrieved from the Meteorology Department of Malaysia based on daily weather data captured by an automatic weather station located at Batu Berendam, Melaka. The weather station was equipped with an autonomous thermometer screen and an autonomous tipping bucket that collected information on the temperature, relative humidity, and rainfall. b. Electricity consumption for the main university campus was estimated using the utility bill provided by the utility company- Tenaga Nasional Berhad (TNB). c. JASP software for statistical analysis was employed for further data treatment in addition to using Microsoft Excel.
Data format	Raw Data: a. Weather data (daily mean temperature, mean relative humidity and rainfall). b. Monthly Electricity consumption. Analysis: a. TRY weather data were generated using the Finkelstein–Schafer statistic method. b. The CDF and correlation matrix between TRY weather data and electricity consumption were determined. Filtered: The outlier detected on the rainfall reading (-33.3) represented a trace reading but less than 0.2 mm.
Parameters for data collection	The weather data and electricity consumption (daily and monthly) for analysis represented a nine-year period (2010-2018).
Description of data collection	a. For the weather data, the parameters analysed included the daily mean temperature, mean relative humidity and rainfall. The temperature and rainfall readings were safely logged into the AWS system. b. Electricity consumption was measured each month via utility companies before issuing to the management of the university.
Data source location	Institution: Pejabat Meteorologi Melaka (Melaka main weather station) City/Town/Region: Batu Berendam, Melaka, Malaysia. Coordinate: (102.24E, 2.25N) Samples/data: Weather data Institution: Universiti Teknikal Malaysia (UTeM) Main Campus City/Town/Region: Durian Tunggal, Melaka, Malaysia. Coordinate: (102.19E, 2.18N) Samples/data: Electricity consumption
Data accessibility	Data within this article and shared within the supplementary material.

**Value of the Data**

• The data would provide a real weather variable pattern representing the Melaka city area specifically for the location close to Batu Berendam, Melaka.
• The data would provide a threshold for TRY weather data correlation and energy consumption for academic facilities.
• The data would provide the researcher with an opportunity to replicate the statistical analysis of this study, as a reliable source, in progressing research to investigate the impact of weather variables on the energy performance of a facility.

## 1 Data Description

The weather variables of this research included the mean temperature (°C), mean relative humidity (%) and mean rainfall (mm) at 08-08 Mountain Standard Time (MST) based onthe data provided by the Meteorology Department of Malaysia for the Batu Berendam, Melaka weather station. The daily raw data between 1^st^ January 2010 and 31^st^ December 2018 were retrieved from the weather station, equipped with an autonomous measuring instrument having recorded hourly readings through the Automatic Weather System (AWS). Based on the raw data the TRY representing weather data over the nine years was generated, in describing typical weather data of a specific location using the Finkelstein–Schafer statistic method (F-S statistic), referred to as the supplementary file for the Melaka 2010-2018 test reference year.

In contrast, the estimation of monthly electricity consumption data on selected academic facilities was based on the utility bill issued each month by the national utility company, Tenaga Nasional Berhad (TNB) referred to as the supplementary file TRY weather data vs electricity. The cumulative distribution function (CDF) was later calculated for both the weather data set and electricity consumption data set to compute their respective FS statistic. The supplementary file under the name graph best and worst weather data and graph best and worst electricity consumption displayed the graph for monthly best and worst weather data parameters and electricity consumption, respectively.

**Fig. 1:**
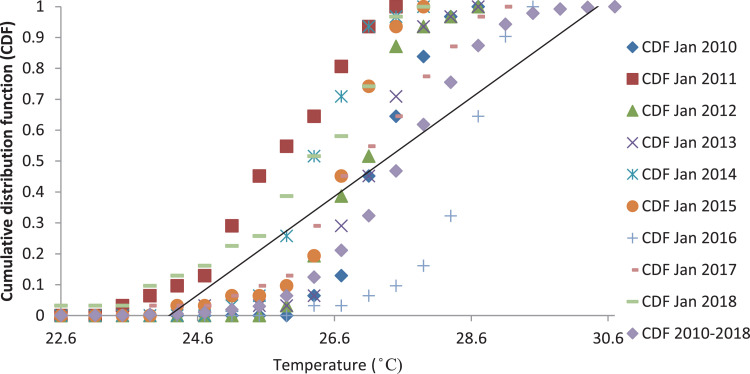
Graph of the long-term CDF and short-term CDF (January) for the mean temperature.

**Fig. 2:**
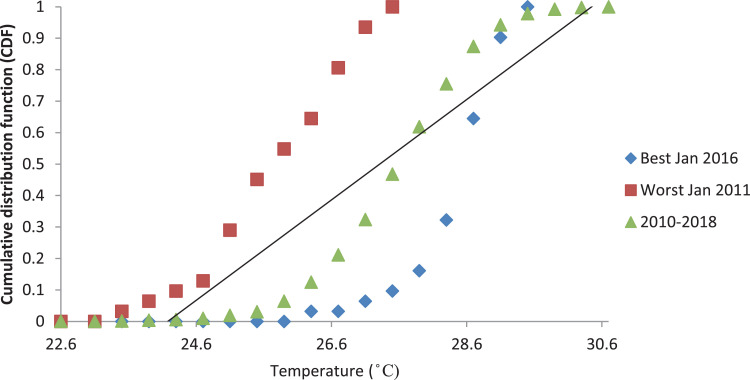
Graph of the long-term CDF, best CDF (January 2016) and worst CDF (January 2011) for the mean temperature.

**Fig. 3:**
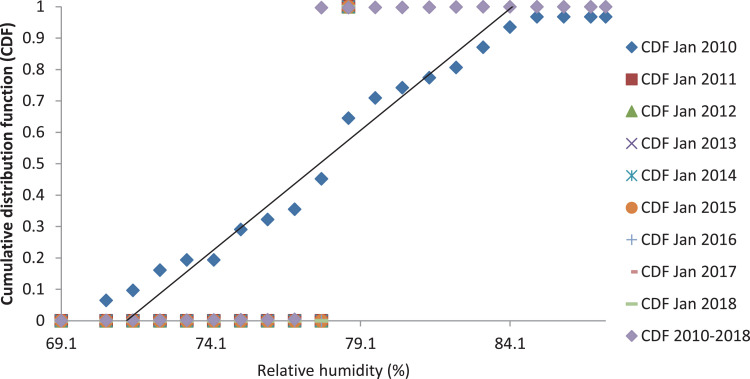
Graph of the long-term CDF and short-term CDF (January) for the mean relative humidity.

**Fig. 4:**
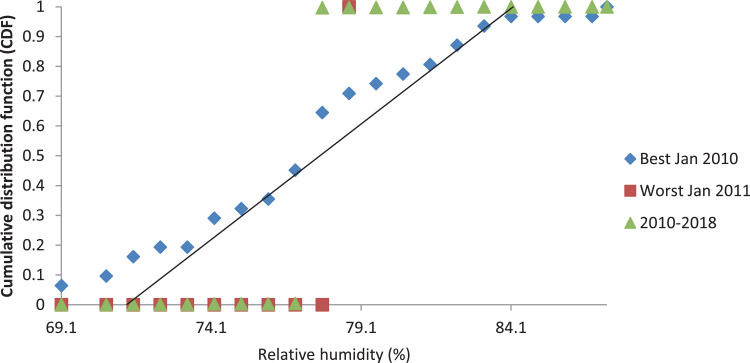
Graph of the long term CDF, best CDF (January 2010) and worst CDF (January 2011) for the mean relative humidity.

**Fig. 5:**
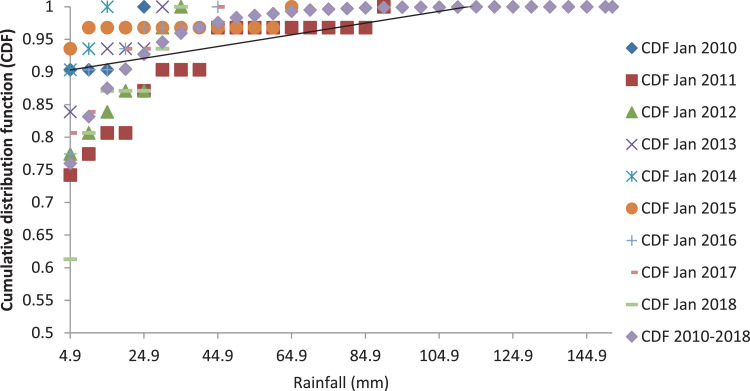
Graph of the long-term CDF and short-term CDF (January) for the mean rainfall.

**Fig. 6:**
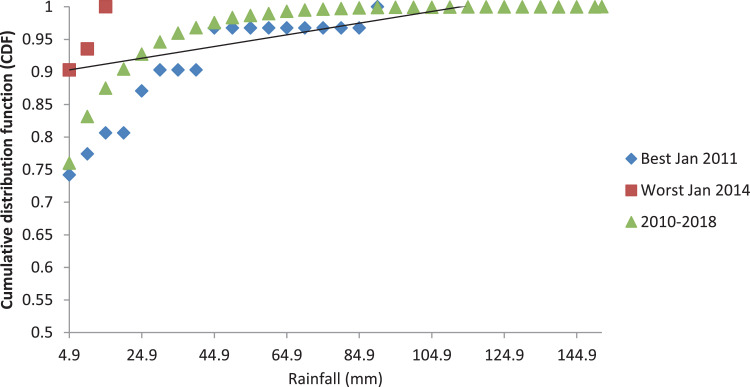
Graph of the long-term CDF, best CDF (January 2011) and worst CDF (January 2014) for the mean rainfall.

Figs. 1, 3 and 5 show the graph of the long-term CDF and short-term CDF of the sample month of January for the mean temperature, mean relative humidity and mean rainfall, respectively. While Figs. 2, 4 and 6 show the graph of the long-term CDF and their respective best and worst CDF for the mean temperature, mean relative humidity and mean rainfall, respectively. For instance, Fig. 1 signifies the solid straight-line posterior distribution of long-term CDF for the temperature variables which were then used for the decision of best (January 2016) and worst (January 2011) temperature variable CDF as represented in Fig. 2. A similar explanation is given for the relative humidity variable, as shown in Figs. 3, 4 having the best and worst CDF for January 2010 and January 2011, respectively. While Figs. 5, 6 signifying the rainfall variable indicate the best and worst of CDF in January 2011 and January 2014, respectively.

In other words, the ‘best CDF’ means that the data distribution pattern is the best representative of all candidates for that specific month (short-term) since it is almost imitating the distribution pattern of the aforementioned long-term CDF.

Contrastingly, Table 1, Table 2, Table 3 portray the FS value for the mean temperature (Table 1), mean relative humidity (Table 2) and mean rainfall (Table 3) for nine years. It can be seen that the number underlined and in bold represent the smallest FS for each month, and are selected to be a part of TRY. For the mean temperature parameter in January 2016, February 2016, March 2014, April 2012, May 2013, June 2017, July 2015, August 2015, September 2013, October 2010, November 2010, and December 2016, these represent part of the TRY. Regarding the mean relative humidity, the decision is much simpler, which is the combination of January 2010 and February to December 2018 to be appointed as TRY. Whereas for the mean rainfall parameter, January 2011, February 2010, March 2017, April 2018, May 2016, June 2016, July 2017, August 2017, September 2017, October 2011, November 2017, and December 2017 are in favour.

**Table 1:** Selection of Test Reference Year (TRY) based on FS value (mean temperature).

Month	2010	2011	2012	2013	2014	2015	2016	2017	2018
January	0.2883	0.5190	0.3100	0.2932	0.4374	0.3944	**0.2480**	0.2598	0.4676
February	0.2470	0.2701	0.3178	0.3594	0.2361	0.2783	**0.2324**	0.2997	0.2803
March	0.1791	0.2269	0.2686	0.2449	**0.1591**	0.2016	0.2553	0.1755	0.1763
April	0.1732	0.2276	**0.1121**	0.1536	0.2605	0.2079	0.2360	0.1952	0.1653
May	0.2175	0.1545	0.1391	**0.1355**	0.2350	0.2144	0.1954	0.2157	0.1920
June	0.1952	0.2291	0.1764	0.1838	0.1709	0.2365	0.2190	**0.1272**	0.1664
July	0.2700	0.2821	0.2826	0.2644	0.2166	**0.1324**	0.1453	0.2452	0.2185
August	0.2806	0.2623	0.2777	0.2644	0.2727	**0.1608**	0.1647	0.2567	0.2329
September	0.2717	0.2689	0.2743	**0.1763**	0.3099	0.2339	0.2240	0.2726	0.1969
October	**0.1388**	0.2947	0.2522	0.2293	0.2234	0.2296	0.1760	0.1755	0.2173
November	**0.1545**	0.3583	0.3230	0.3282	0.2615	0.2304	0.2398	0.3393	0.2751
December	0.4107	0.4107	0.3360	0.3345	0.3732	0.1684	**0.1256**	0.2388	0.2933

**Table 2:** Selection of Test Reference Year (TRY) based on FS value (mean relative humidity).

Month	2010	2011	2012	2013	2014	2015	2016	2017	2018
January	**0.1780**	0.5243	0.5243	0.5243	0.5243	0.5243	0.5243	0.5243	0.5243
February	0.5243	0.5243	0.5243	0.5243	0.5243	0.5243	0.5243	0.5243	**0.5243**
March	0.5243	0.5243	0.5243	0.5243	0.5243	0.5243	0.5243	0.5243	**0.5243**
April	0.5243	0.5243	0.5243	0.5243	0.5243	0.5243	0.5243	0.5243	**0.5243**
May	0.5243	0.5243	0.5243	0.5243	0.5243	0.5243	0.5243	0.5243	**0.5243**
June	0.5243	0.5243	0.5243	0.5243	0.5243	0.5243	0.5243	0.5243	**0.5243**
July	0.5243	0.5243	0.5243	0.5243	0.5243	0.5243	0.5243	0.5243	**0.5243**
August	0.5243	0.5243	0.5243	0.5243	0.5243	0.5243	0.5243	0.5243	**0.5243**
September	0.5243	0.5243	0.5243	0.5243	0.5243	0.5243	0.5243	0.5243	**0.5243**
October	0.5243	0.5243	0.5243	0.5243	0.5243	0.5243	0.5243	0.5243	**0.5243**
November	0.5243	0.5243	0.5243	0.5243	0.5243	0.5243	0.5243	0.5243	**0.5243**
December	0.5243	0.5243	0.5243	0.5243	0.5243	0.5243	0.5243	0.5243	**0.5243**

**Table 3:** Selection of Test Reference Year (TRY) based on FS value (mean rainfall).

Month	2010	2011	2012	2013	2014	2015	2016	2017	2018
January	0.8439	**0.4407**	0.7775	0.8120	0.9027	0.5996	0.7152	0.7120	0.7804
February	**0.4423**	0.8497	0.5867	0.8416	0.9535	0.7530	0.5584	0.7855	0.8197
March	0.8103	0.7834	0.8388	0.8787	0.8777	0.7455	0.9535	**0.5592**	0.7844
April	0.6933	0.5275	0.6846	0.7454	0.8137	0.5876	0.7474	0.7779	**0.4957**
May	0.8673	0.6245	0.6522	0.8725	0.6543	0.6809	0.5575	0.7778	0.6097
June	0.6224	0.7764	0.8765	0.7265	0.4659	0.5344	0.2976	0.5966	0.5760
July	0.7490	0.8449	0.7739	0.6852	0.7050	0.6541	0.6861	**0.4126**	0.8735
August	0.6889	0.7460	0.8407	0.8099	0.6286	0.3397	0.7500	**0.0567**	0.7481
September	0.7808	0.7164	0.4968	0.6191	0.7450	0.6840	0.8387	**0.4966**	0.7765
October	0.7804	**0.4616**	0.5951	0.6823	0.6600	0.5987	0.6197	0.6197	0.7480
November	0.6679	0.7185	0.7317	0.6344	0.7069	0.7513	0.5404	**0.5318**	0.7246
December	0.6824	0.8075	0.8704	0.7782	0.7823	**0.4720**	0.6297	0.5309	0.5915

**Fig. 7:**
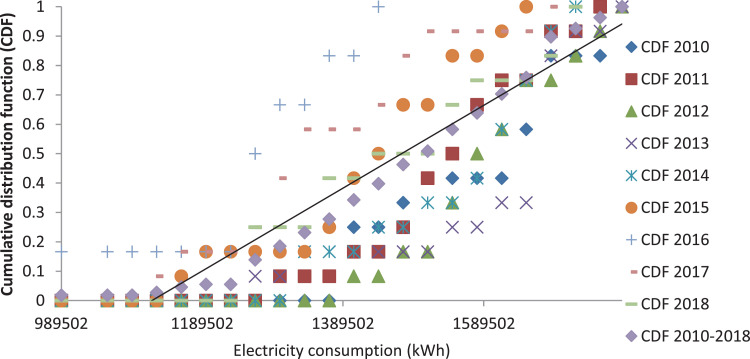
Graph of the long-term CDF and short term CDF for the electricity consumption.

**Fig. 8:**
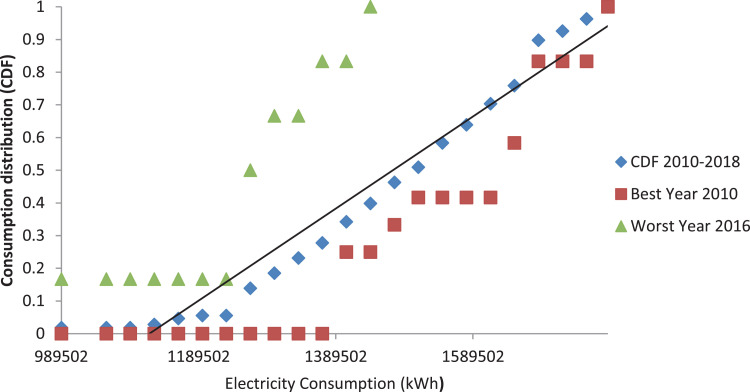
Graph of the long-term CDF, best CDF (Year 2010) and worst CDF (Year 2016) for the electricity consumption.

The monthly electricity consumption data were treated in the same manner as that of CDF presented in Figs. 7, 8. In Fig. 7, the graph shows the compilation of the long-term CDF and the short-term CDF of individual years. However, Fig. 8 demonstrates the best and the worst of the electricity consumption parameter. The best and the worst year of the data set were determined in accordance with the FS statistic used in generating the typical weather data. Nevertheless, the best year is 2010, and the worst year is 2016 accordingly in comparison to the long-term year as a threshold.

On the other hand, the open-source statistical analysis software platform, namely JASP software, was used to perform the descriptive statistical analysis and correlation matrix analysis. Tables 4, 5 show the descriptive statistic presented into two sets of data, Set A and Set B. Set A of the data sample consists of 12 data points while Set B consists of 108 data points. Here, the number of data points that differ in both sets of the sampled data is where Set A represents typical weather data for the respective month of the year while Set B represents typical weather data for the respective day of a month for over the nine years.

**Table 4:** Descriptive statistic for correlation analysis Set A.

Descriptive statistic	Mean temperature	Mean relative humidity	Mean rainfall	Electricity consumption
Sample, n	12	12	12	12
Mean	27.86	77.27	8.15	1,573,000
Standard deviation	0.4	0.1	2.7	141,652.5
Skewness	−0.518	−3.464	0.987	−0.101
Kurtosis	−0.98	12.00	1.731	−1.519
maximum	28.42	77.3	14.48	1,768,000
Minimum	27.11	76.94	3.85	1,383,000

**Table 5:** Descriptive statistic for correlation analysis Set B.

Descriptive statistic	Mean temperature	Mean relative humidity	Mean rainfall	Electricity consumption
Sample, n	108	108	108	108
Mean	27.861	77.27	8.15	1,483,000
Standard deviation	0.419	0.1	2.6	179,183.1
Skewness	−0.457	−3.058	0.871	−0.423
Kurtosis	−1.070	7.488	1.715	−0.442
maximum	28.419	77.3	14.48	1,786,000
Minimum	27.113	76.94	3.85	989,502

However, it can be seen that there is a reasonable similarity between the descriptive analysis for the correlation analysis in Set A and Set B. This is because the sample of electricity consumption utilised was the best representative that was selected from the nine years using the method described in the previous section. It is the best distribution that mimics the whole pattern distribution of electricity consumption. A similar explanation is given for the typical weather data or the TRY used for the correlation analysis for both sets. Concerning data skewness, it can be inferred that the tendency of the mean temperature, mean relative humidity and electricity consumption is prone to the left tail. In other words, the distribution is negatively skewed. However, the mean rainfall parameter distribution is positively skewed, having a bulk amount of data on the right tail.

Furthermore, the result from the correlation analysis, Set A and Set B, is presented in Tables 6, 7, respectively. For Set A (Table 6), the negative correlation can be observed between the 1)mean temperature-the mean relative humidity and 2)mean relative humidity-mean rainfall for all three-correlation tests carried out. On the other hand, a positive correlation is observed between the 1)mean temperature-mean rainfall, 2)mean temperature-electricity consumption and 3)mean rainfall-electricity consumption for all three-correlation measures.

**Table 6:** Overall correlation analysis for Set A.

Parameter	Correlation test	Mean temperature	Mean relative humidity	Mean rainfall
Mean temperature	Pearson's r	−	−	−
	Spearman's rho	−	−	−
	Kendall's tau	−	−	−
Mean relative humidity	Pearson's r	−0.404	−	−
	Spearman's rho	−0.481	−	−
	Kendall's tau	−0.411	−	−
Mean rainfall	Pearson's r	0.169	−0.067	−
	Spearman's rho	0.137	−0.131	−
	Kendall's tau	0.076	−0.111	−
Electricity consumption	Pearson's r	0.287	−0.086	0.053
	Spearman's rho	0.312	0.044	0.007
	Kendall's tau	0.198	0.037	0.000

**Table 7:** Overall correlation analysis for Set B.

Parameter	Correlation test	Mean temperature	Mean relative humidity	Mean rainfall
Mean temperature	Pearson's r	−	−	−
	Spearman's rho	−	−	−
	Kendall's tau	−	−	−
Mean relative humidity	Pearson's r	−0.404	−	−
	Spearman's rho	−0.481	−	−
	Kendall's tau	−0.411	−	−
Mean rainfall	Pearson's r	0.169	−0.067	−
	Spearman's rho	0.137	−0.131	−
	Kendall's tau	0.076	−0.111	−
Electricity consumption	Pearson's r	−0.131	−0.043	−0.158
	Spearman's rho	−0.096	0.044	−0.153
	Kendall's tau	−0.064	0.036	−0.110

As for the correlation between the mean relative humidity and electricity consumption, the negative correlation resulted from Pearson's r test and the positive correlation quotient from Spearman's rho and Kendall's tau. Likewise, the correlation analysis for Set B (Table 7) shows almost a similar outcome as for Set A, except that in this case, a negative correlation was observed for all three-correlation measures for the relationship between 1)mean temperature-electricity consumption and 2)mean rainfall-electricity consumption.

Finally, the Bayesian pair correlation for Bayes Factors, BF_10_ confidence interval under the alternative hypothesis was carried out for both data sets in which focused on Pearson's r correlation analysis and the scatter plot, which was projected based on that established idea. The alternative hypothesis suggests that there is a positive association between the two variables tested. Tables 8, 9 show a scatter plot for Set A and Set B Pearson's r correlation analysis. Moreover, it visualises the distribution that constitutes the entire Pearson's r correlation coefficient with the assumption that it must be a continuous type of data with a linear relationship between the two assigned variables and no outliers whatsoever. Therefore, the causal correlation between the parameters, for instance, a)temperature and relative humidity, b)temperature and rainfall, c)temperature and electricity consumption, d)relative humidity, e)relative humidity and electricity consumption and f)rainfall and electricity consumption was projected for both Set A and Set B.

**Table 8:** Scatter plot for the correlation analysis Set A.

**Table 9:** Scatter plot for the correlation analysis Set B.

The concept of the posterior-prior plot for this Bayesian pair correlation is that if the unconnected dot before distribution is higher than the straight-line posterior distribution, thus the BF_10_ is in favour of the alternative hypothesis (positive correlation) and vice versa. Referring to the unconnected dot and the straight-line of the posterior-prior plot in Table 8 it shows that (a) temperature is negatively correlated to relative humidity, (b) temperature is positively correlated to rainfall, (c) temperature is positively correlated to electricity consumption, (d) relative humidity is negatively correlated to rainfall, (e) relative humidity is negatively correlated to electricity consumption and (f) rainfall is positively correlated to electricity consumption. In contrast, the posterior-prior plot in Table 9, shows that (a) temperature is negatively correlated to relative humidity, (b) temperature is positively correlated to rainfall, (c) temperature is negatively correlated to electricity consumption, (d) relative humidity is negatively correlated to rainfall, (e) relative humidity is positively correlated to electricity consumption and (f) rainfall is negatively correlated to electricity consumption.

Based on both correlation analysis above (Pearson's r, Spearman's rho, Kendall's tau, and Bayesian pairs correlation) it can be safely deduced that for data Set A, a promising medium positive correlation (0.312-Spearman's rho and 0.287-Pearson's r) was detected between the mean temperature and electricity consumption, whereas for data Set B, a weak positive correlation (0.044-Spearman's rho and 0.036-Kendall's tau) was confirmed between mean relative humidity and electricity consumption.

## 2 Experimental Design, Materials and Methods

The weather data to be analysed in this experiment consisted of various parameters, namely 24 hours mean temperature (°C), 24 hours mean relative humidity (%) and 08-08 MST rainfall (mm). All three parameters were retrieved from one of the main automatic weather stations at Batu Berendam, Melaka (2°16’N, 102°15’E) by the Meteorology Department of Malaysia. The Batu Berendam's weather station is the nearest main station which is approximately 10.90 km from the main university campus. The reliability of the weather data was based on the assumption that even if the weather condition were to change every 5 km, it would not make a significant difference regarding the climate condition since it involves only a two-radius difference. Thus, the weather information from this station represented the weather condition at the main university campus.

Moreover, the method of weather data acquisition comprised of both hourly logged AWS and 08-08 MST (mm) via a manually acquired weather measuring instrument which concurrently functions as a support for data validation in case one of the instrument categories failed. The main station was completed having an autonomous measuring instrument to extract multiple meteorology parameters such as the Tipping bucket (rainfall), PT 100 sensor (wet bulb and dry bulb temperature), Anemometer (wind direction and speed) Solarimeter (solar irradiance) and high-volume air sample (air pollutant index). In addition, it was equipped with manual meteorology devices such as the Rain gauge (rainfall), Thermometer screen (maximum and minimum temperature, dry and wet bulb temperature) and Evaporation pond (rate of natural evaporation). In the end, all logged readings from the autonomous instrument were channelled to acquisition electronics which were monitored on the AWS interface. All the meteorology measuring instruments were assigned and set up based on the guidelines provided by the World Meteorology Organisation (WMO). For instance, the meteorology instrument placed at the standardised height and distance between one measuring instruments to the other was also uniquely considered to prevent any meteorology data interference.

**Fig. 9:**
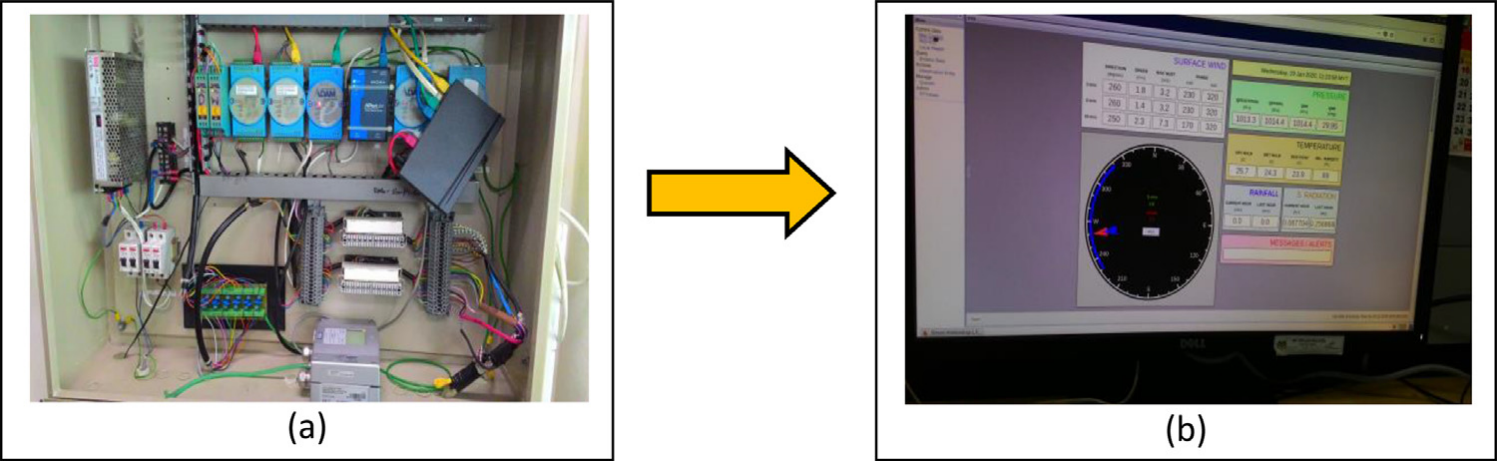
Flow of Automatic weather system (AWS) consist of acquisition electronics (a) and AWS interface (b).

**Fig. 10:**
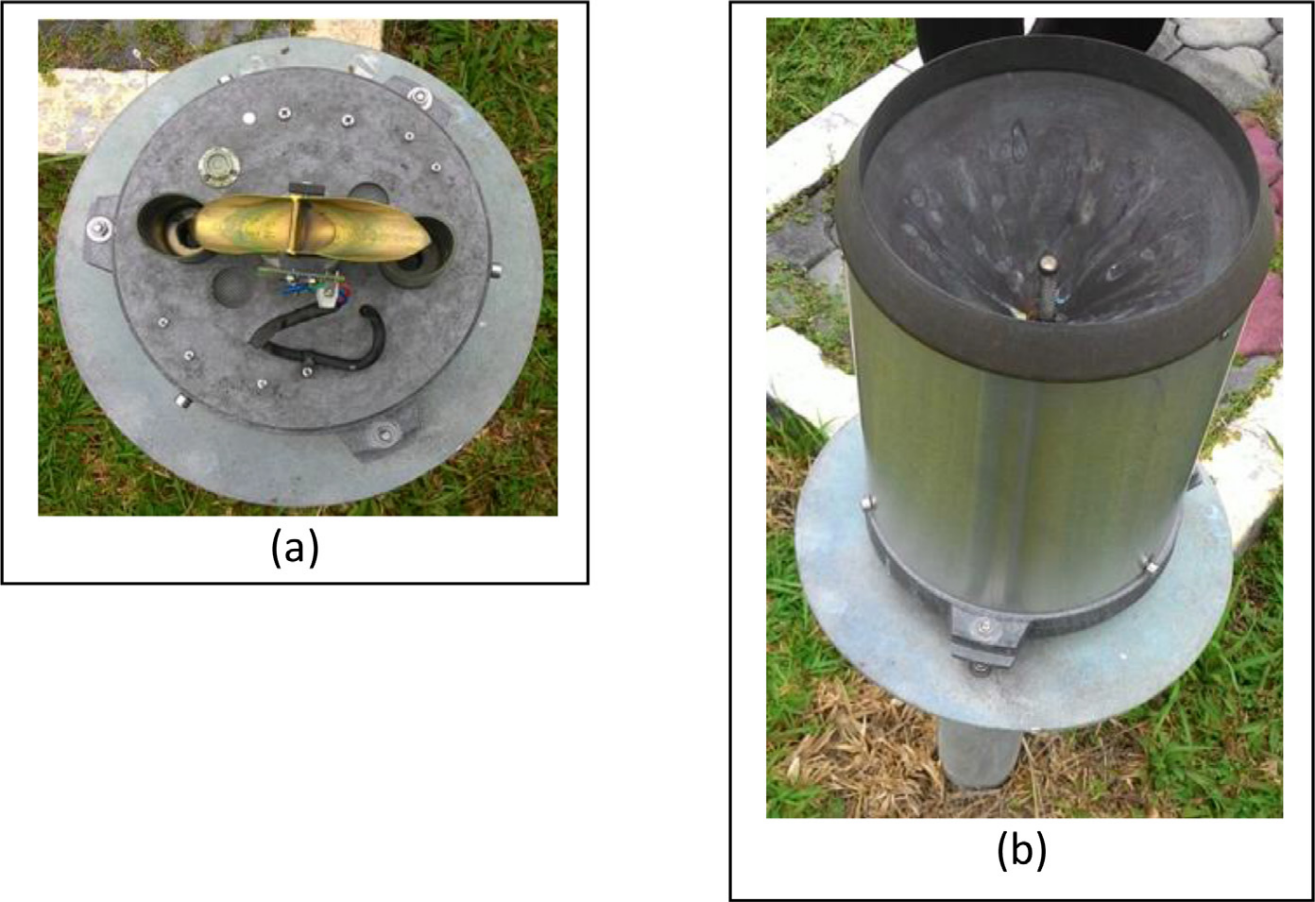
Autonomous Tipping bucket inside top view (a) and angle side view (b).

**Fig. 11:**
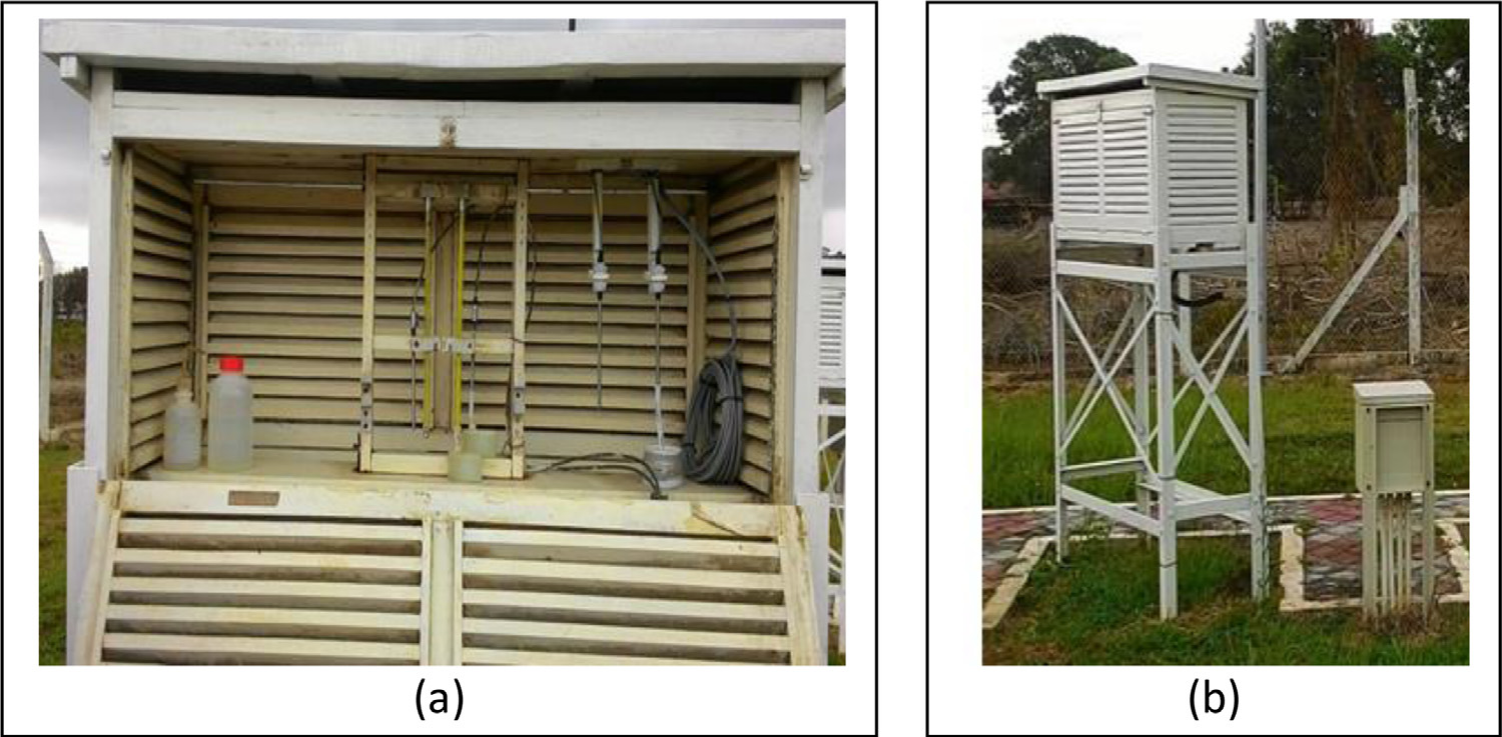
Autonomous thermometer screen inside view (a) and side view (b).

Aside from that, all meteorology instruments were consistently scheduled to be calibrated twice yearly. While the focus of this exercise was only on the three parameters mentioned earlier, it is undeniable that all meteorology parameters gathered by the weather station would be associated at some level to establish a specific climate condition. Having said that, it was considered safe to make a premature inference that any huge fluctuation from any parameter would contribute to a significant correlation, either positive or negative. Figs. 9-11 show the AWS available at the Batu Berendam's weather station, the autonomous tipping bucket, and the autonomous thermometer screen, respectively.

Utilising the typical weather data via TRY is implied as a reliable source, chosen to represent the pattern of description for the weather environment at a specific location [1], [2], [3], [4]. In this article, Finkelstein–Schafer statistic (F-S statistic) was utilised to generate TRY. First and foremost, the CDF for the individual month and the long-term CDF was calculated. After that, using Equation (1) the difference between the long-term CDF and the individual month CDF of interest was calculated. Lastly, the smallest value of the FS statistic of each individual month was selected to form a TRY. The TRY formed was then utilised for further statistical analysis, as mentioned previously [3], [5].

(1) $$FS=\left(1/n\right)\sum_{i=1}^{n}\left|\delta_{i}\right|$$

Using the open-source statistical software proved to be convenient for the ease of data handling and data treatment. Thus, the JASP software for statistical analysis was employed to perform the descriptive analysis and the correlation analysis. The descriptive analysis included all crucial information such as the mean, standard deviation, skewness, kurtosis, maximum and minimum value [6]. Furthermore, for the correlation analysis, the set of correlation measures such as Pearson's r, Spearman's rho and Kendall's tau was undertaken with the support of Bayesian correlation pair analysis consecutively. Two sets of analysis were conducted, the first set (Correlation analysis Set A) involved was n = 12 which is between the TRY representing the typical weather data and the best year (January-December 2010) for electricity consumption while another set (Correlation analysis Set B) involved was n = 108 with repetitive TRY, which was employed against the whole raw data set of electricity consumption (January 2010-December 2018).

## Ethics Statement

The authors declare that the work does not involve the use of human subject or animal experiments which need for any informed consent.

## Declaration of Competing Interest

The authors declare that they have no known competing financial interests or personal relationships which have or could be perceived to have influenced the work reported in this article.

## Appendix Supplementary materials

Image, application 1

Image, application 2
