# Supplementary material for: Data Assessment on the relationship between typical weather data and electricity consumption of academic building in Melaka
Source: Data Brief. 2021 Feb 1;35:106797. doi: 10.1016/j.dib.2021.106797 (PMC7881228; doi:10.1016/j.dib.2021.106797)

Report TRY VS Electricity 2010-2018

Descriptive Statistics

Descriptive Statistics

	Temperature	Relative humidity	Rainfall	Electricity Consumption
**Valid**	108	108	108	108
**Missing**	0	0	0	0
**Mean**	27.861	77.270	8.150	1.483e +6
**Std. Error of Mean**	0.040	0.010	0.254	17241.897
**Median**	27.915	77.300	7.565	1.502e +6
**Mode** [a]	27.303	77.300	3.845	989502.000
**Std. Deviation**	0.419	0.100	2.644	179183.053
**IQR**	0.620	0.000	2.290	269058.500
**Variance**	0.175	0.010	6.988	3.211e +10
**Skewness**	-0.457	-3.058	0.871	-0.423
**Std. Error of Skewness**	0.233	0.233	0.233	0.233
**Kurtosis**	-1.070	7.488	0.715	-0.442
**Std. Error of Kurtosis**	0.461	0.461	0.461	0.461
**Minimum**	27.113	76.939	3.845	989502.000
**Maximum**	28.419	77.300	14.477	1.786e +6
**Sum**	3009.015	8345.151	880.209	1.602e +8
**25th percentile**	27.550	77.300	6.691	1.360e +6
**50th percentile**	27.915	77.300	7.565	1.502e +6
**75th percentile**	28.171	77.300	8.981	1.629e +6

[a] More than one mode exists, only the first is reported

Distribution Plots

Temperature

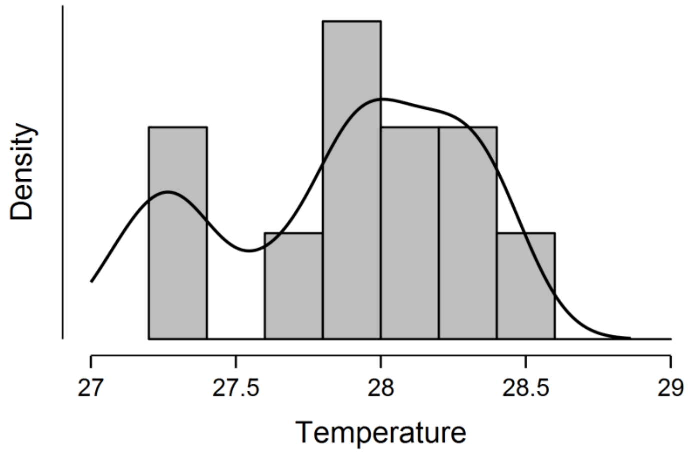

Relative humidity

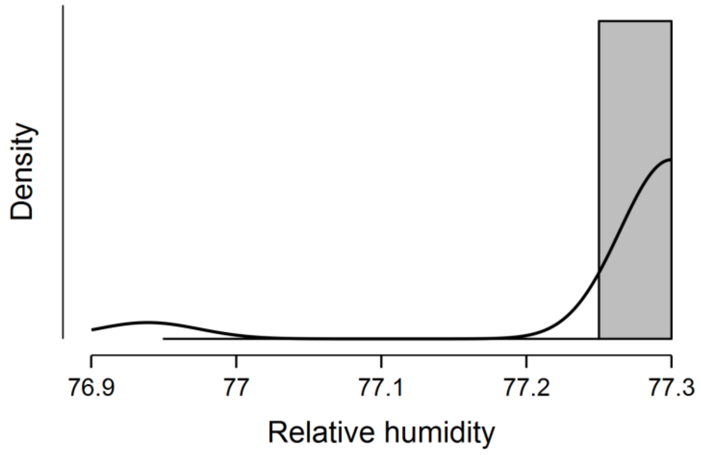

Rainfall

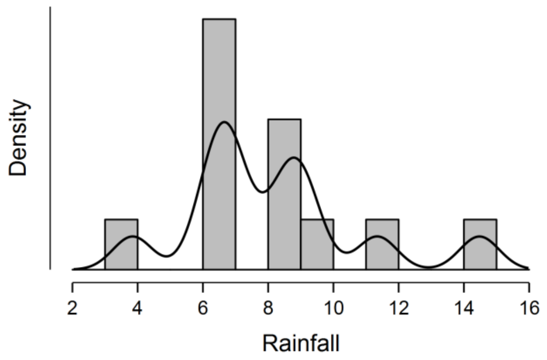

Electricity Consumption

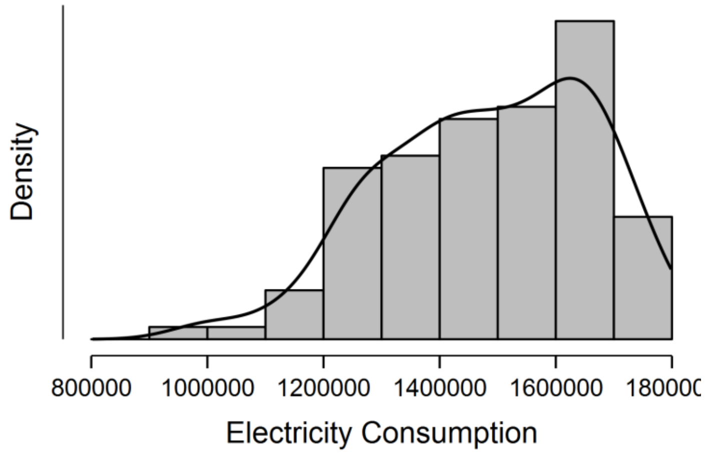

Correlation Matrix

Correlation Table

		Temperature	Relative humidity	Rainfall	Electricity Consumption
Temperature	Pearson's r	—			
	p-value	—			
	Spearman's rho	—			
	p-value	—			
	Kendall's tau B	—			
	p-value	—			
Relative humidity	Pearson's r	-0.404	—		
	p-value	< .001	—		
	Spearman's rho	-0.481	—		
	p-value	< .001	—		
	Kendall's tau B	-0.411	—		
	p-value	< .001	—		
Rainfall	Pearson's r	0.169	-0.067	—	
	p-value	0.081	0.488	—	
	Spearman's rho	0.137	-0.131	—	
	p-value	0.159	0.177	—	
	Kendall's tau B	0.076	-0.111	—	
	p-value	0.278	0.175	—	
Electricity Consumption	Pearson's r	-0.131	0.043	-0.158	—
	p-value	0.177	0.660	0.102	—
	Spearman's rho	-0.096	0.044	-0.153	—
	p-value	0.325	0.655	0.115	—
	Kendall's tau B	-0.064	0.036	-0.110	—
	p-value	0.349	0.653	0.104	—

Correlation Plot

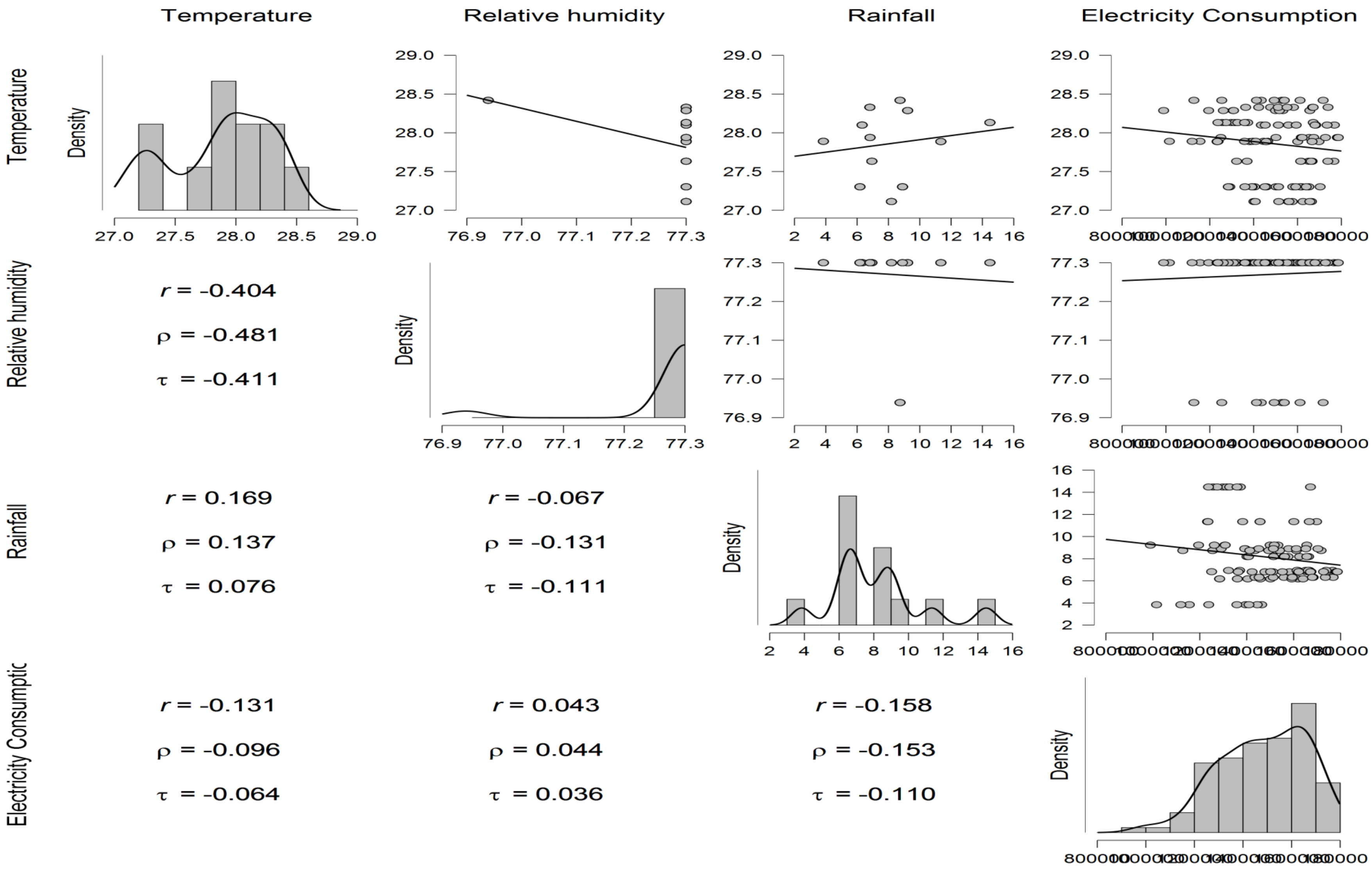

Bayesian Correlation Pairs

Bayesian Pearson Correlation

		r	BF_{10}
Temperature	- Relative humidity	-0.404	1249.659
Temperature	- Rainfall	0.169	0.542
Temperature	- Electricity Consumption	-0.131	0.295
Relative humidity	- Rainfall	-0.067	0.153
Relative humidity	- Electricity Consumption	0.043	0.132
Rainfall	- Electricity Consumption	-0.158	0.448

Plots

Temperature - Relative humidity

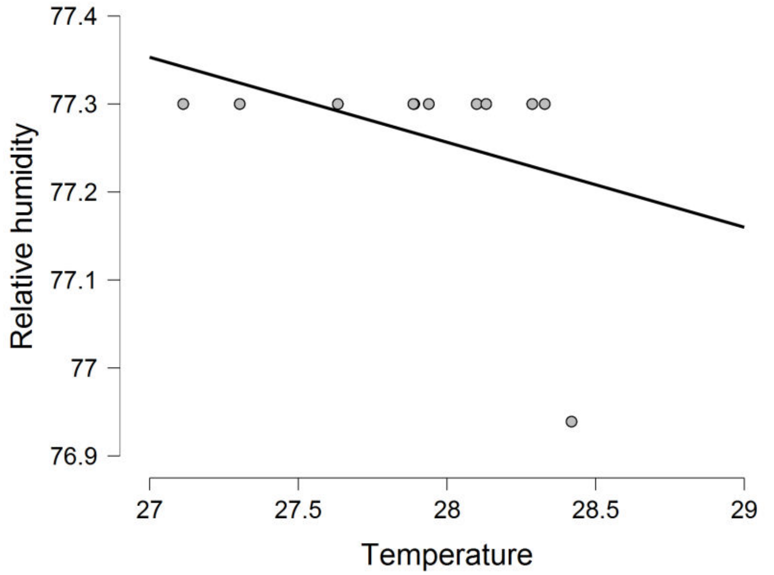

Temperature – Rainfall

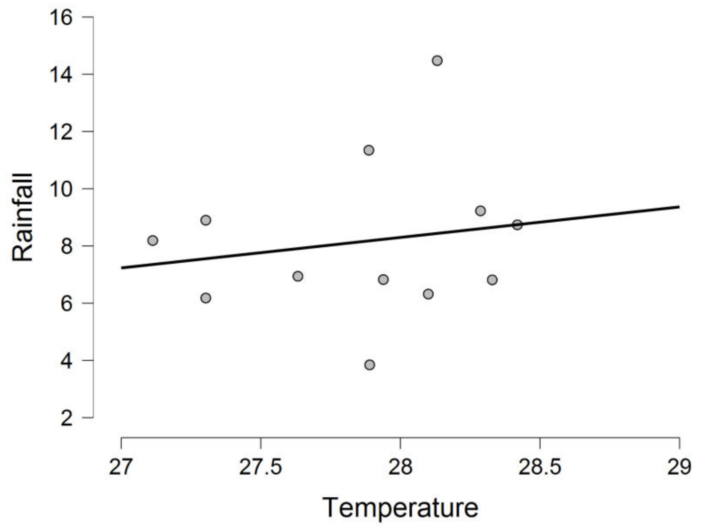

Temperature - Electricity Consumption

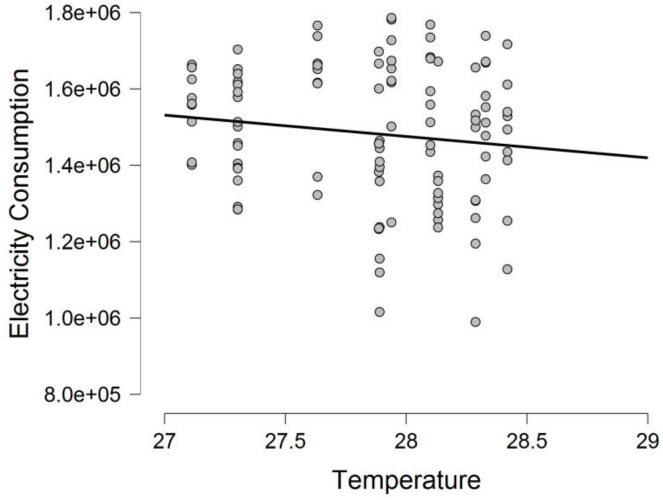

Relative humidity - Rainfall

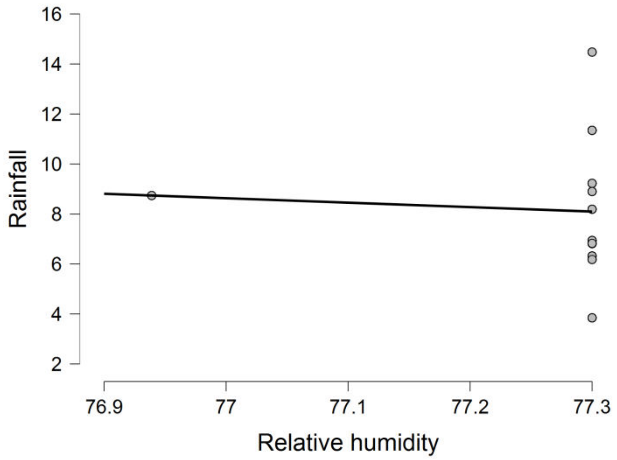

Relative humidity - Electricity Consumption

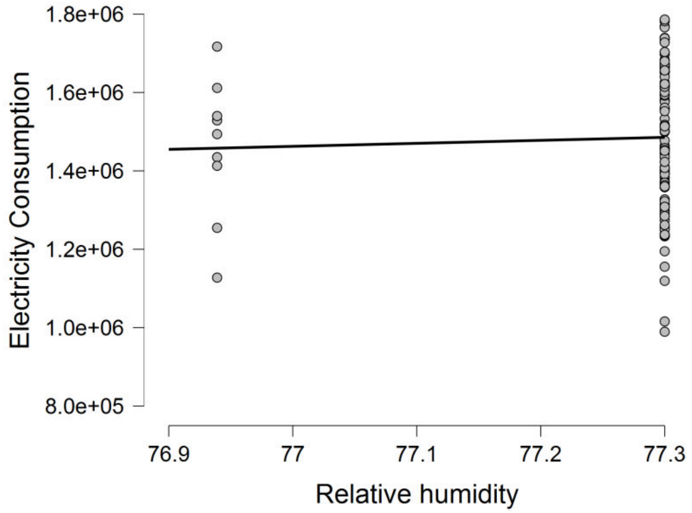

Rainfall - Electricity Consumption

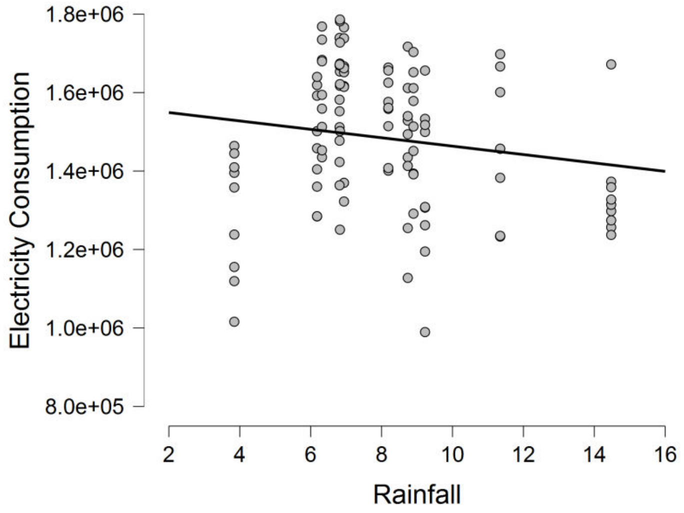

Supplement: Supplementary file 1 [file mmc1.zip › report TRY vs electricity 2010-2018.pdf]
