# Supplementary material for: Data Assessment on the relationship between typical weather data and electricity consumption of academic building in Melaka
Source: Data Brief. 2021 Feb 1;35:106797. doi: 10.1016/j.dib.2021.106797 (PMC7881228; doi:10.1016/j.dib.2021.106797)

Report Ultimate Weather Data Electricity

Descriptive Statistics

Descriptive Statistics

	Temperature	Relative humidity	Rainfall	Electricity consumption
**Valid**	12	12	12	12
**Missing**	0	0	0	0
**Mean**	27.861	77.270	8.150	1.573e +6
**Std. Error of Mean**	0.126	0.030	0.793	40891.553
**Median**	27.915	77.300	7.565	1.618e +6
**Mode** [a]	27.303	77.300	3.845	1.383e +6
**Std. Deviation**	0.435	0.104	2.748	141652.495
**IQR**	0.620	0.000	2.290	210975.250
**Variance**	0.189	0.011	7.553	2.007e +10
**Skewness**	-0.518	-3.464	0.987	-0.101
**Std. Error of Skewness**	0.637	0.637	0.637	0.637
**Kurtosis**	-0.976	12.000	1.731	-1.519
**Std. Error of Kurtosis**	1.232	1.232	1.232	1.232
**Minimum**	27.113	76.939	3.845	1.383e +6
**Maximum**	28.419	77.300	14.477	1.768e +6
**Sum**	334.335	927.239	97.801	1.887e +7
**25th percentile**	27.550	77.300	6.691	1.449e +6
**50th percentile**	27.915	77.300	7.565	1.618e +6
**75th percentile**	28.171	77.300	8.981	1.660e +6

[a] More than one mode exists, only the first is reported

Distribution Plots

Temperature

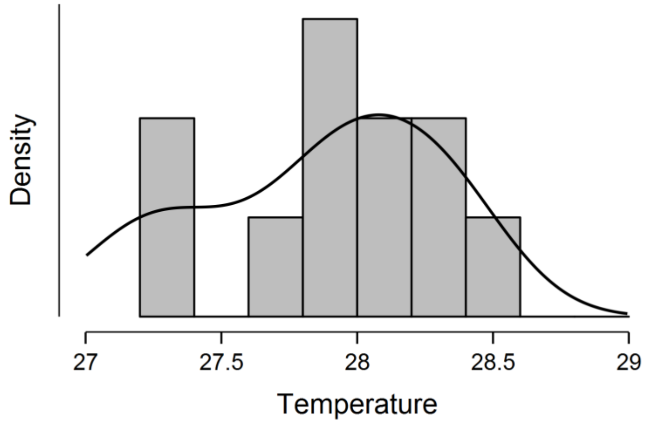

Relative humidity

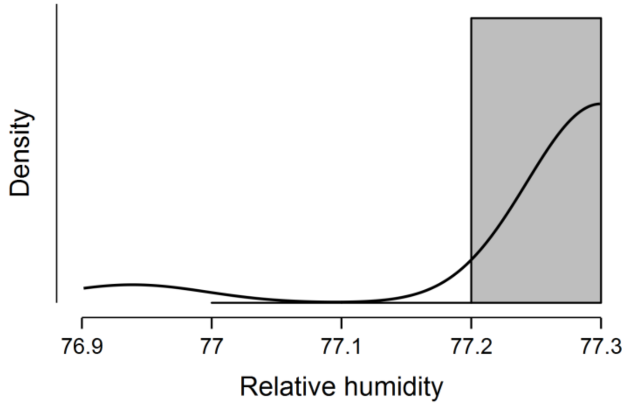

Rainfall

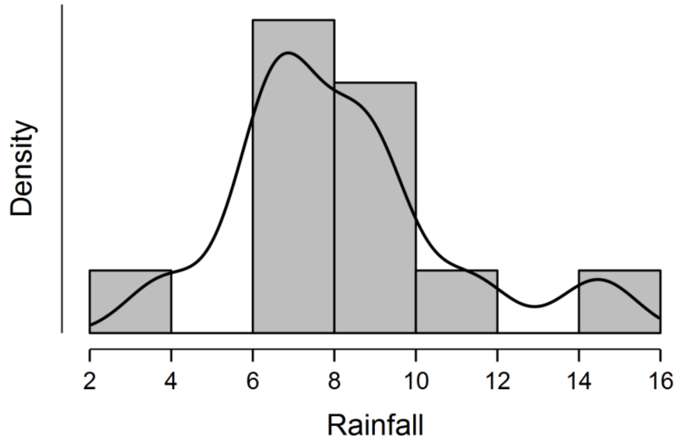

Electricity consumption

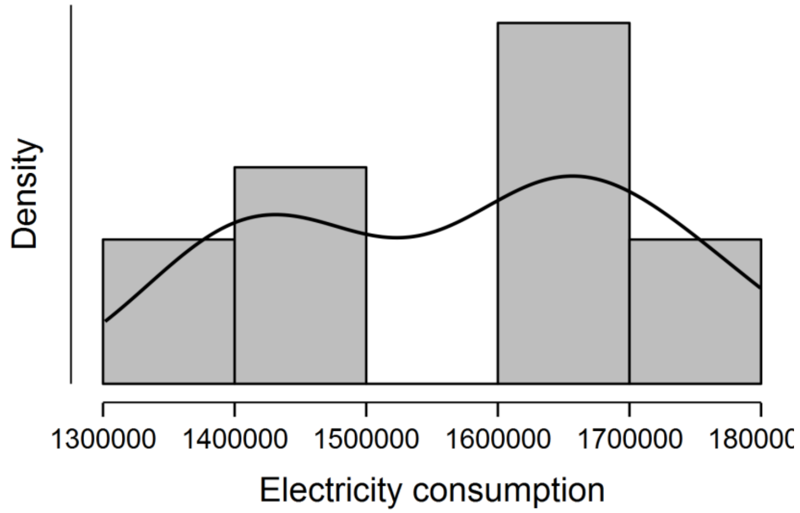

Correlation Matrix

Correlation Table

		Temperature	Relative humidity	Rainfall	Electricity consumption
Temperature	Pearson's r	—			
	p-value	—			
	Spearman's rho	—			
	p-value	—			
	Kendall's tau B	—			
	p-value	—			
Relative humidity	Pearson's r	-0.404	—		
	p-value	0.193	—		
	Spearman's rho	-0.481	—		
	p-value	0.113	—		
	Kendall's tau B	-0.411	—		
	p-value	0.110	—		
Rainfall	Pearson's r	0.169	-0.067	—	
	p-value	0.600	0.835	—	
	Spearman's rho	0.137	-0.131	—	
	p-value	0.672	0.685	—	
	Kendall's tau B	0.076	-0.111	—	
	p-value	0.731	0.664	—	
Electricity consumption	Pearson's r	0.287	-0.086	0.053	—
	p-value	0.365	0.791	0.870	—
	Spearman's rho	0.312	0.044	0.007	—
	p-value	0.324	0.893	0.991	—
	Kendall's tau B	0.198	0.037	0.000	—
	p-value	0.372	0.885	1.000	—

Correlation Plot

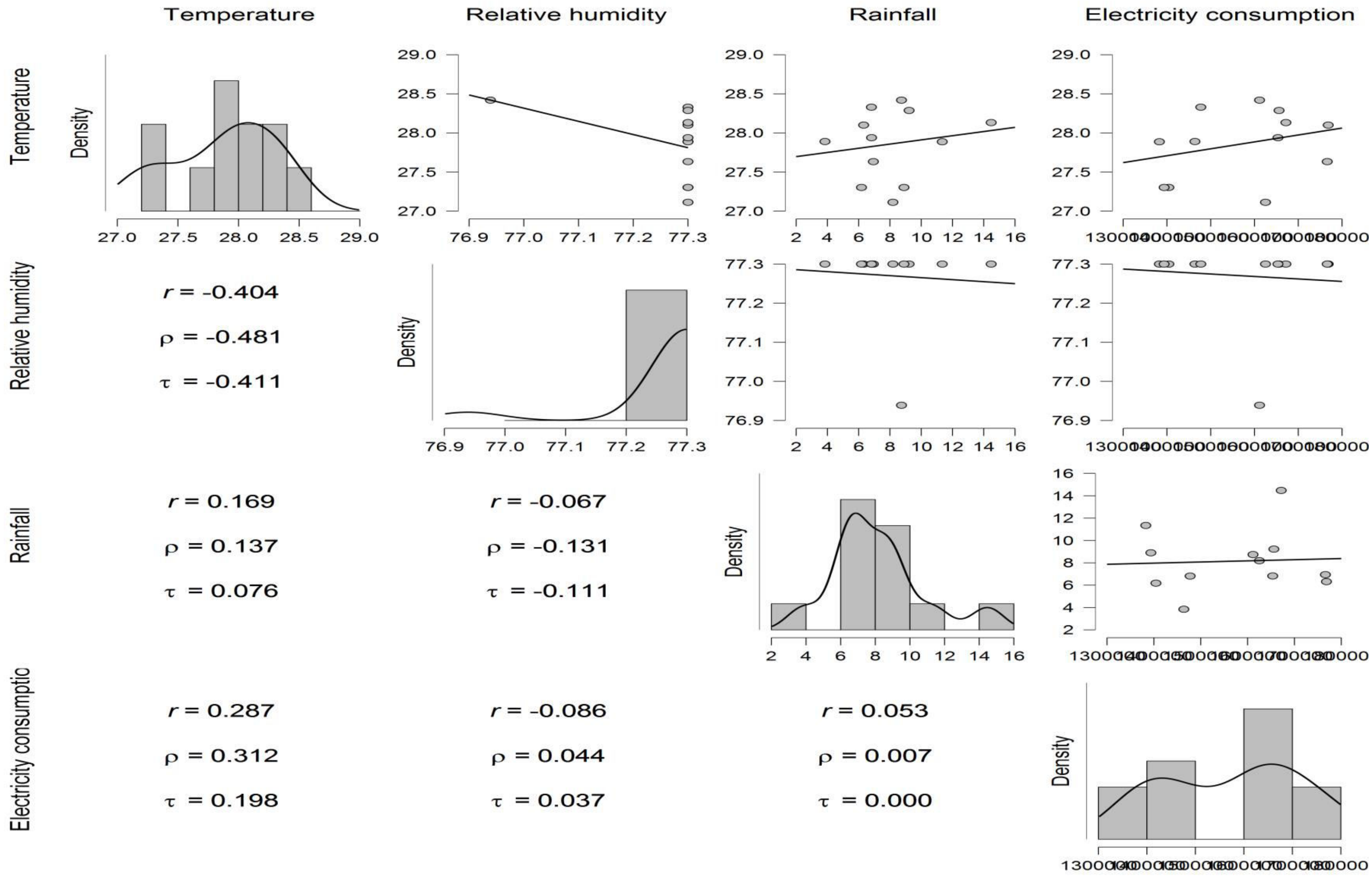

Bayesian Correlation Pairs

Bayesian Pearson Correlation

		r	BF_{10}
Temperature	- Relative humidity	-0.404	0.762
Temperature	- Rainfall	0.169	0.401
Temperature	- Electricity consumption	0.287	0.514
Relative humidity	- Rainfall	-0.067	0.361
Relative humidity	- Electricity consumption	-0.086	0.366
Rainfall	- Electricity consumption	0.053	0.359

Plots

Temperature - Relative humidity

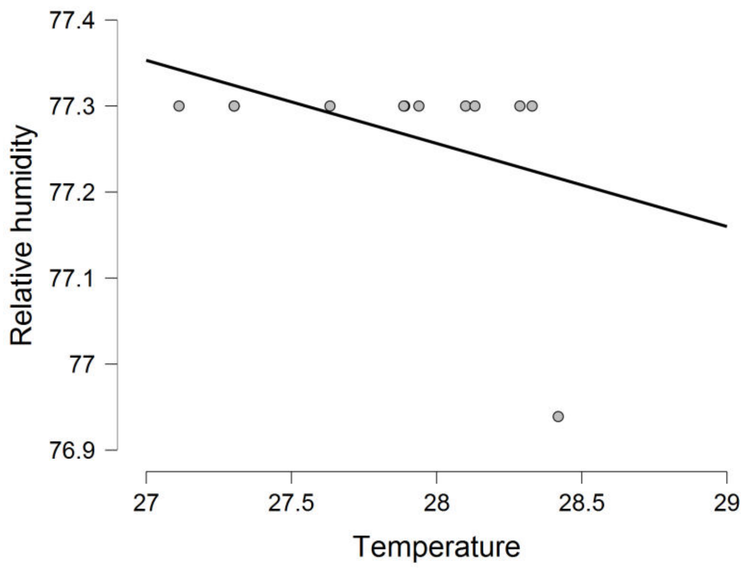

Temperature - Rainfall

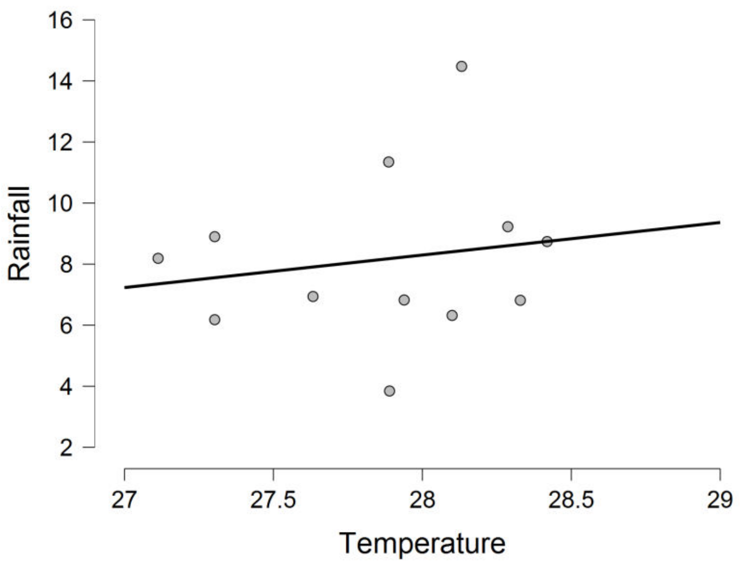

Temperature - Electricity consumption

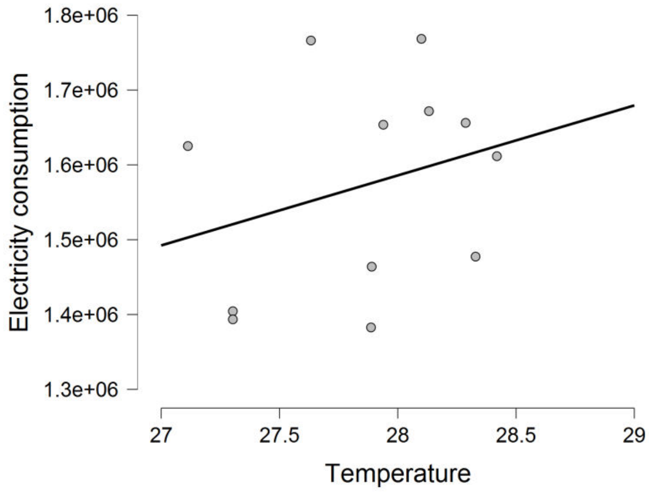

Relative humidity - Rainfall

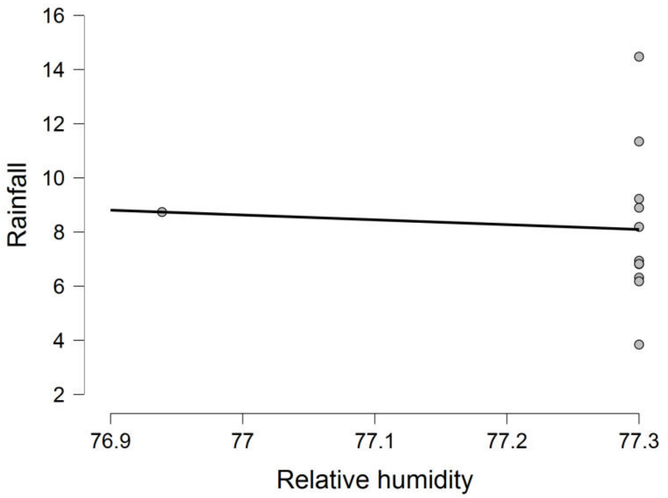

Relative humidity - Electricity consumption

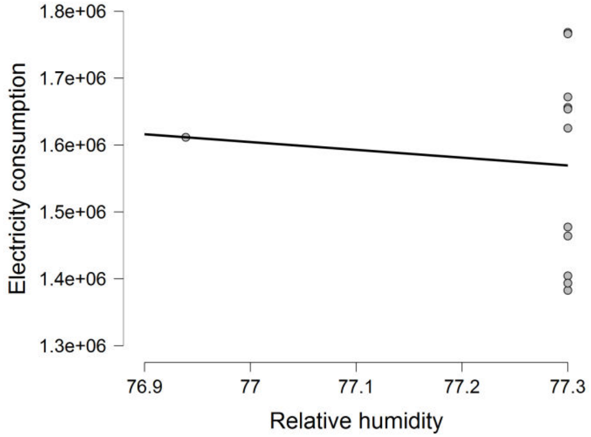

Rainfall - Electricity consumption

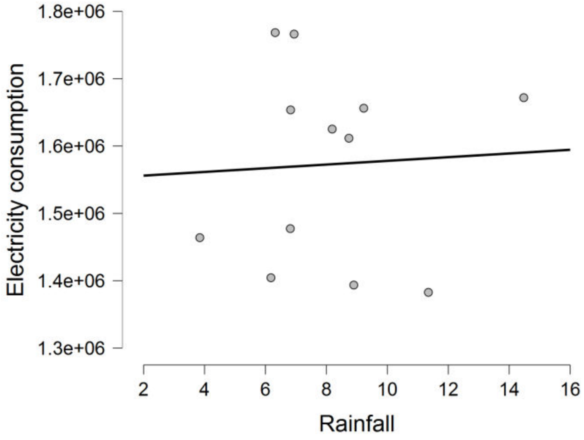

Supplement: Supplementary file 1 [file mmc1.zip › report ultimate weather data electricity.pdf]
