# Supplementary figures and images for: Data Assessment on the relationship between typical weather data and electricity consumption of academic building in Melaka
Source: Data Brief. 2021 Feb 1;35:106797. doi: 10.1016/j.dib.2021.106797 (PMC7881228; doi:10.1016/j.dib.2021.106797)

## Compilation 1: Graph Best Worst Electricity Consumption

Electricity consumption

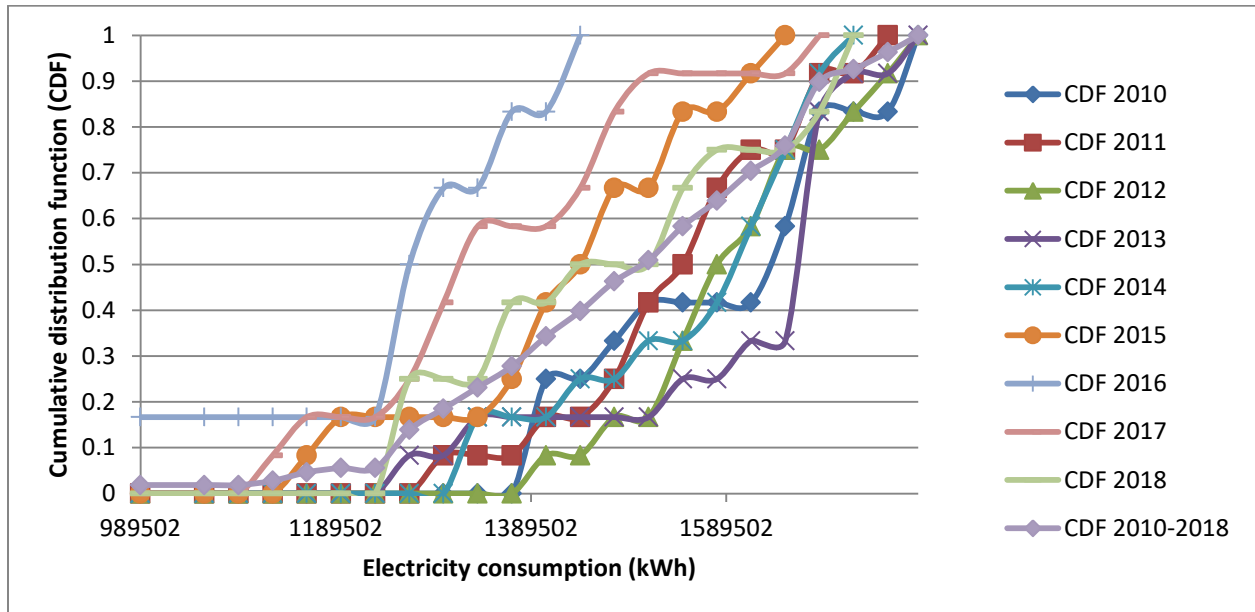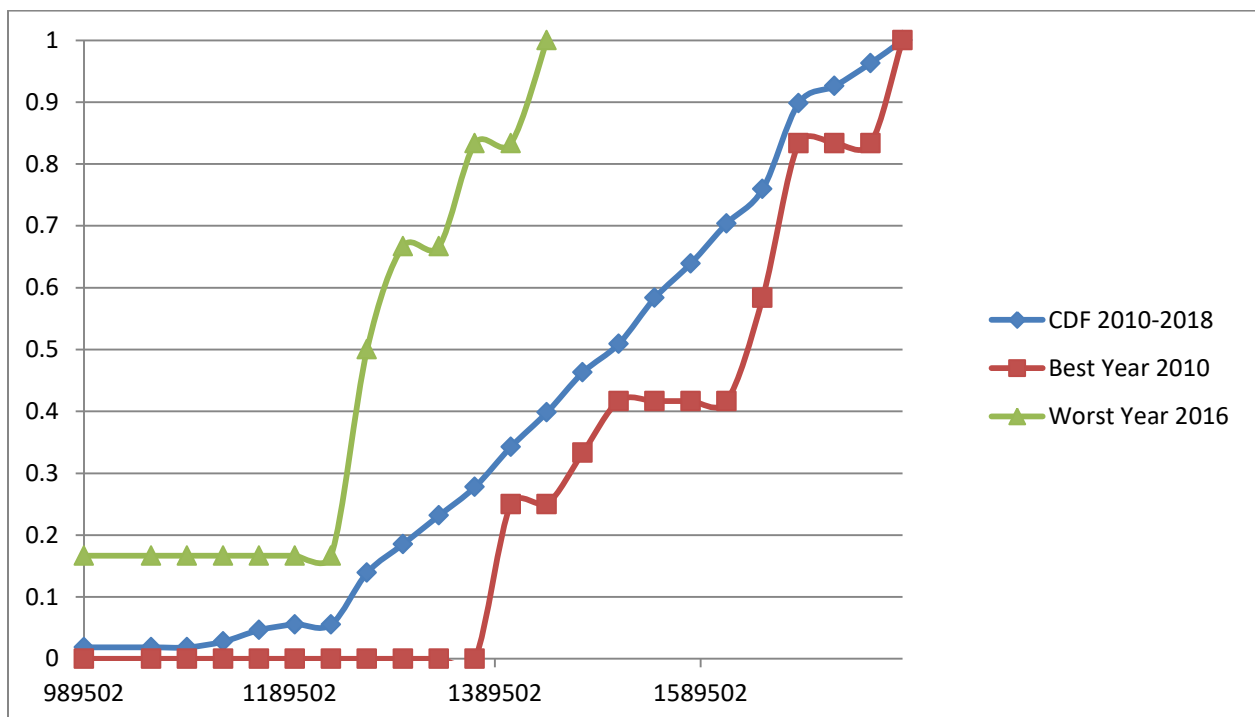

Supplement: Supplementary file 1 [file mmc1.zip › graph best worst electricity consumption.pdf]

Compilation 2: Graph CDF Best and Worst Weather Data

Temperature

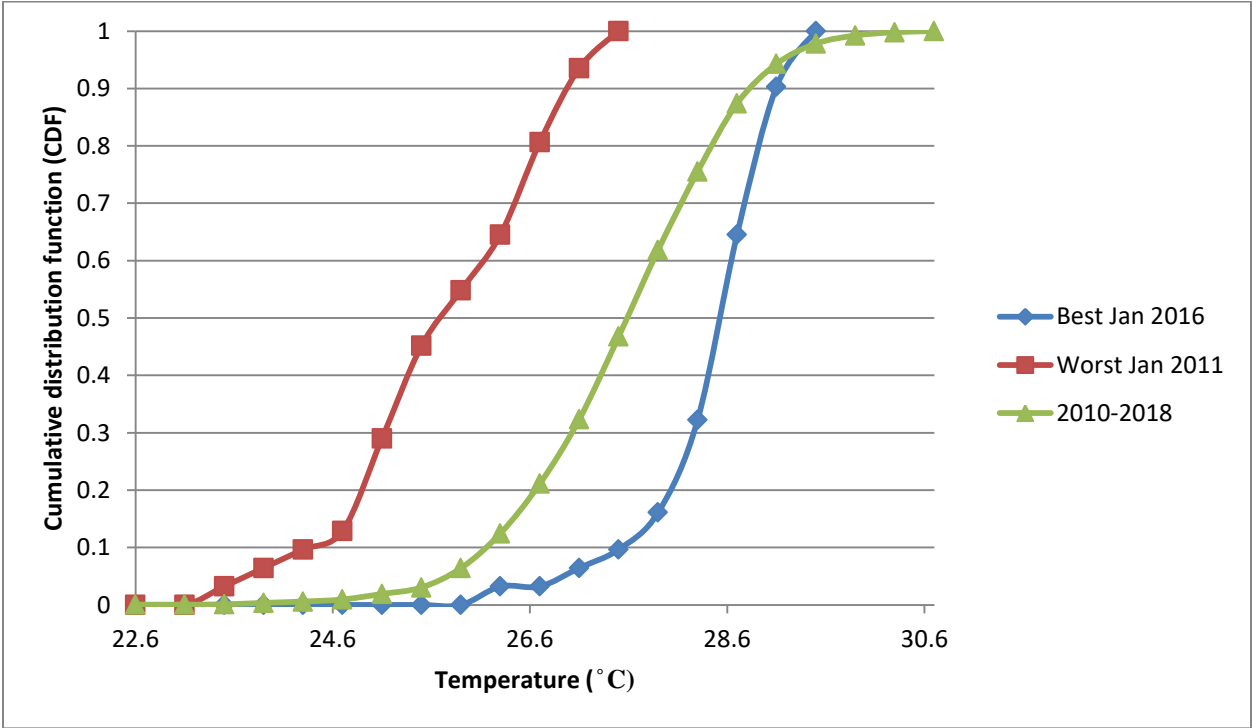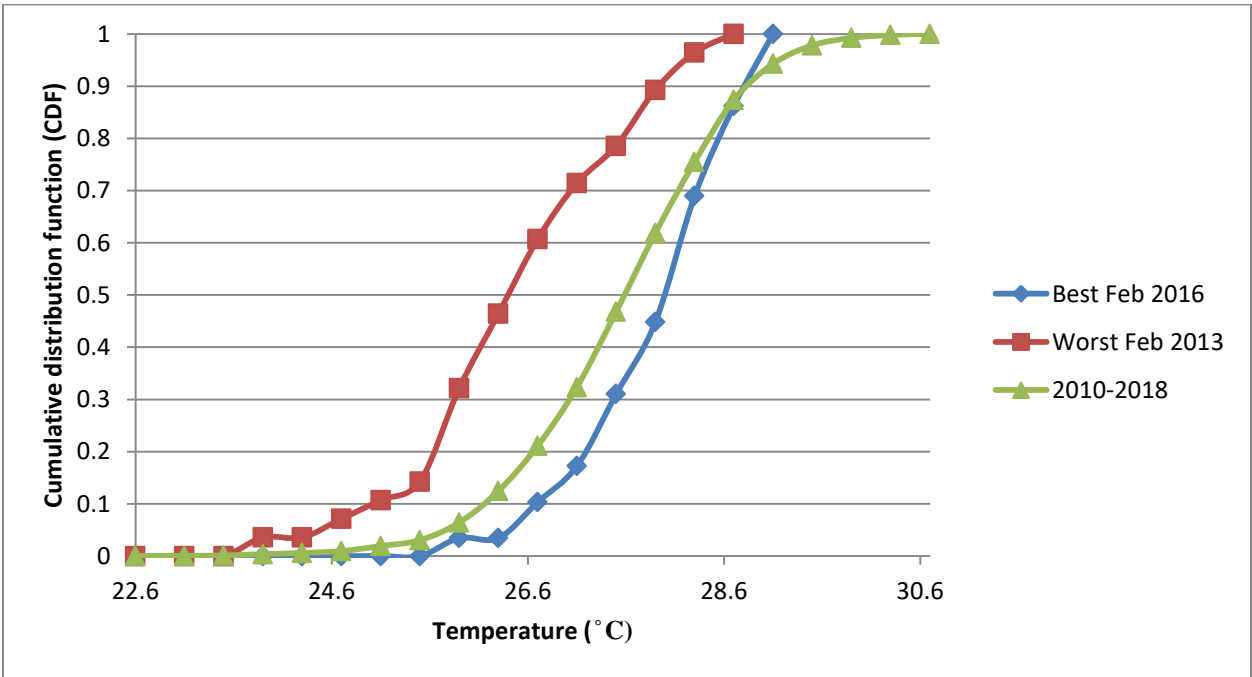

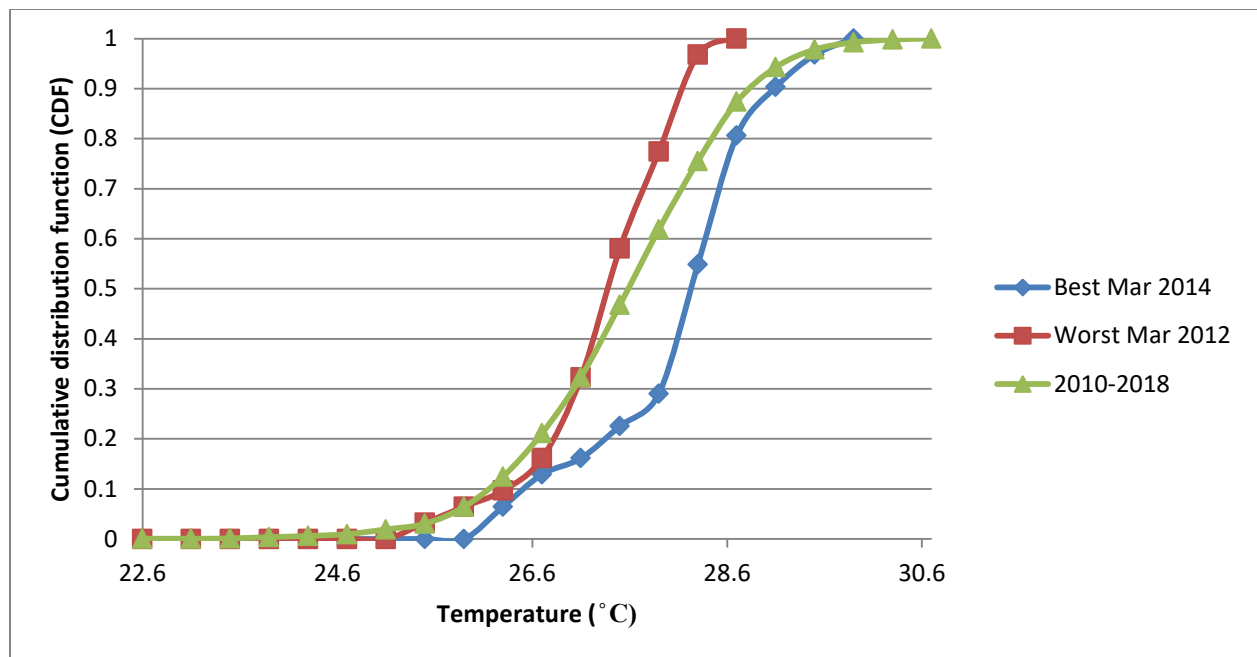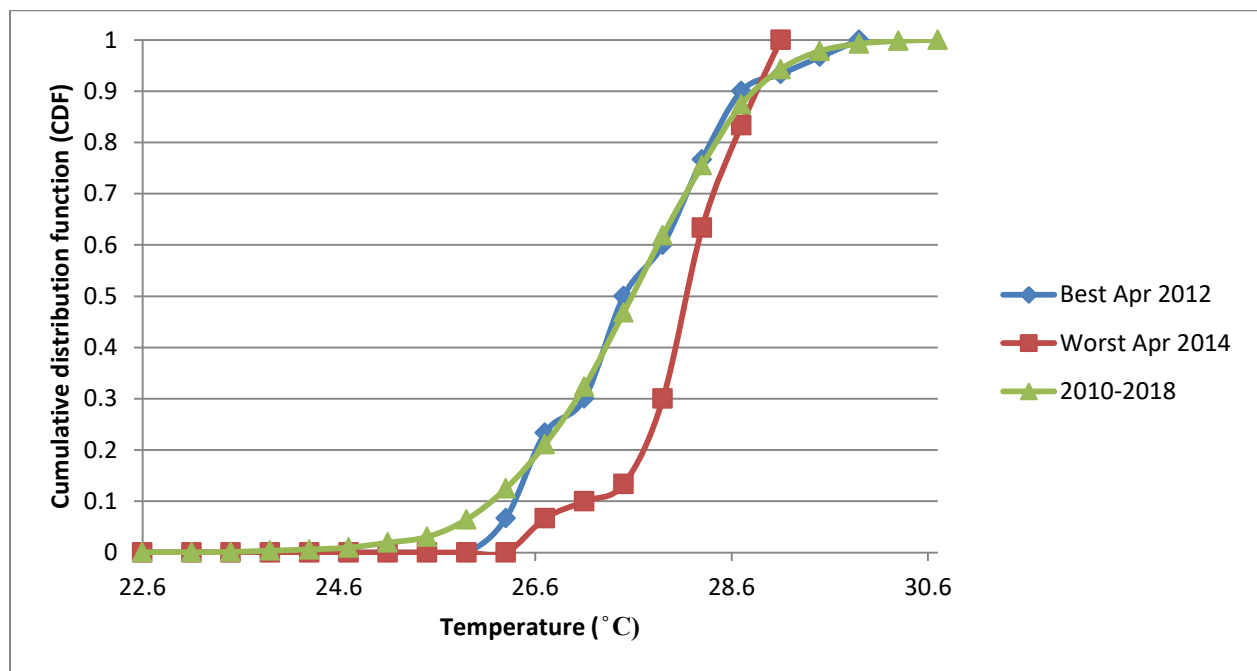

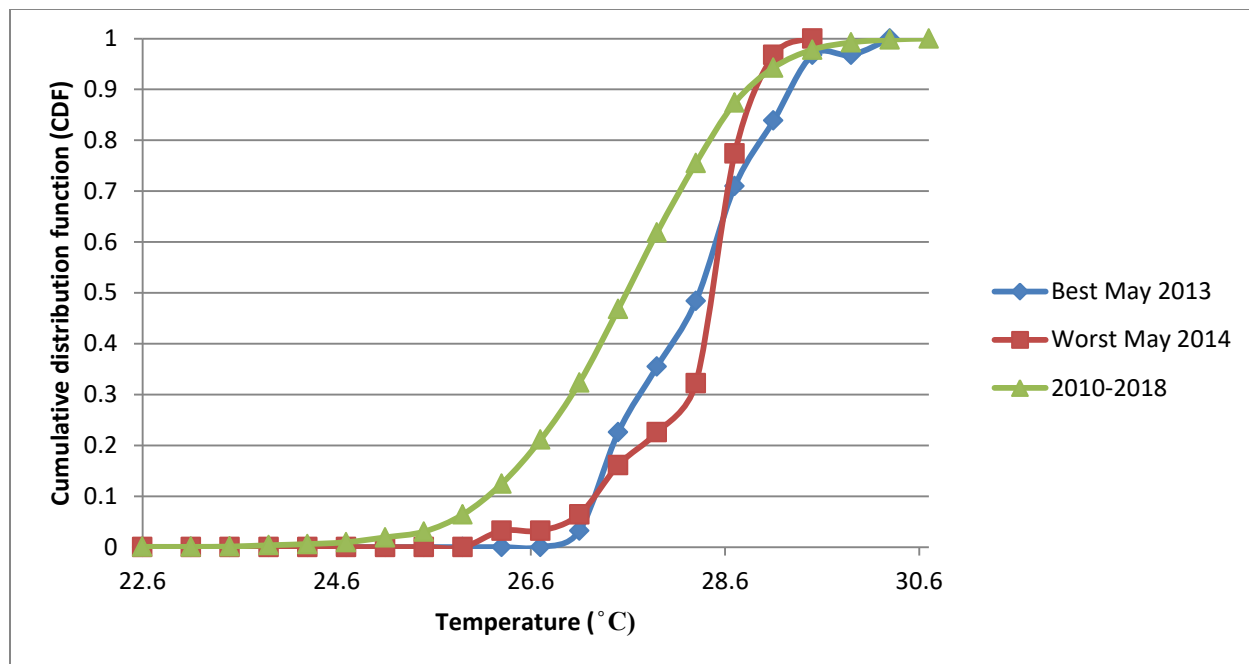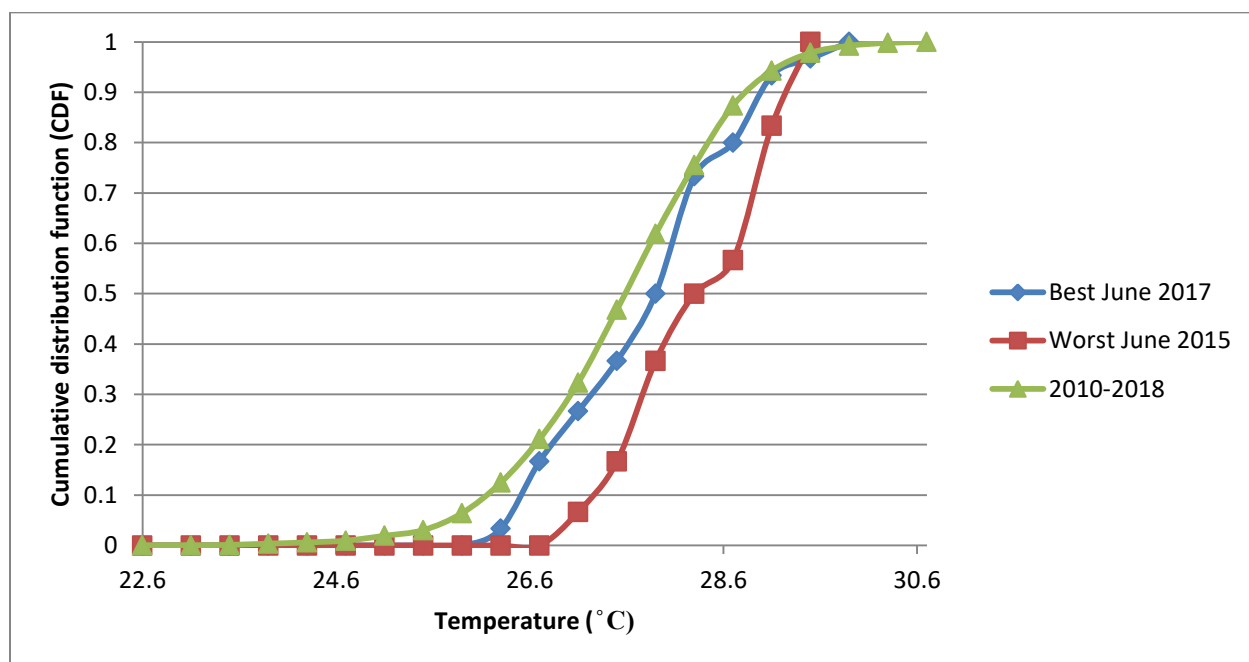

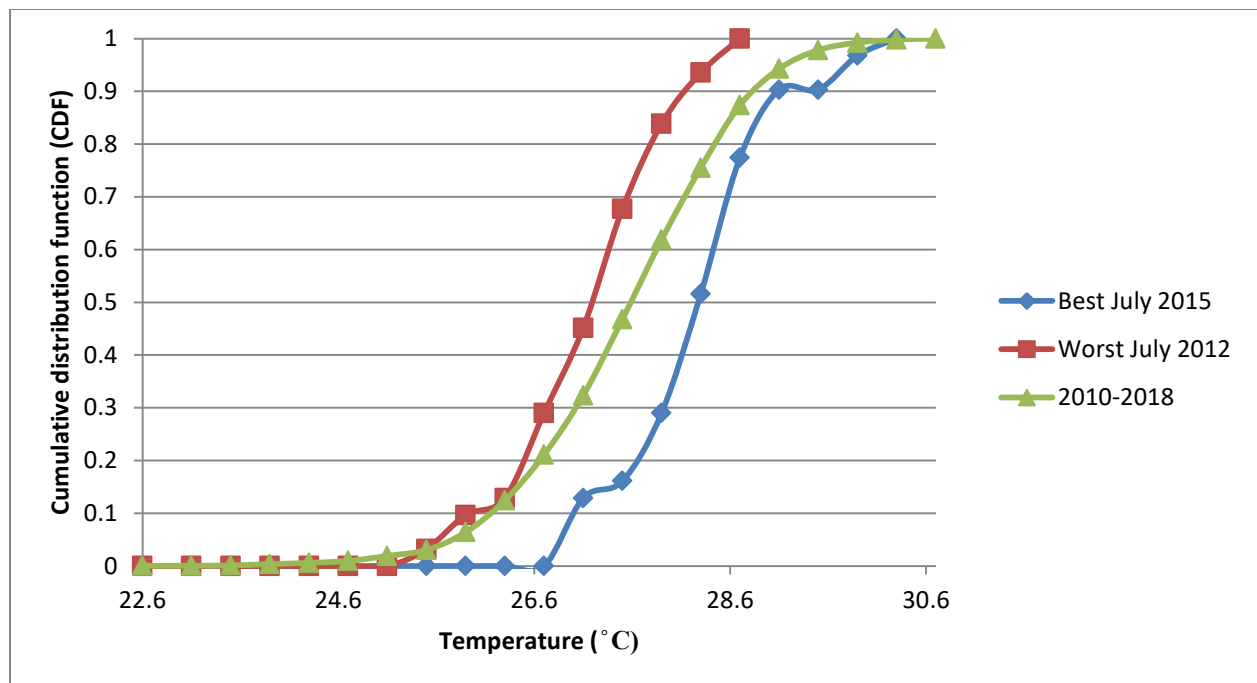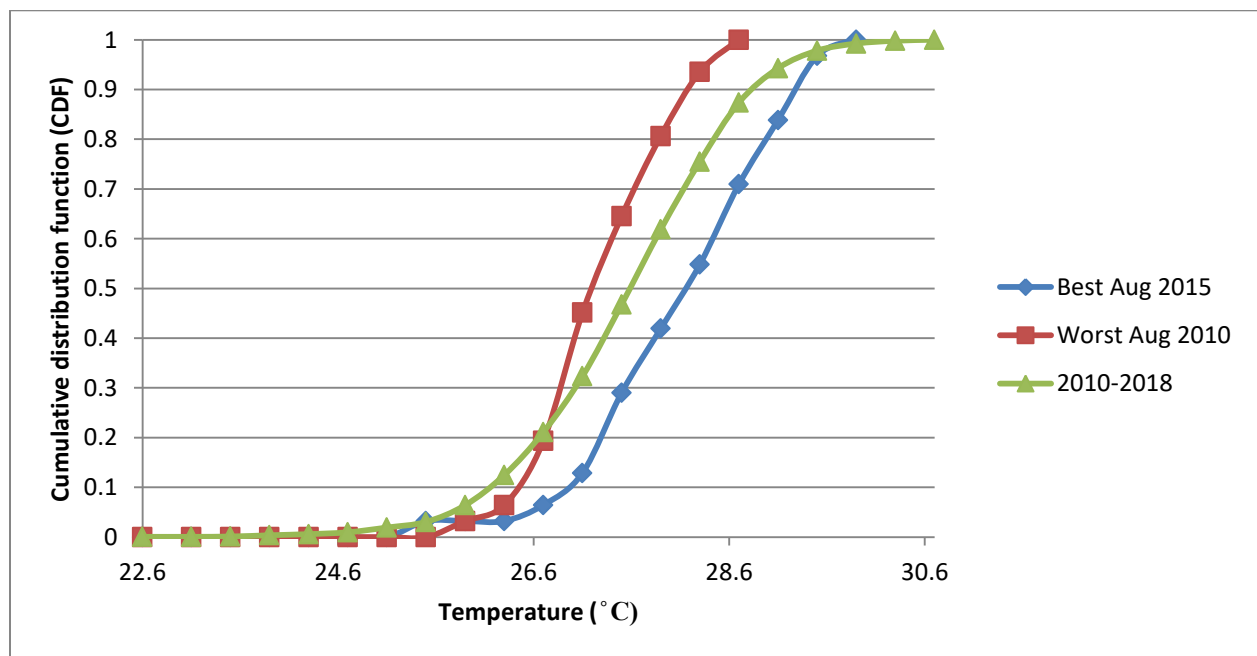

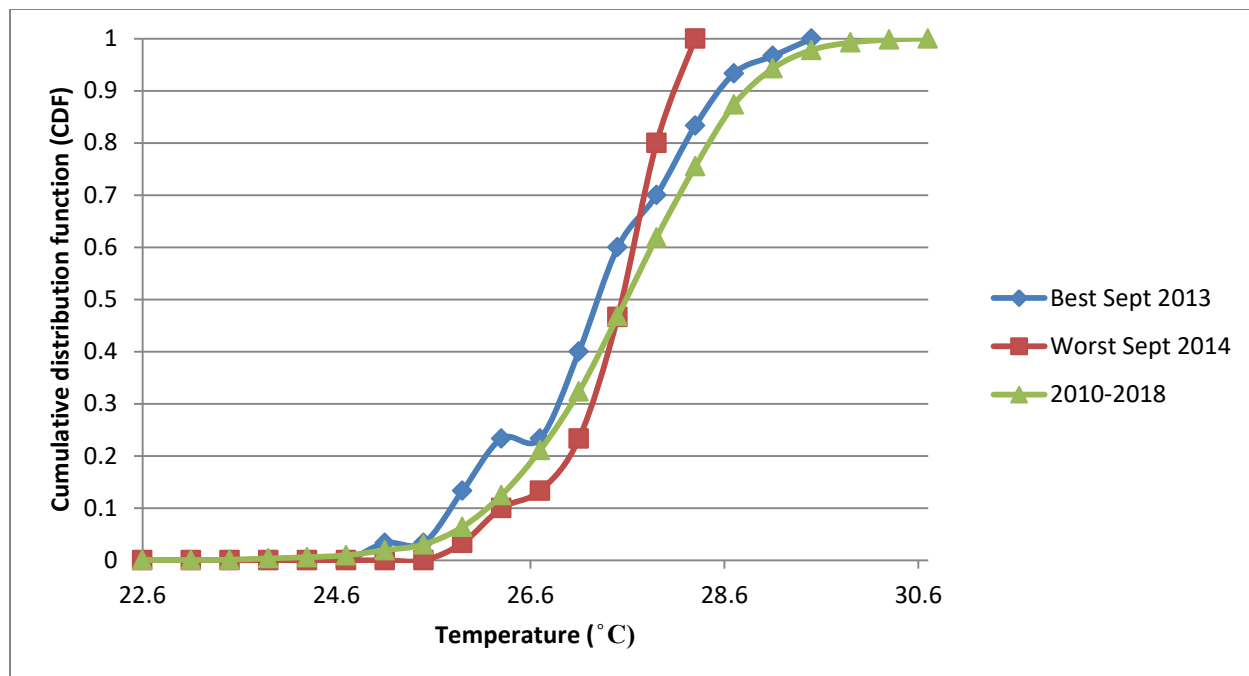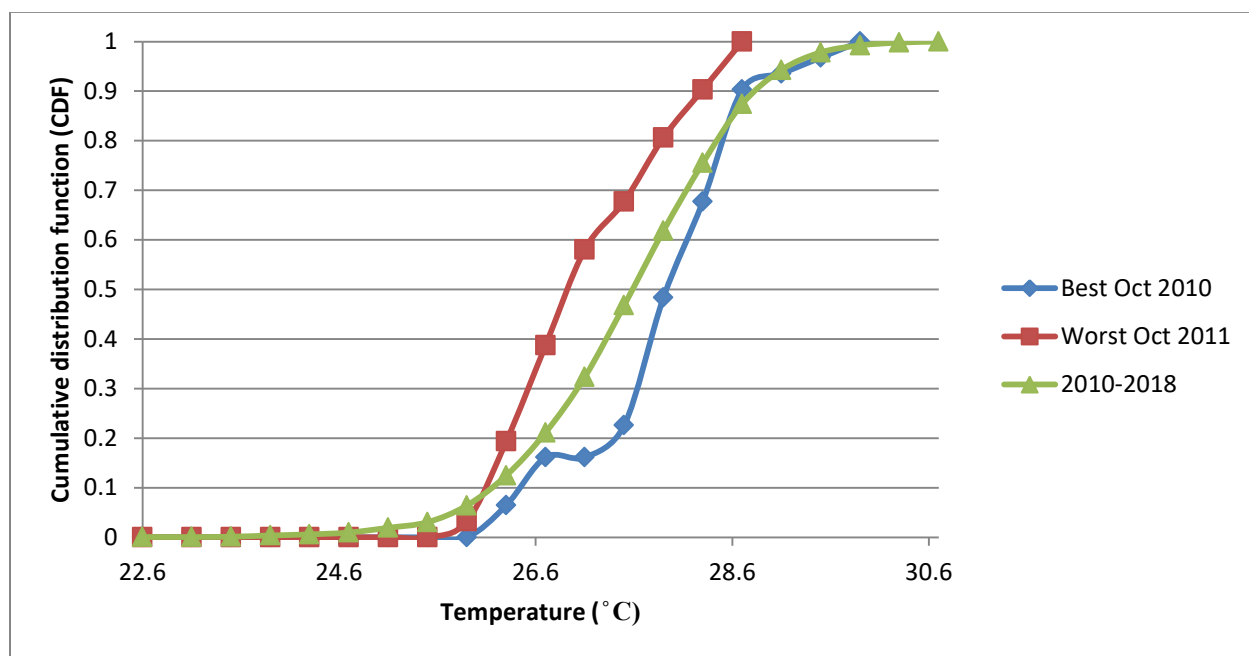

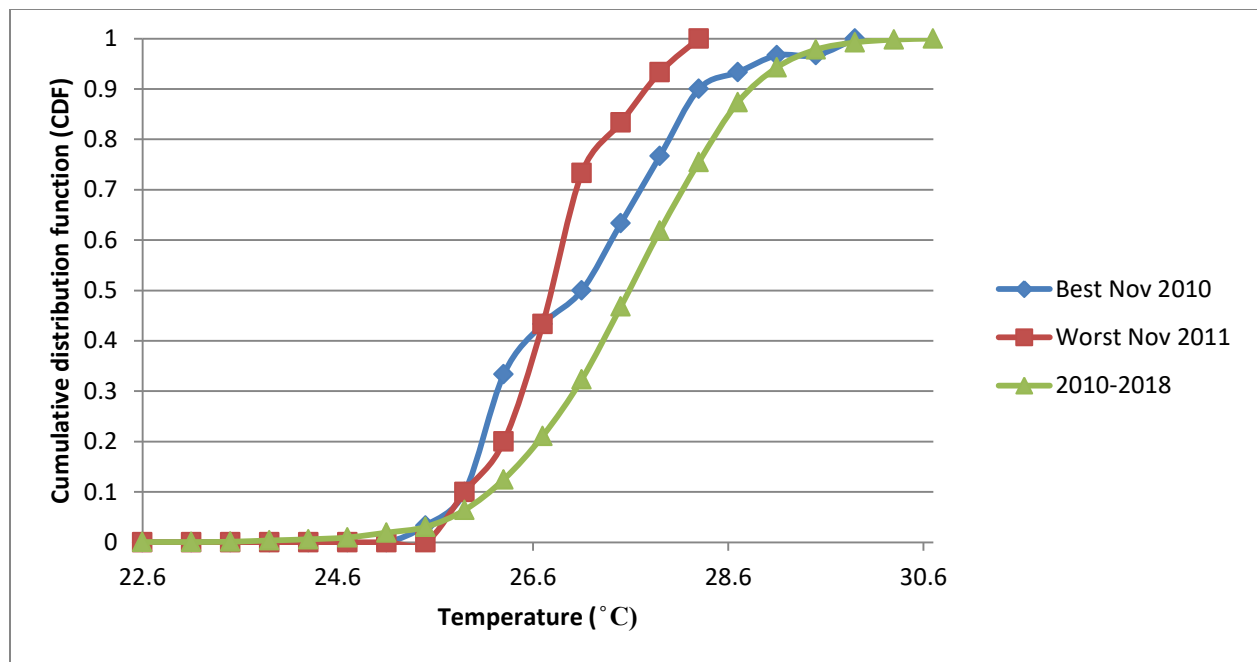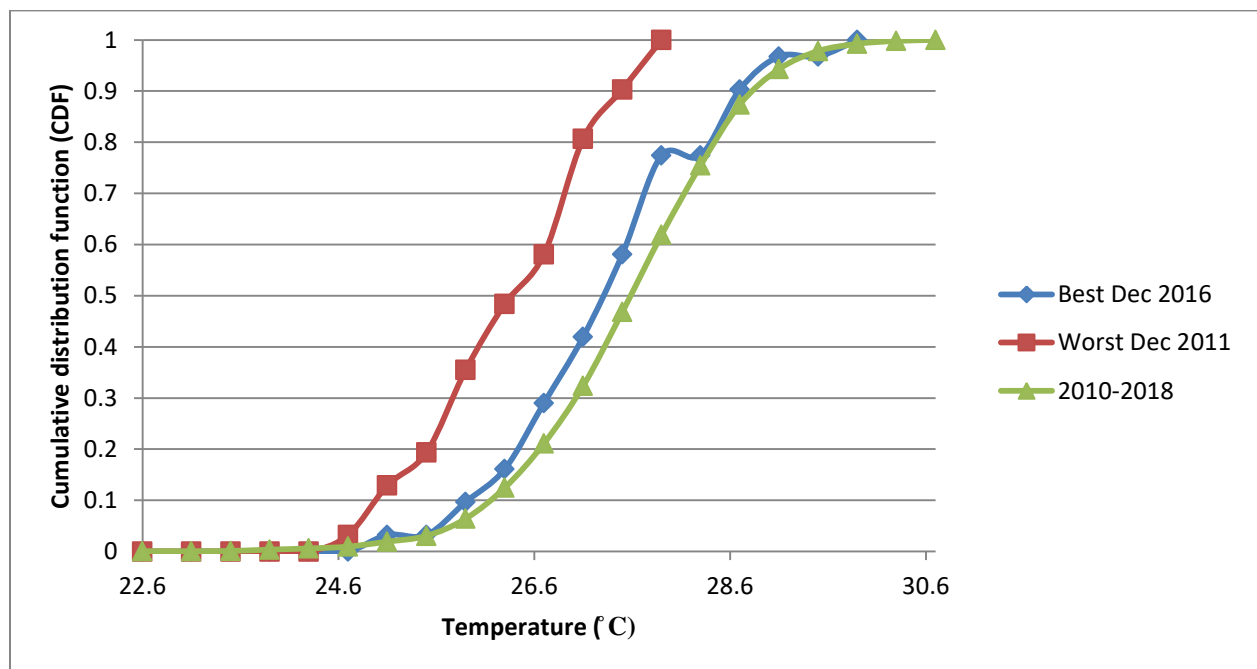

Relative humidity

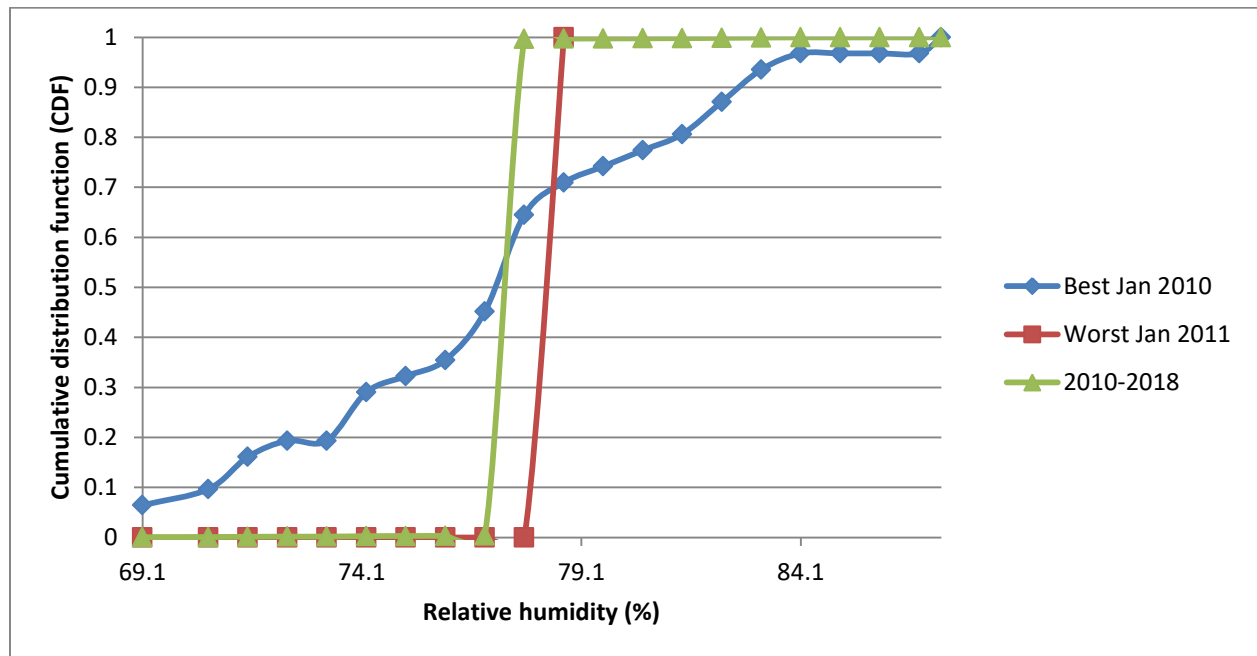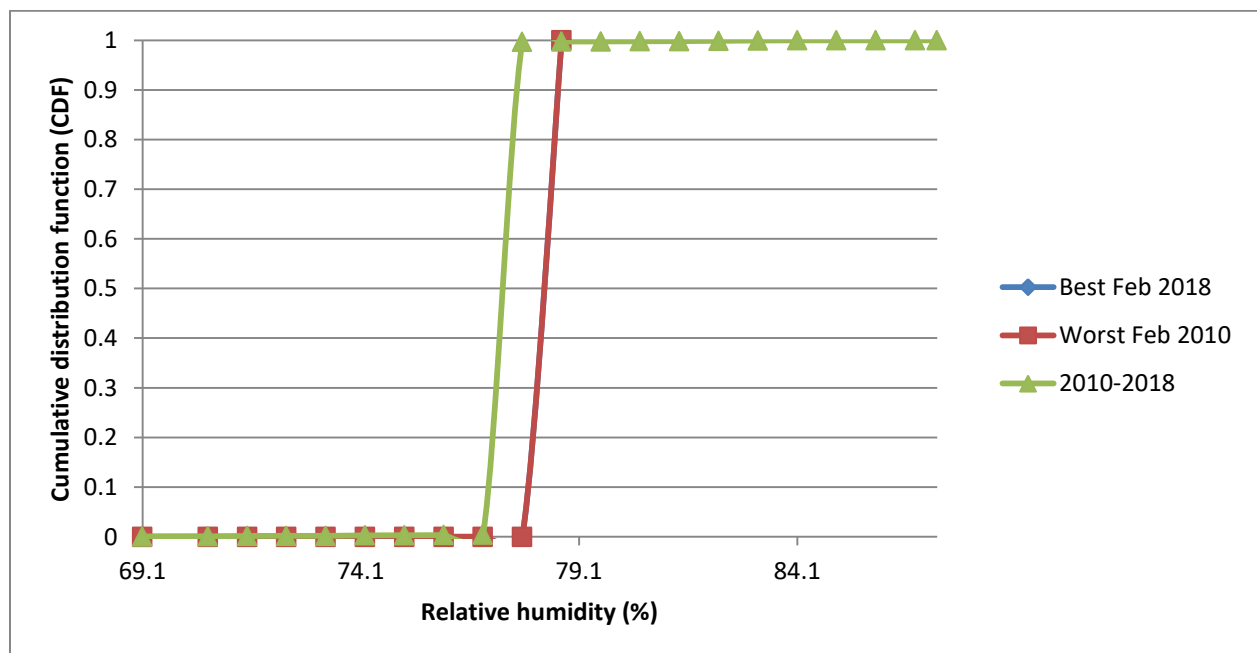

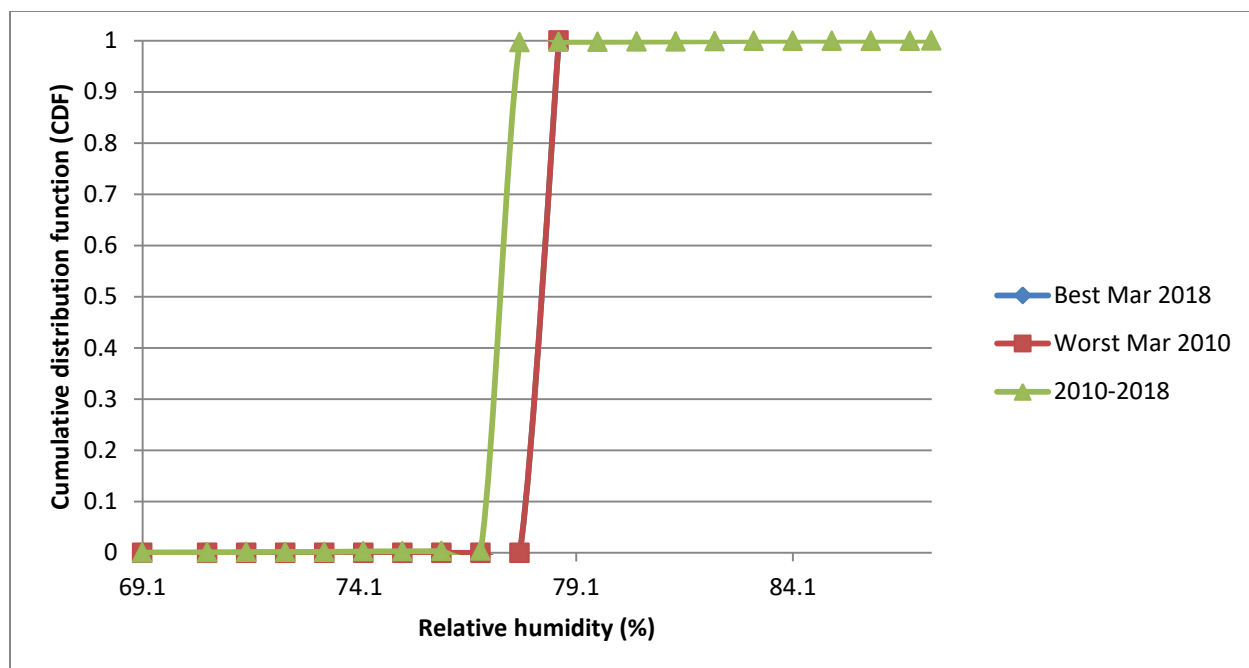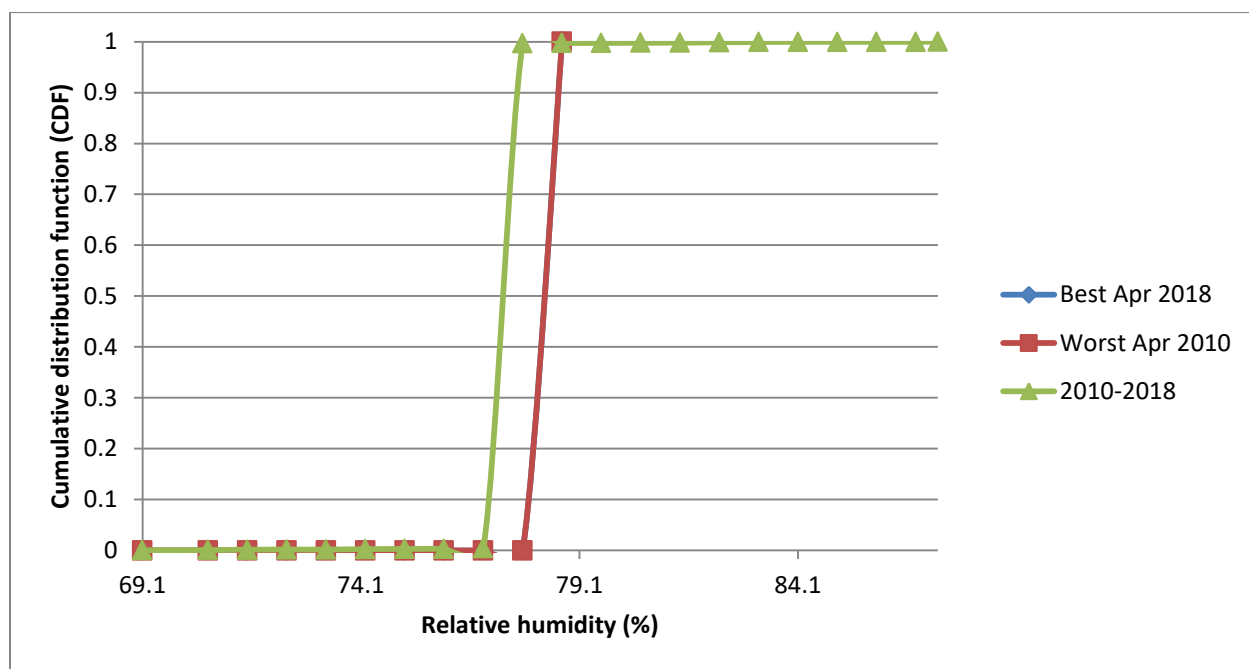

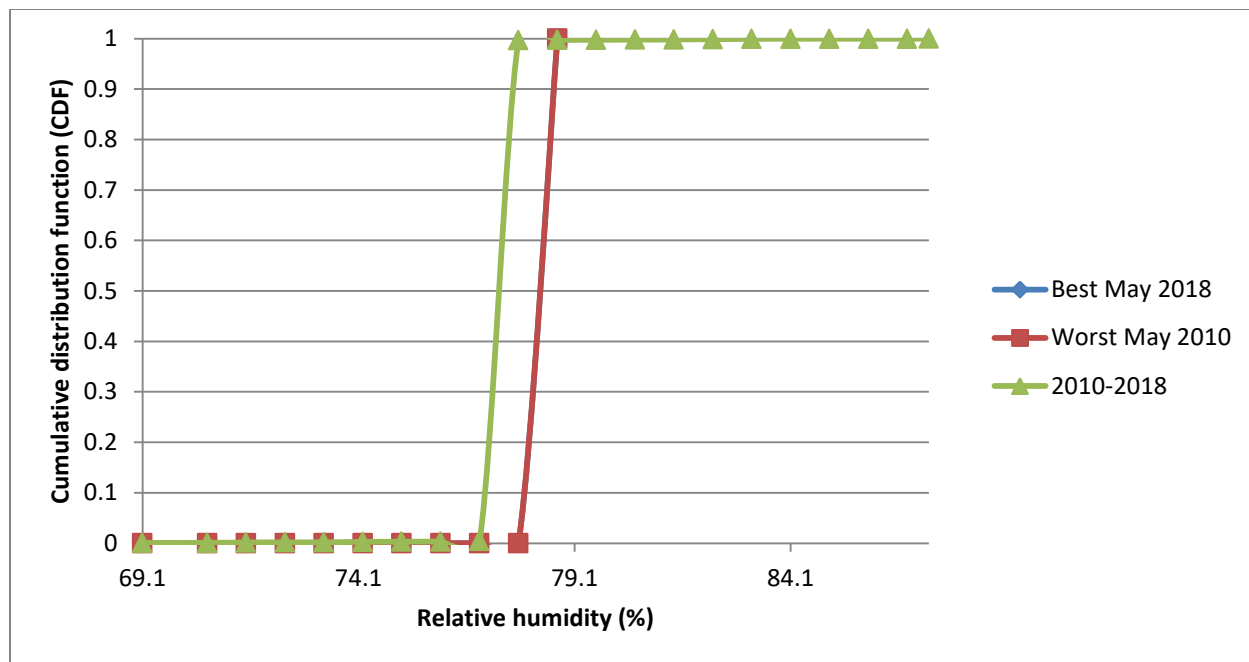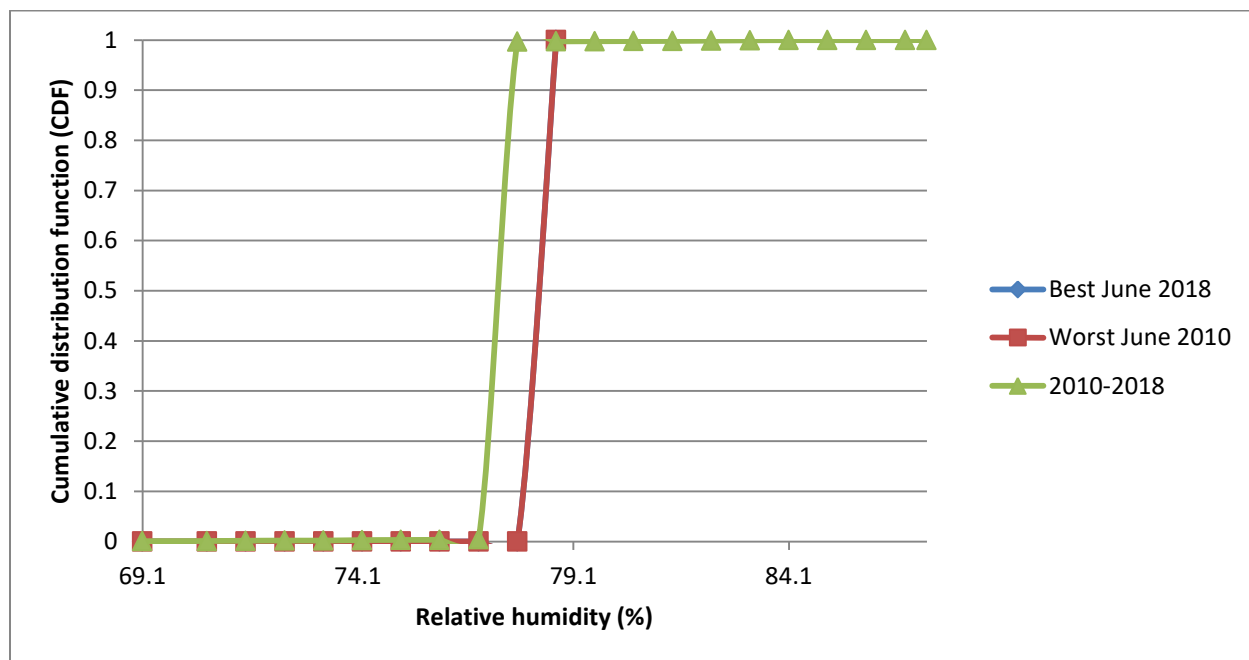

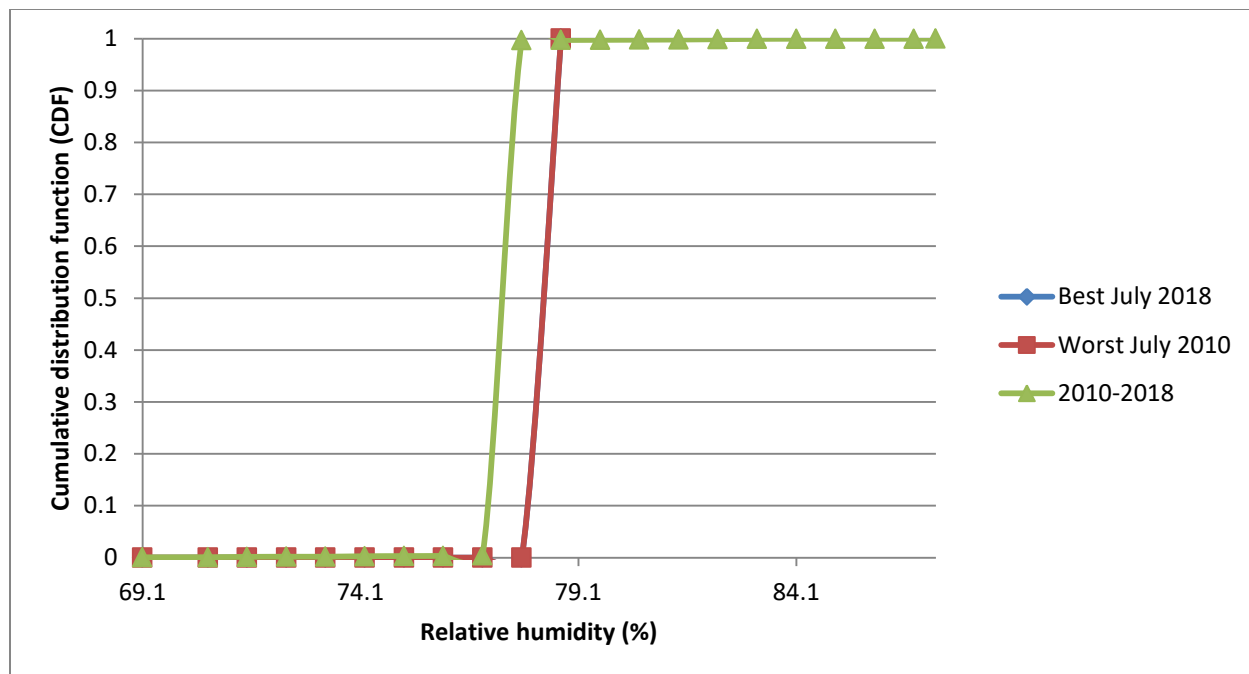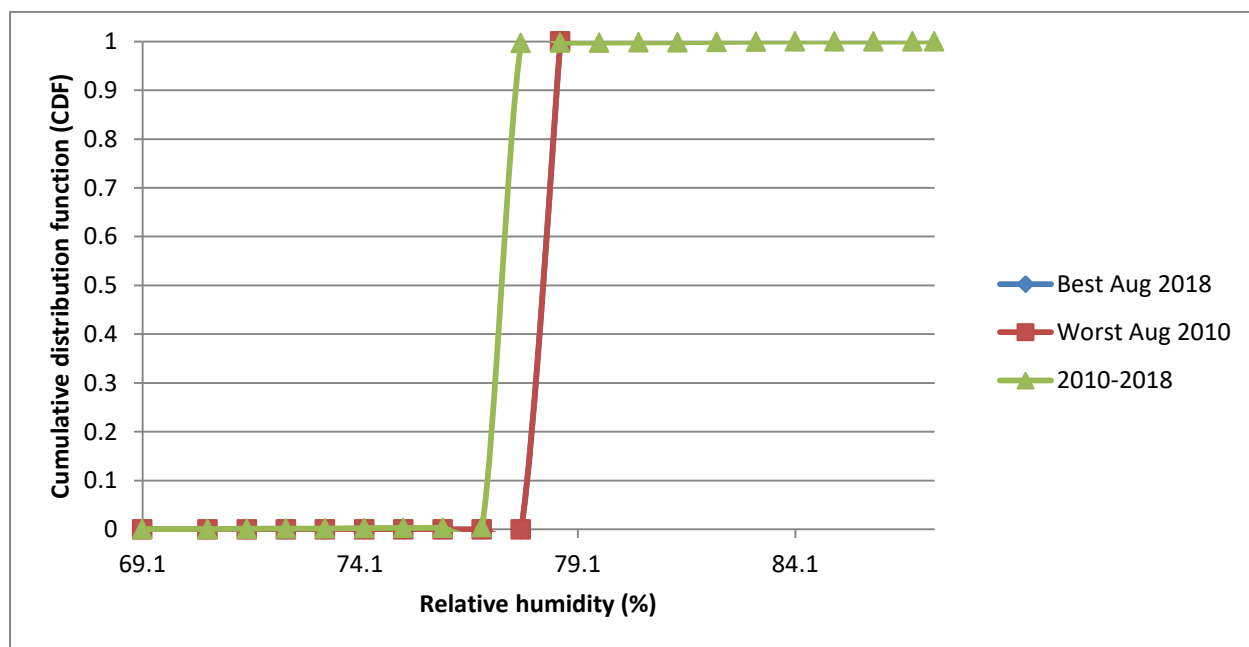

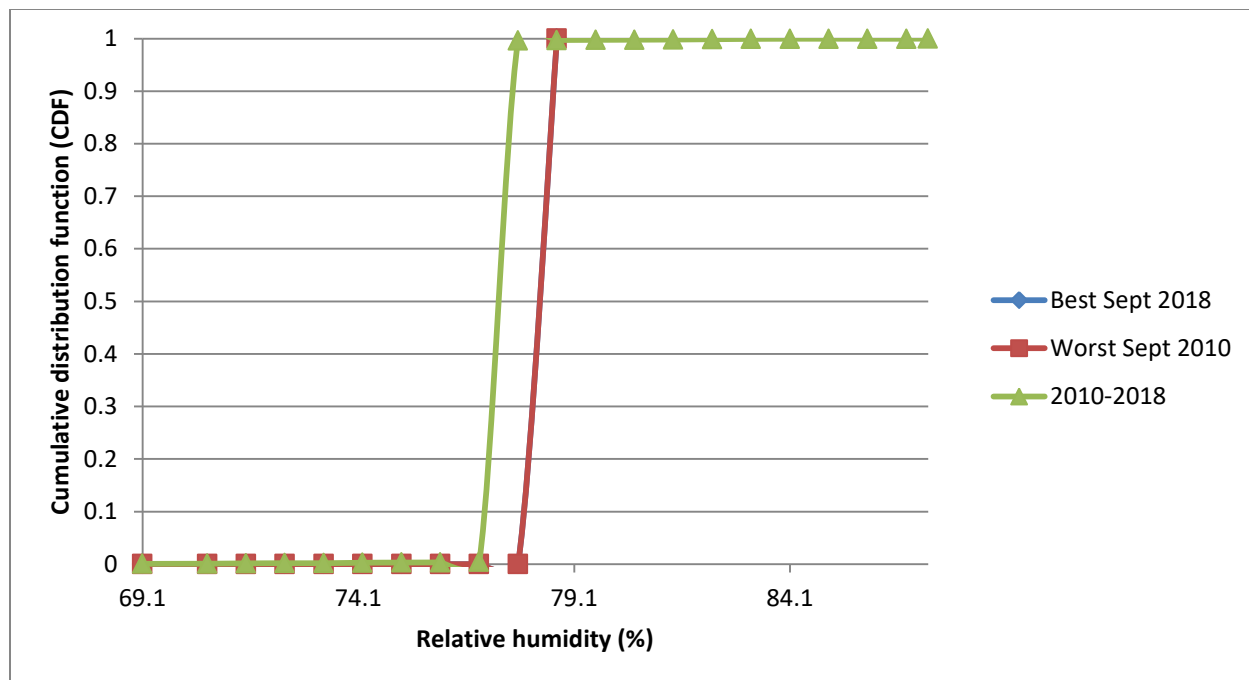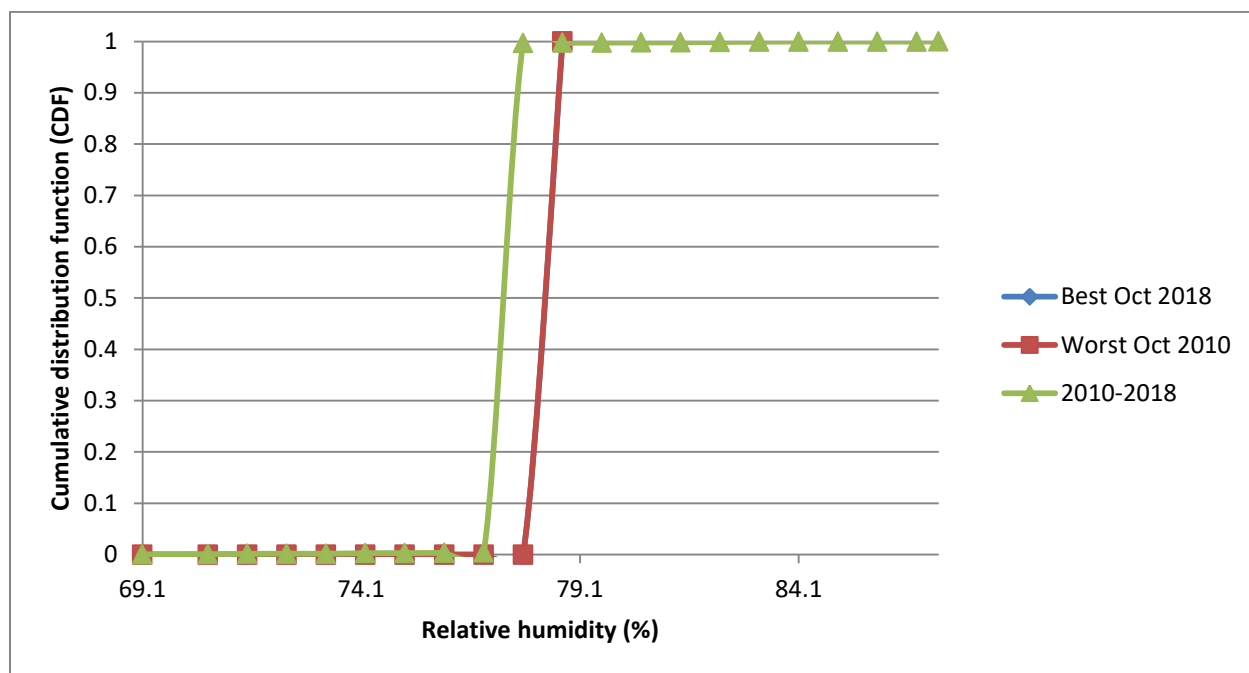

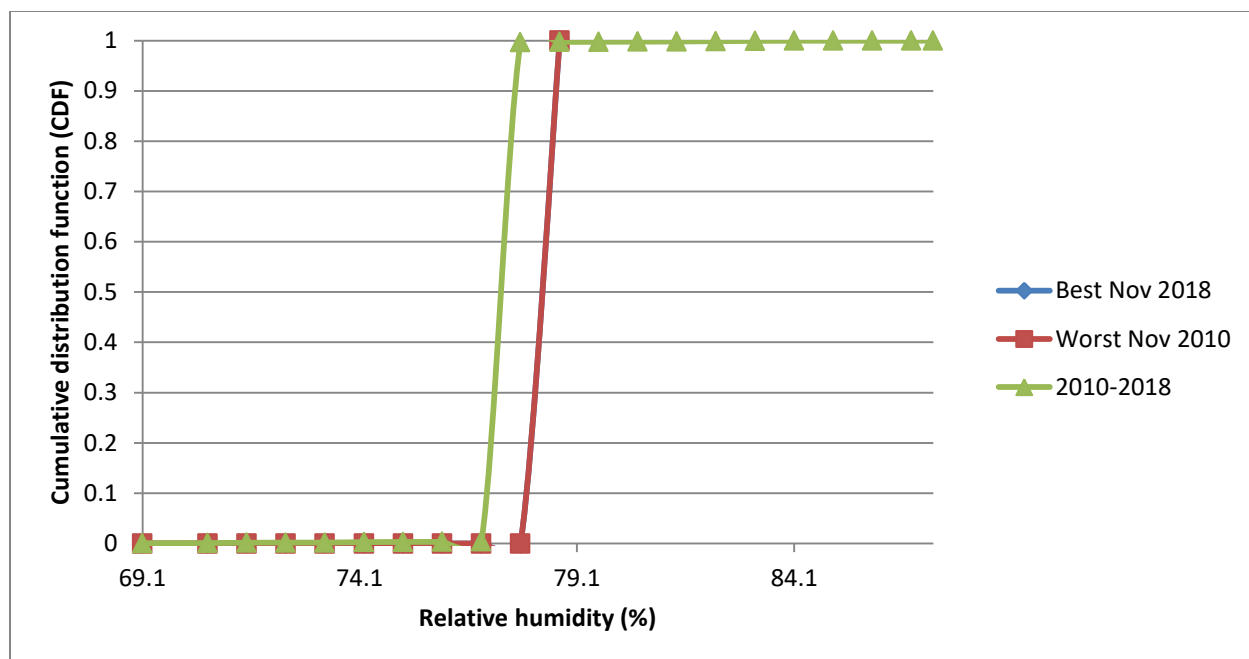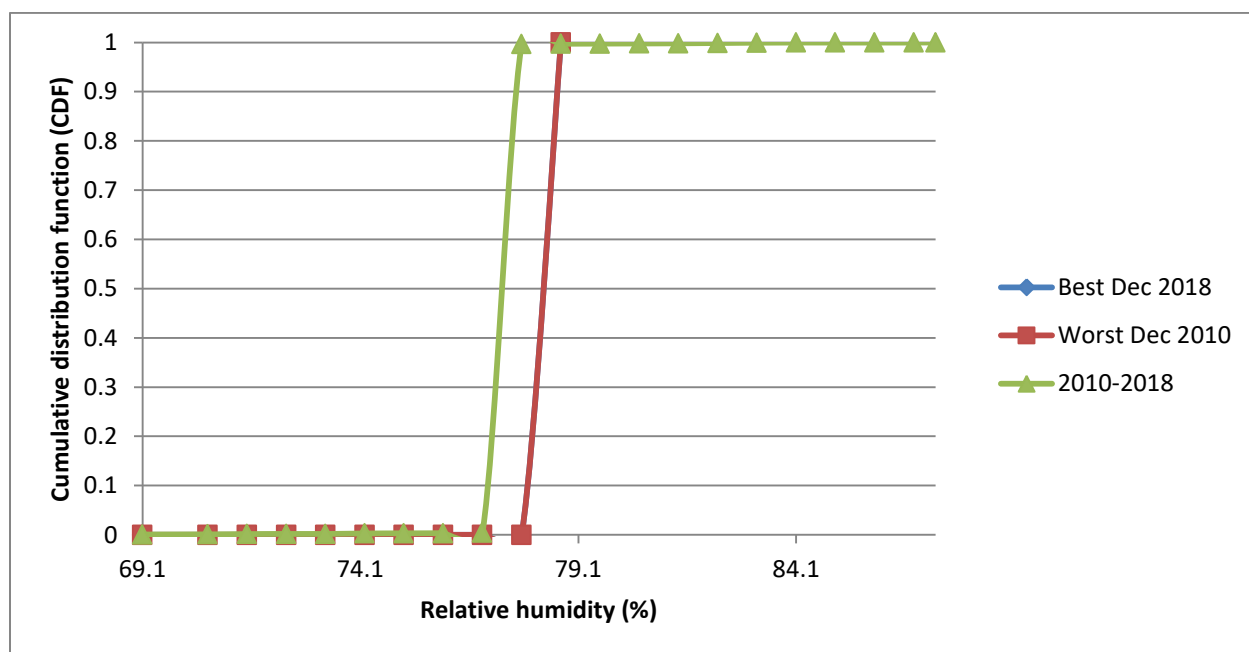

## Rainfall

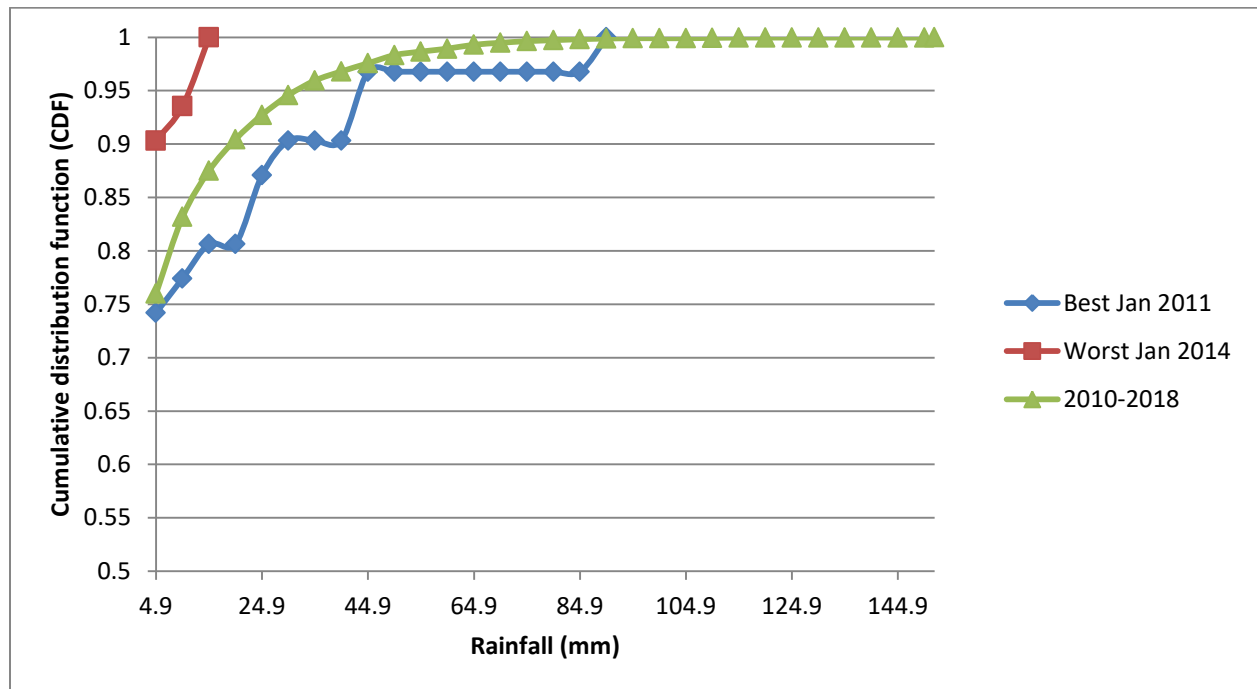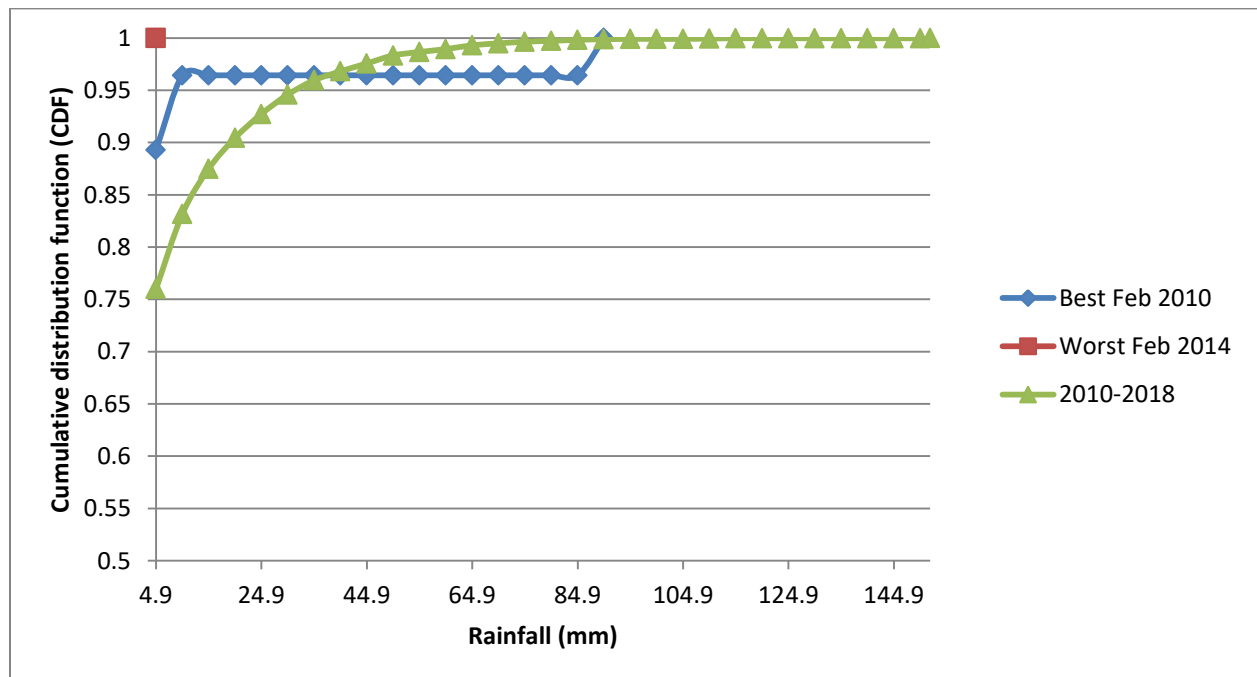

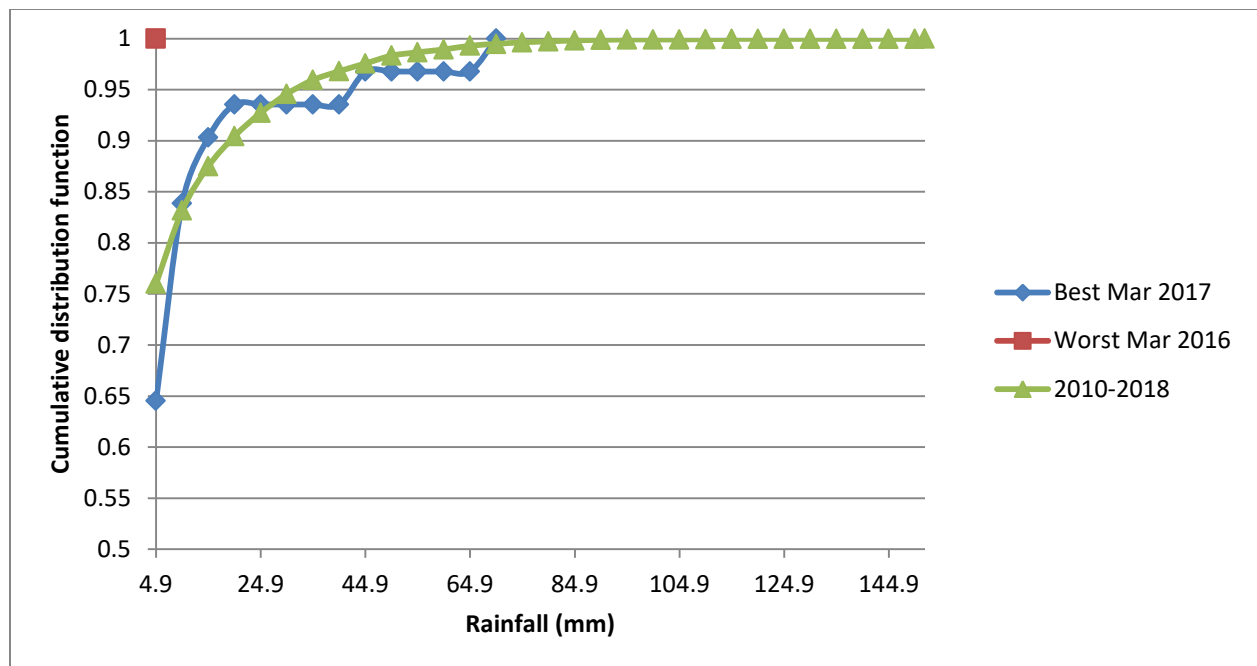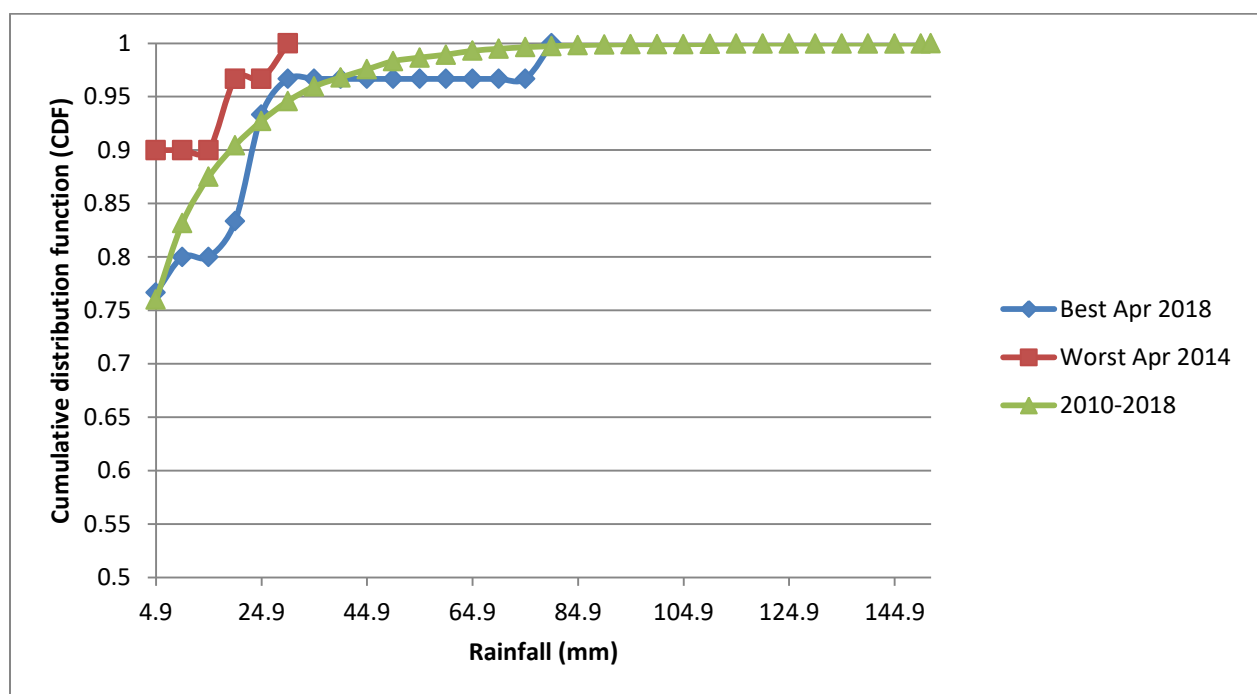

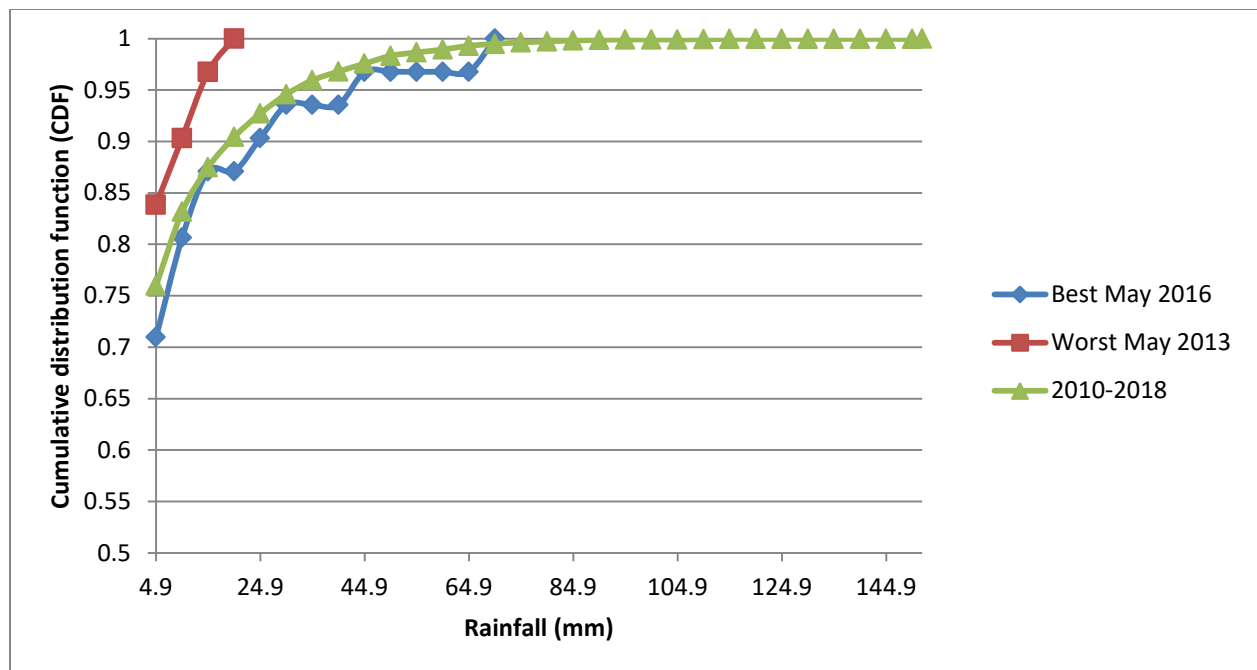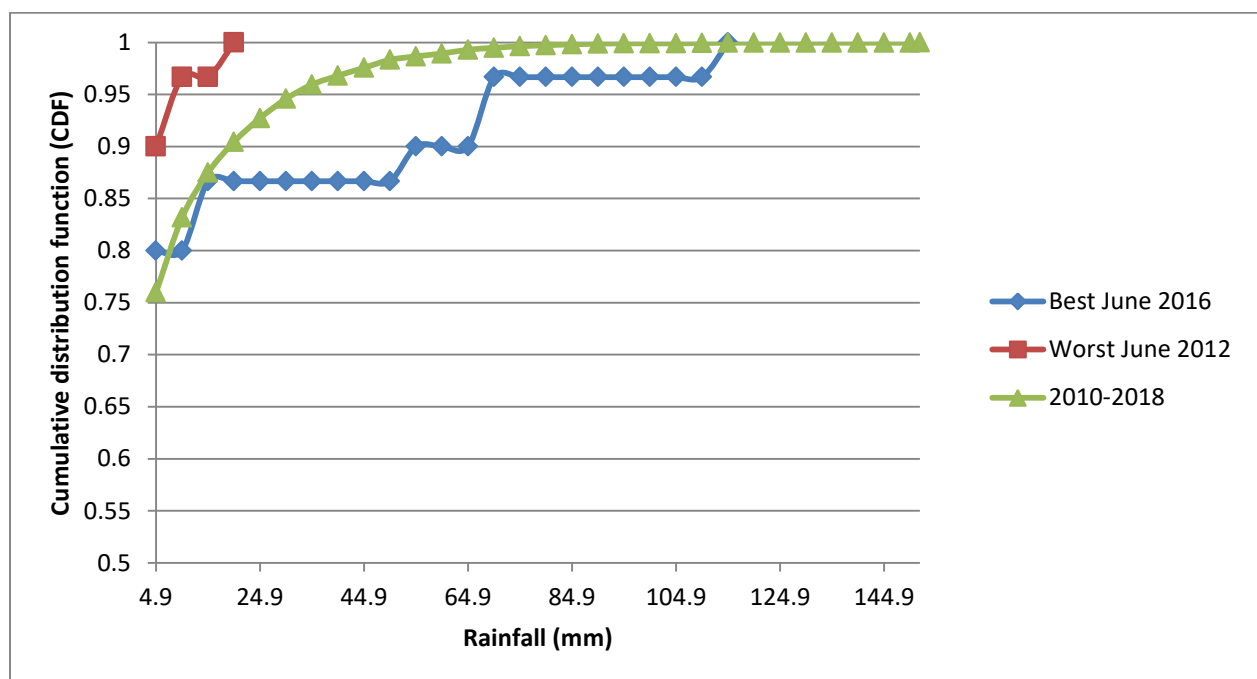

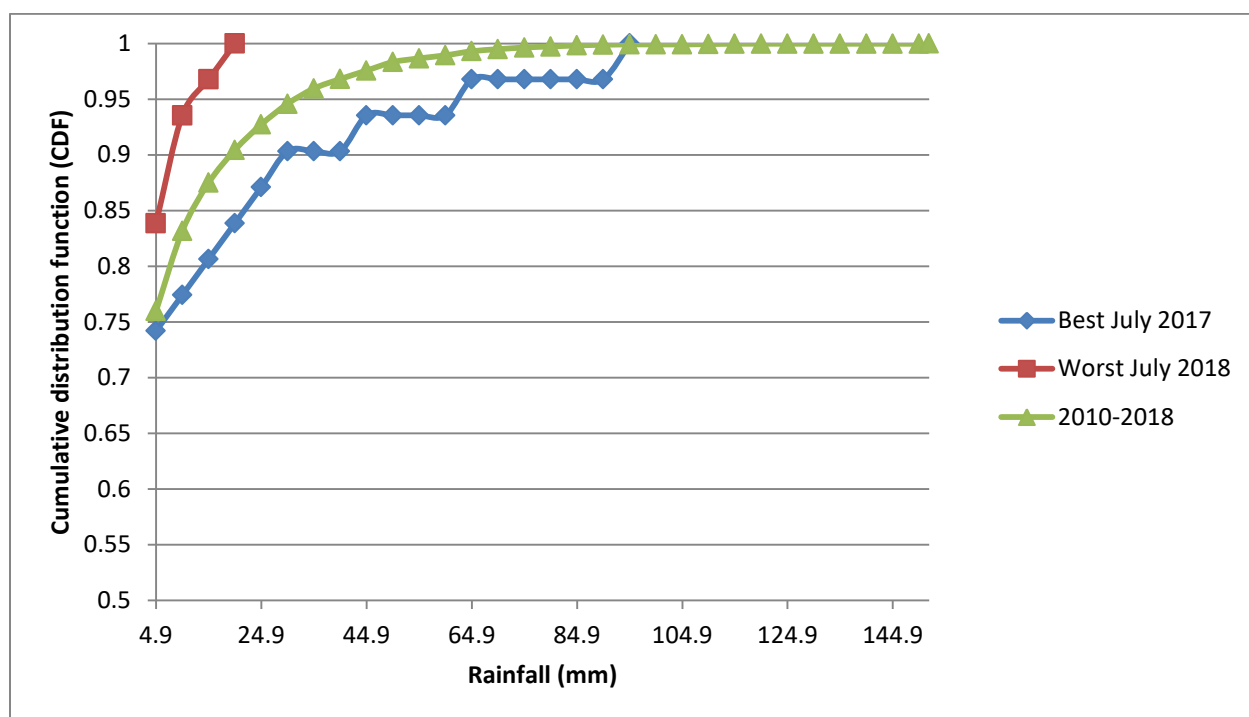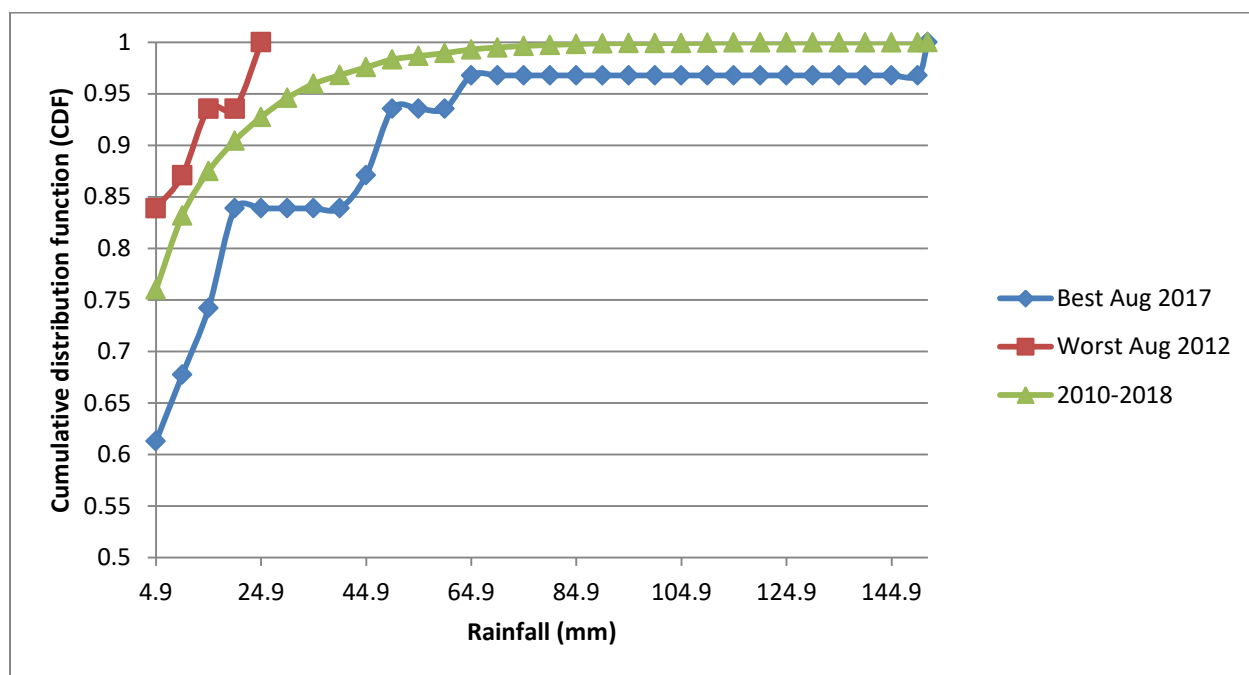

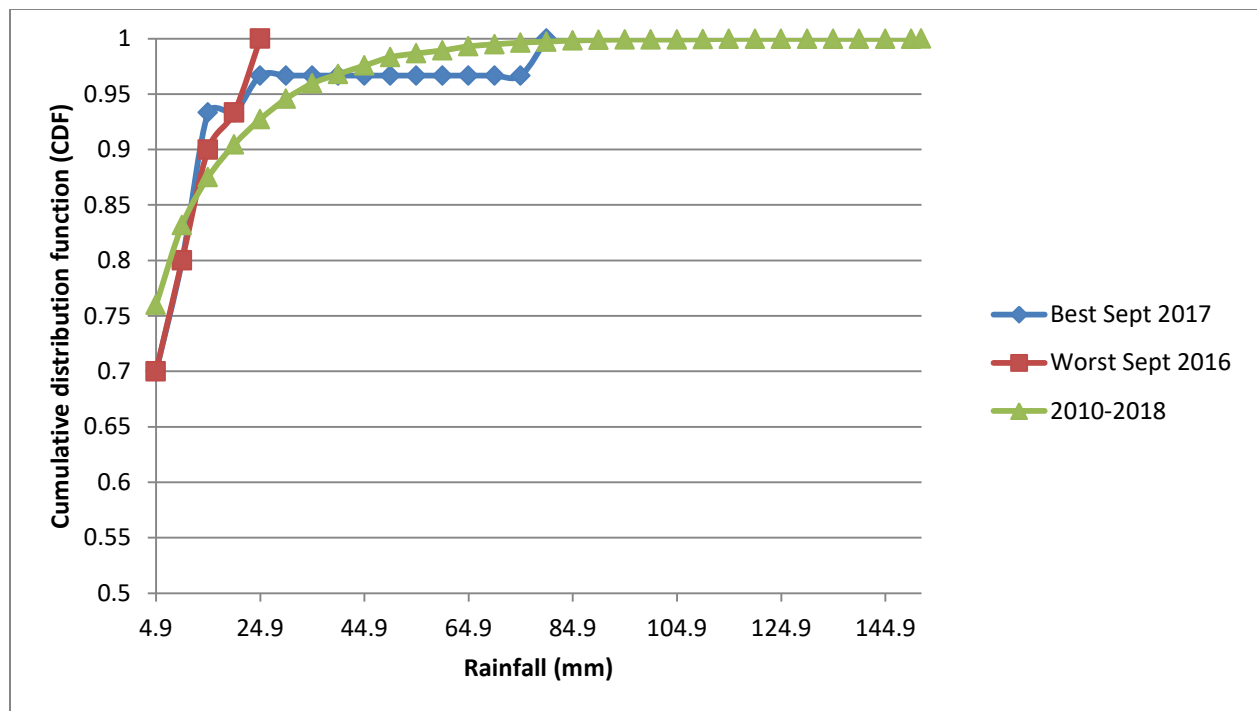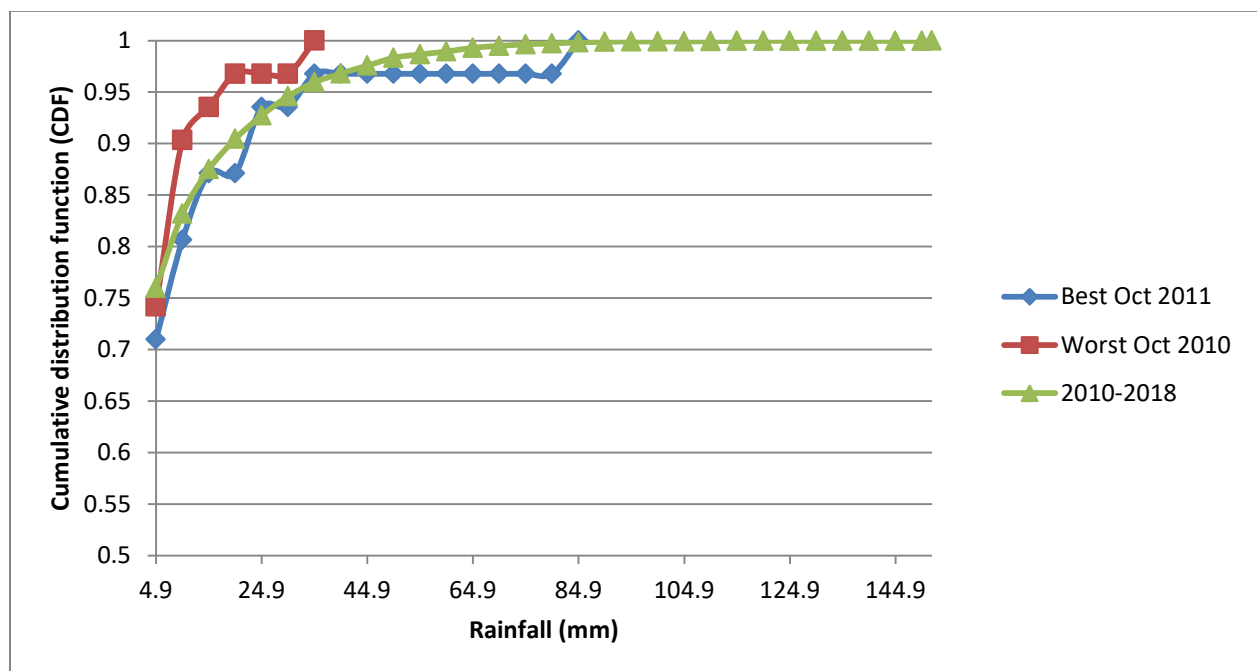

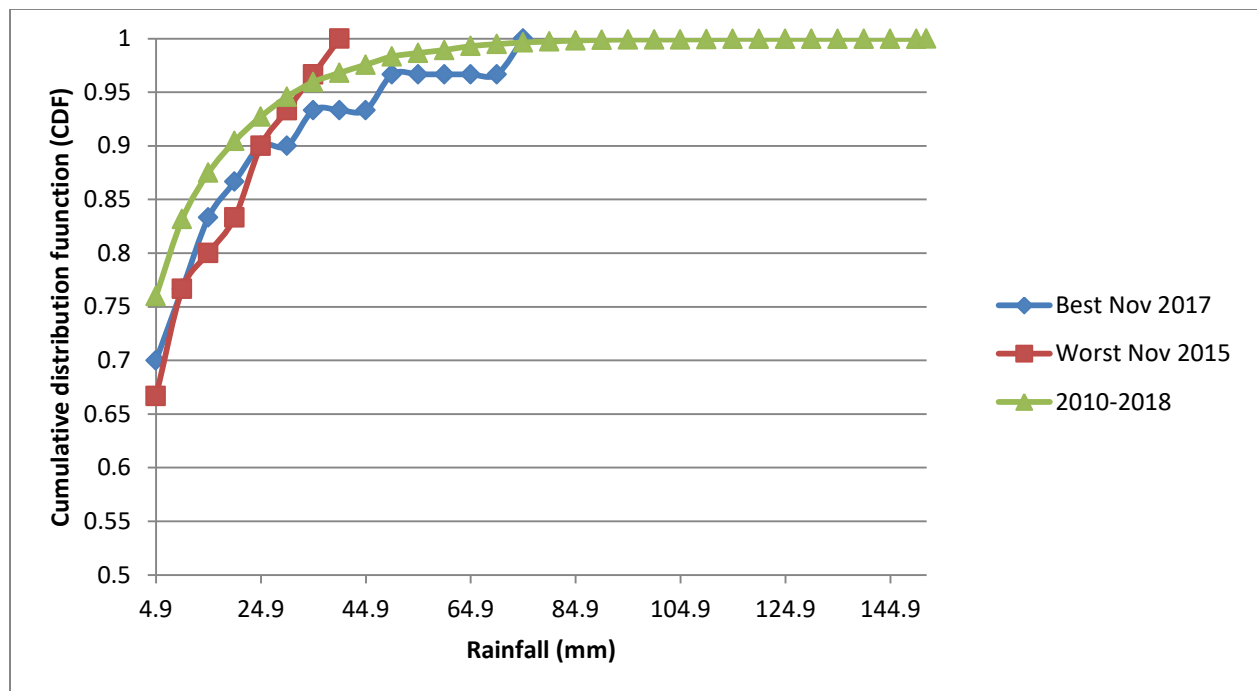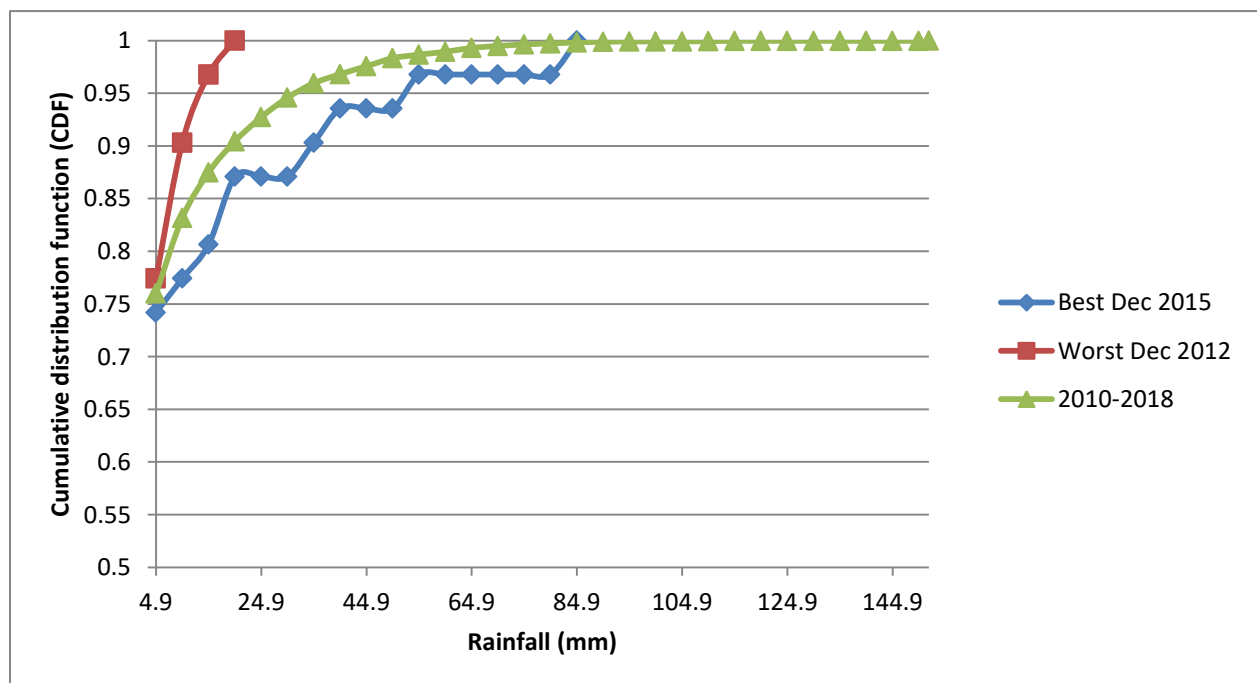

Supplement: Supplementary file 1 [file mmc1.zip › graph cdf best and worst weather data.pdf]

### Compilation 3: Graph CDF Compile

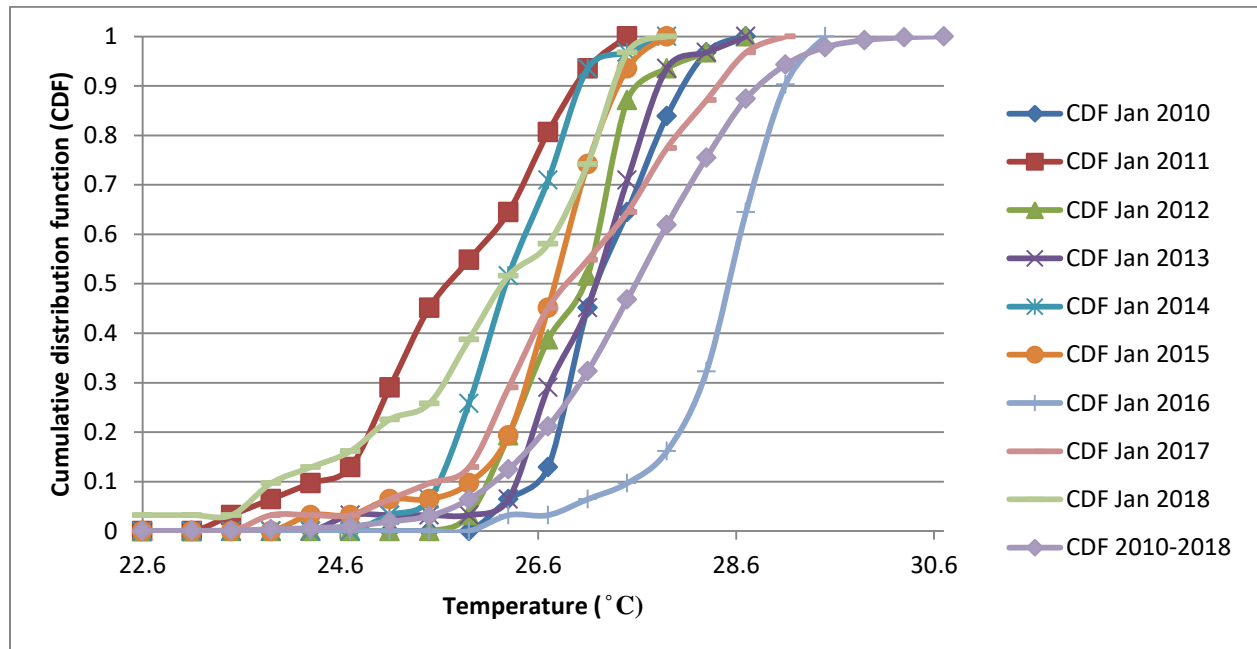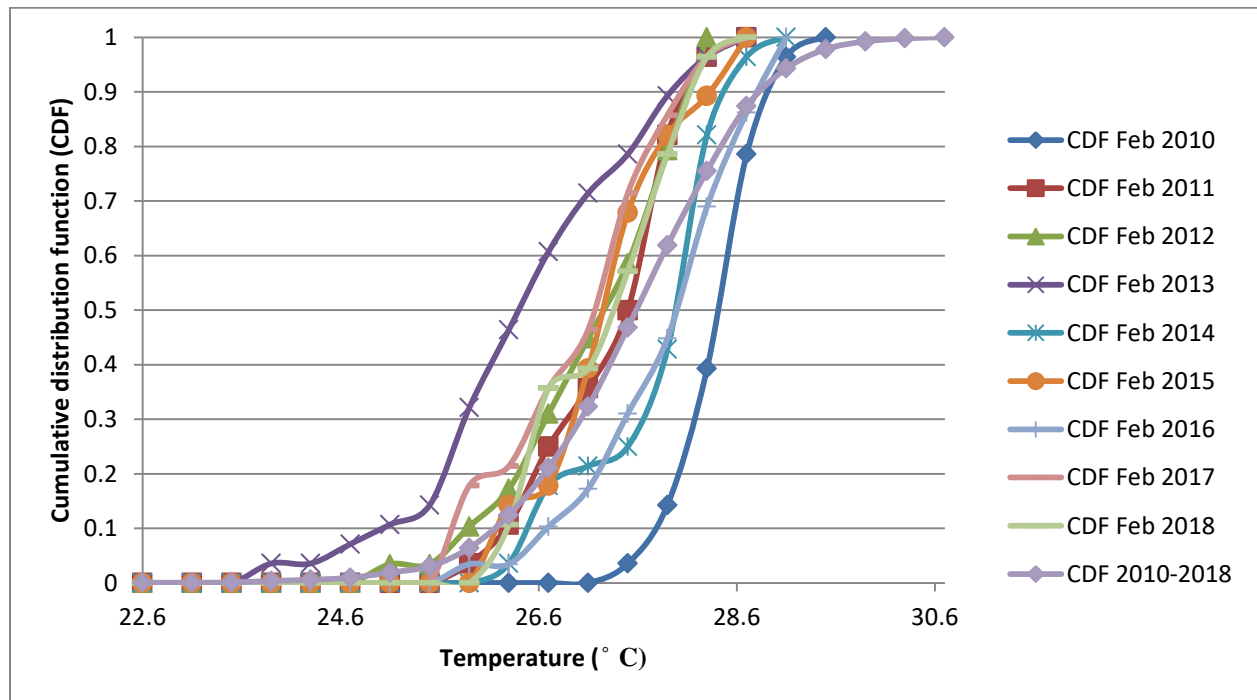

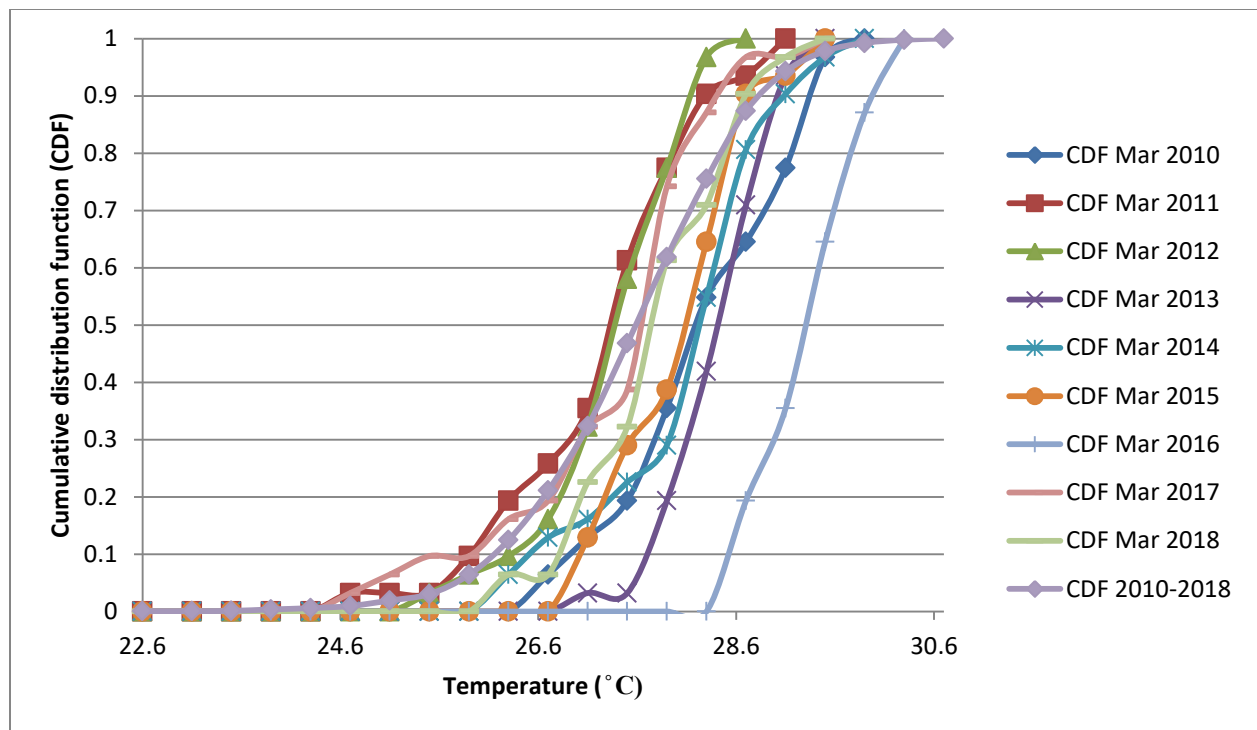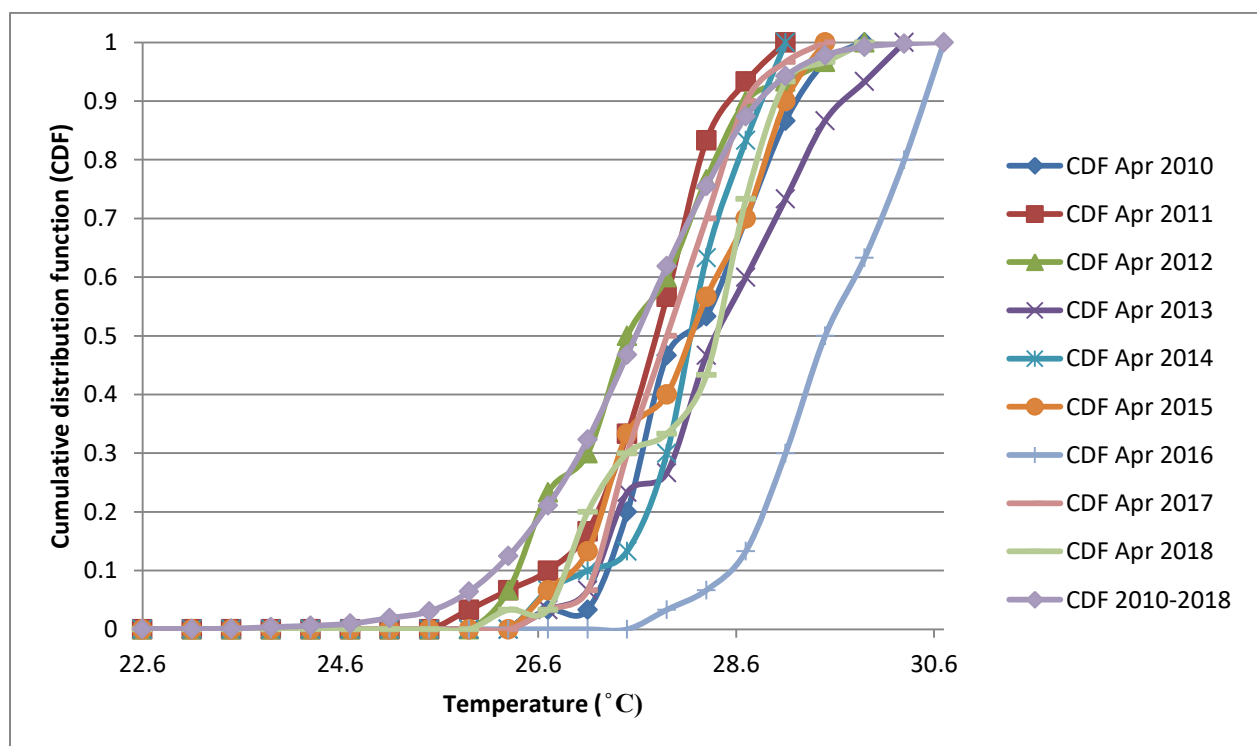

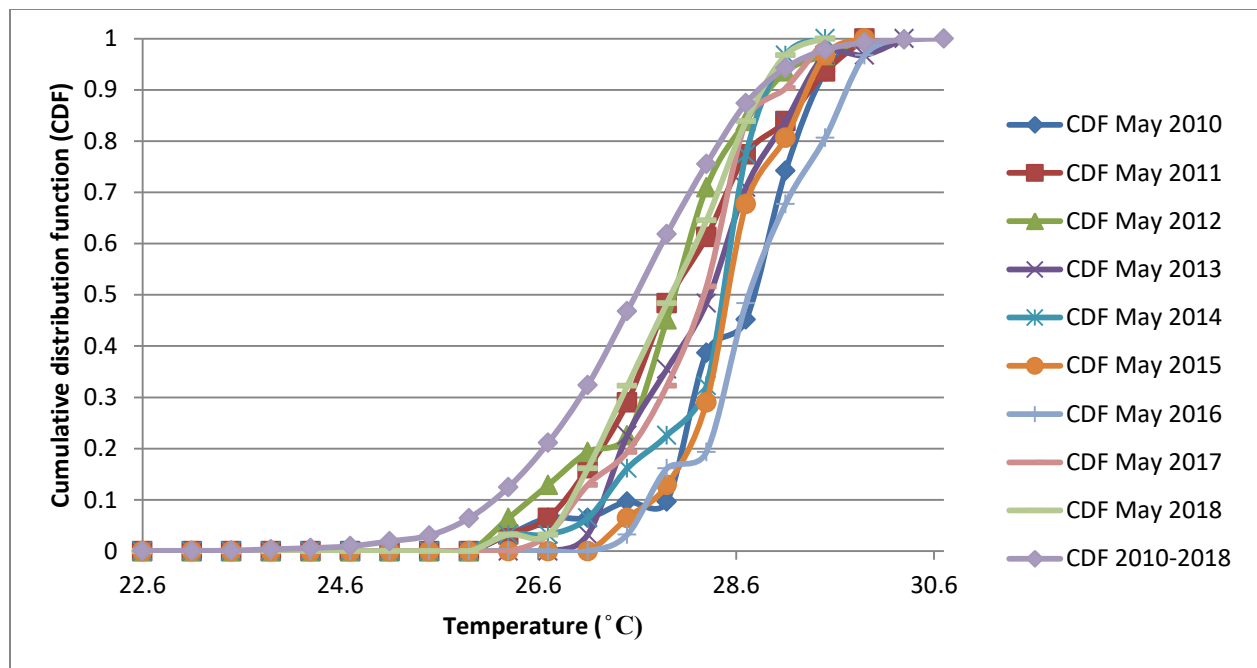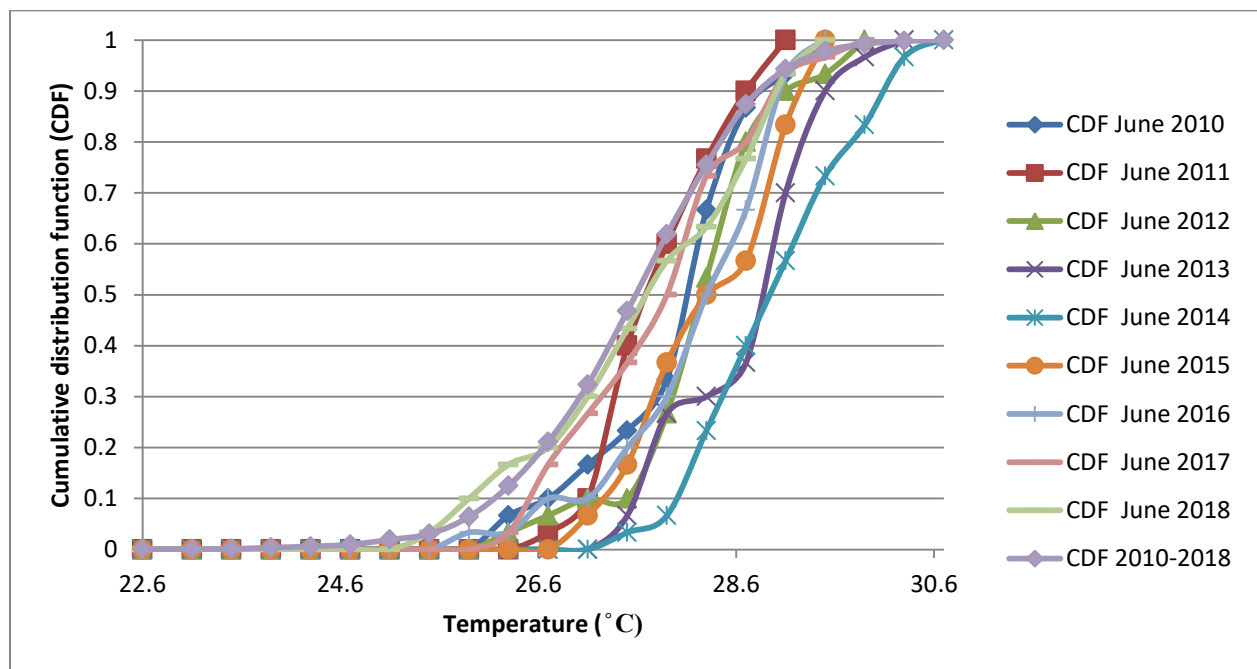



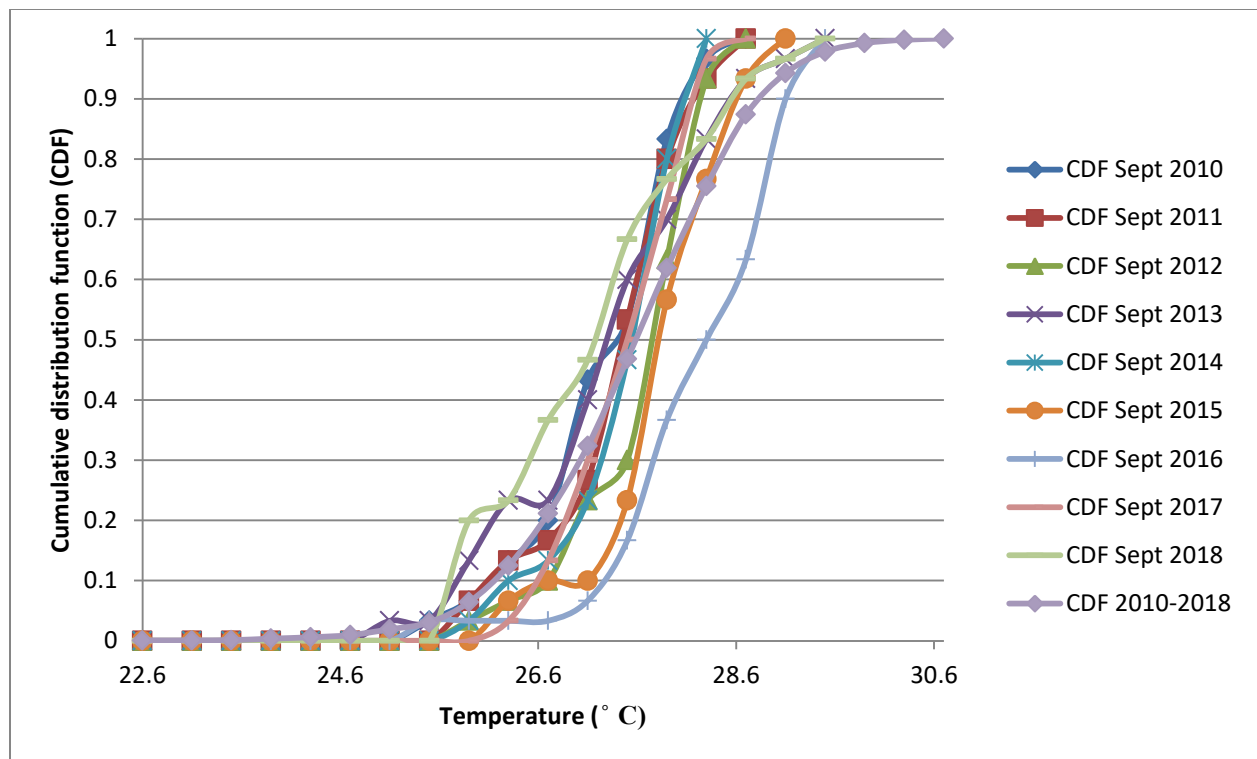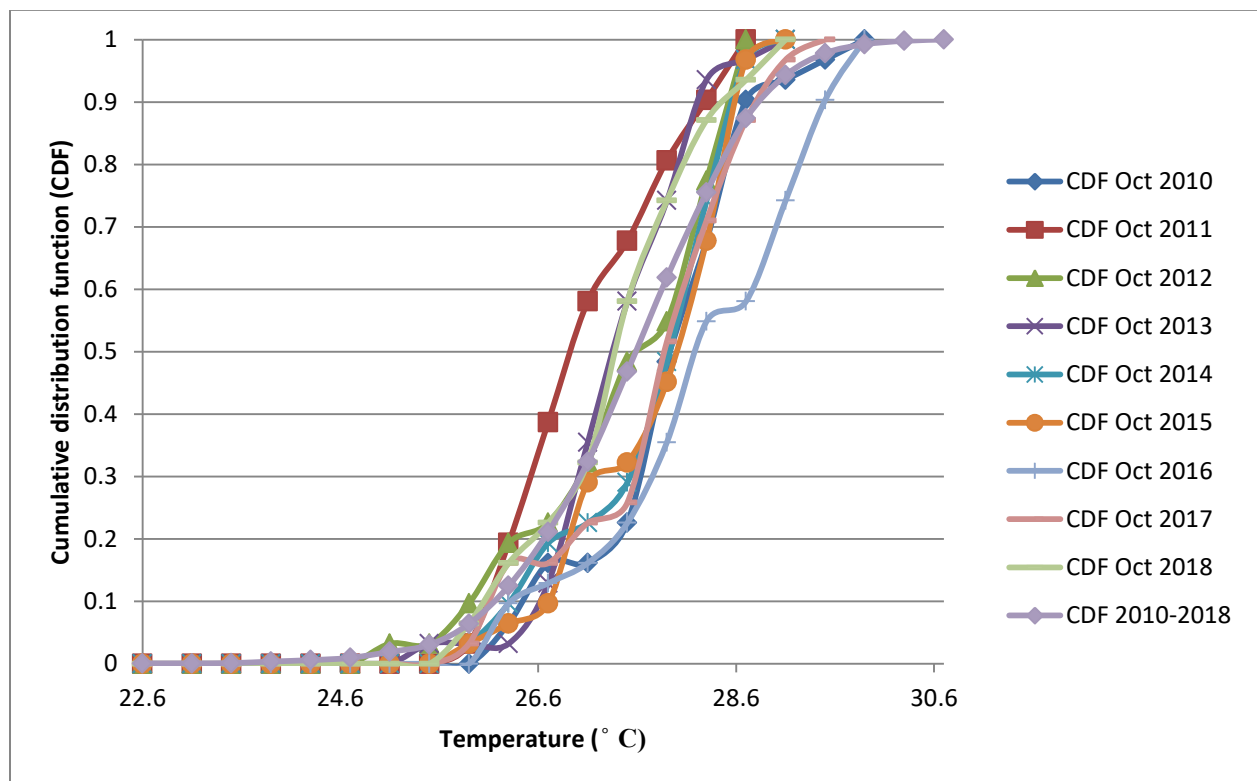





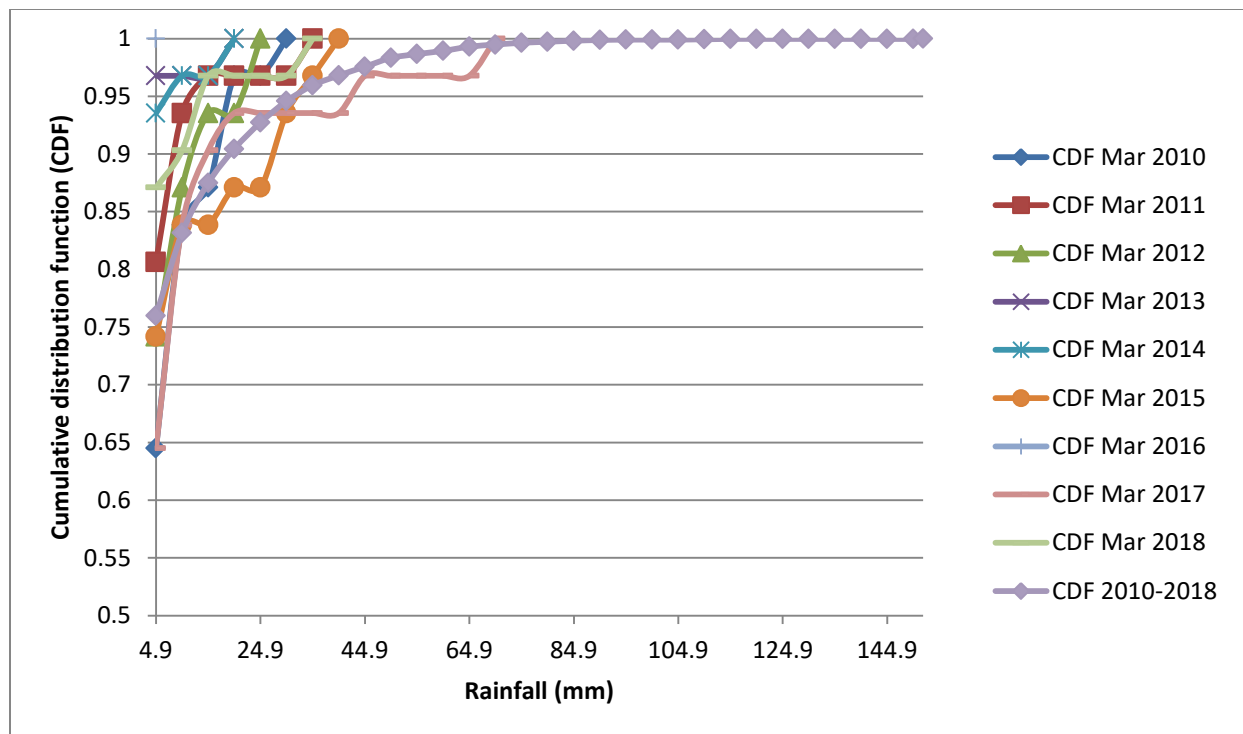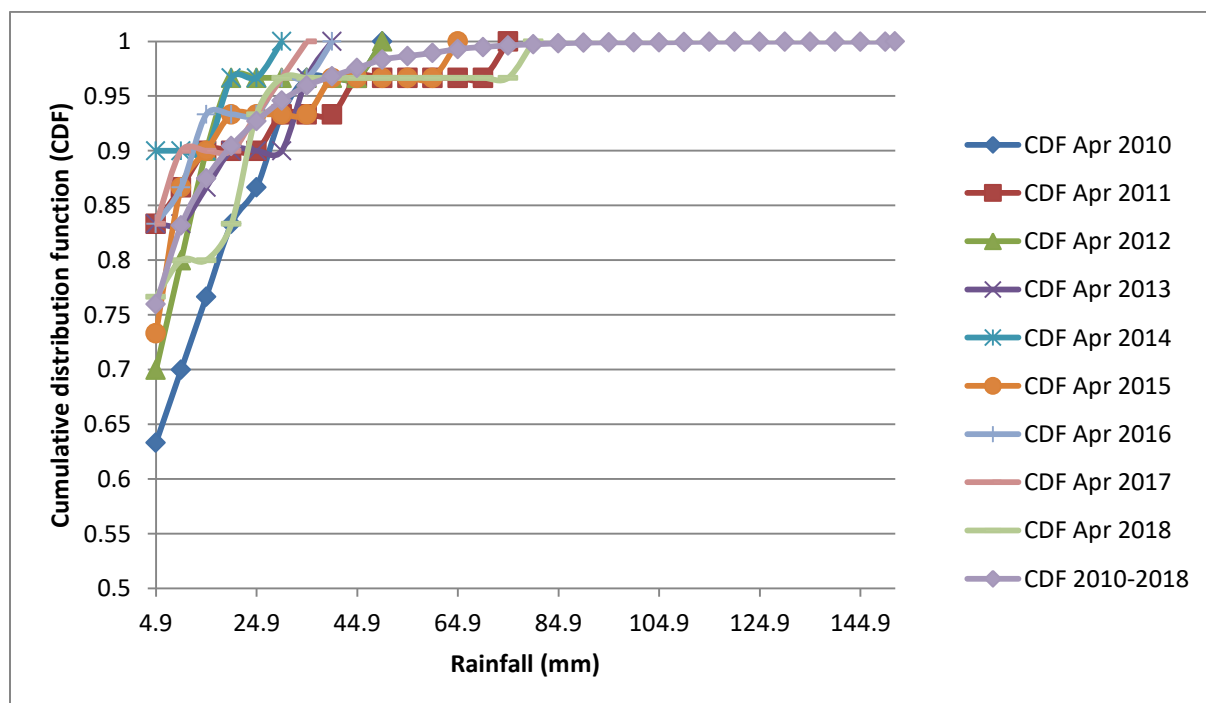

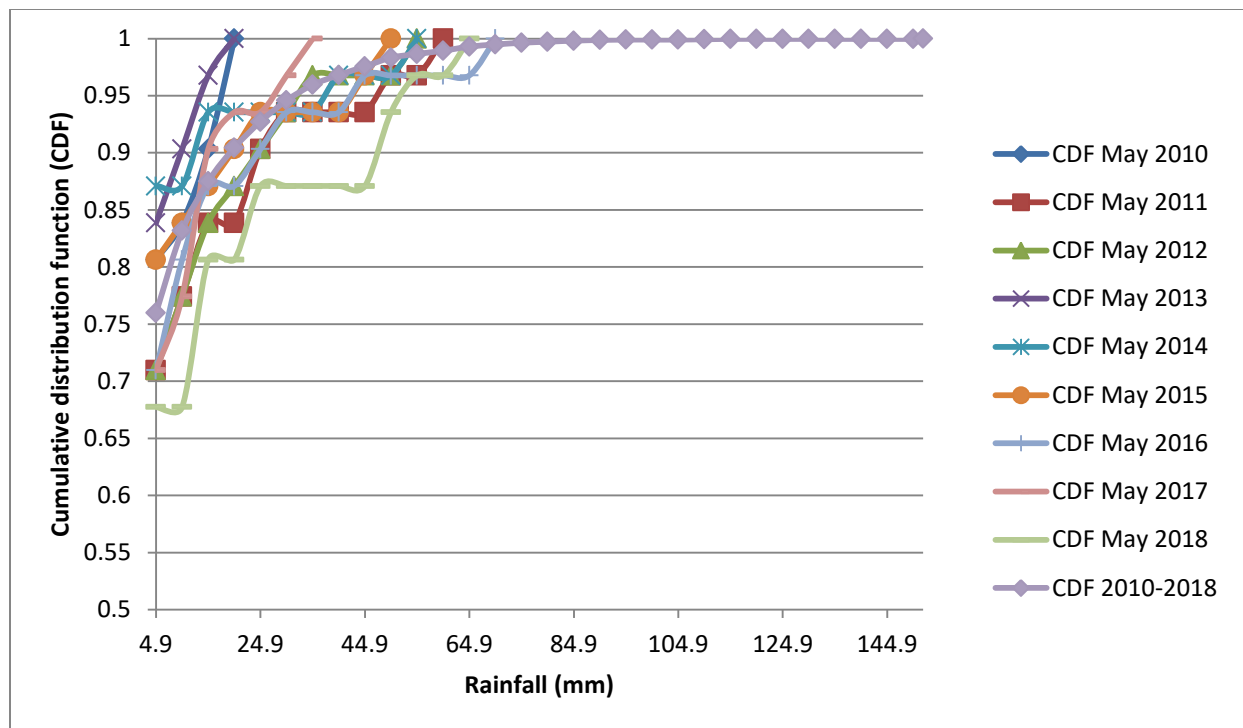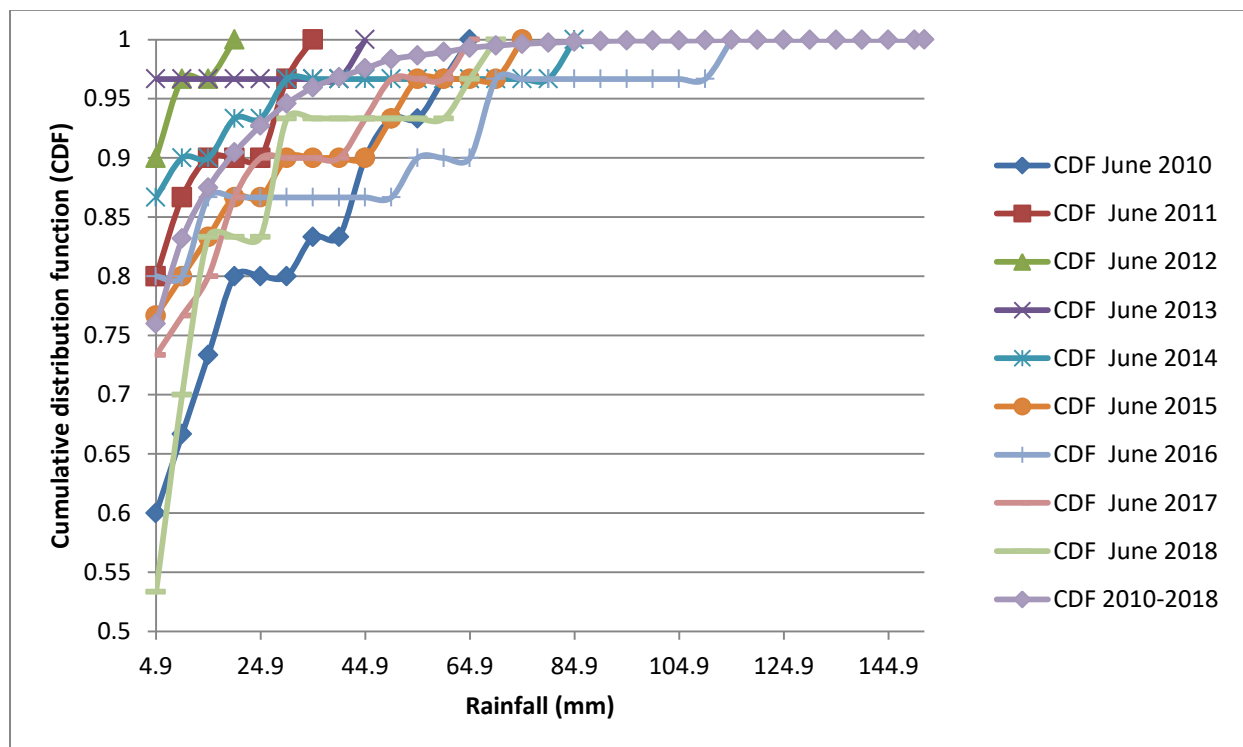



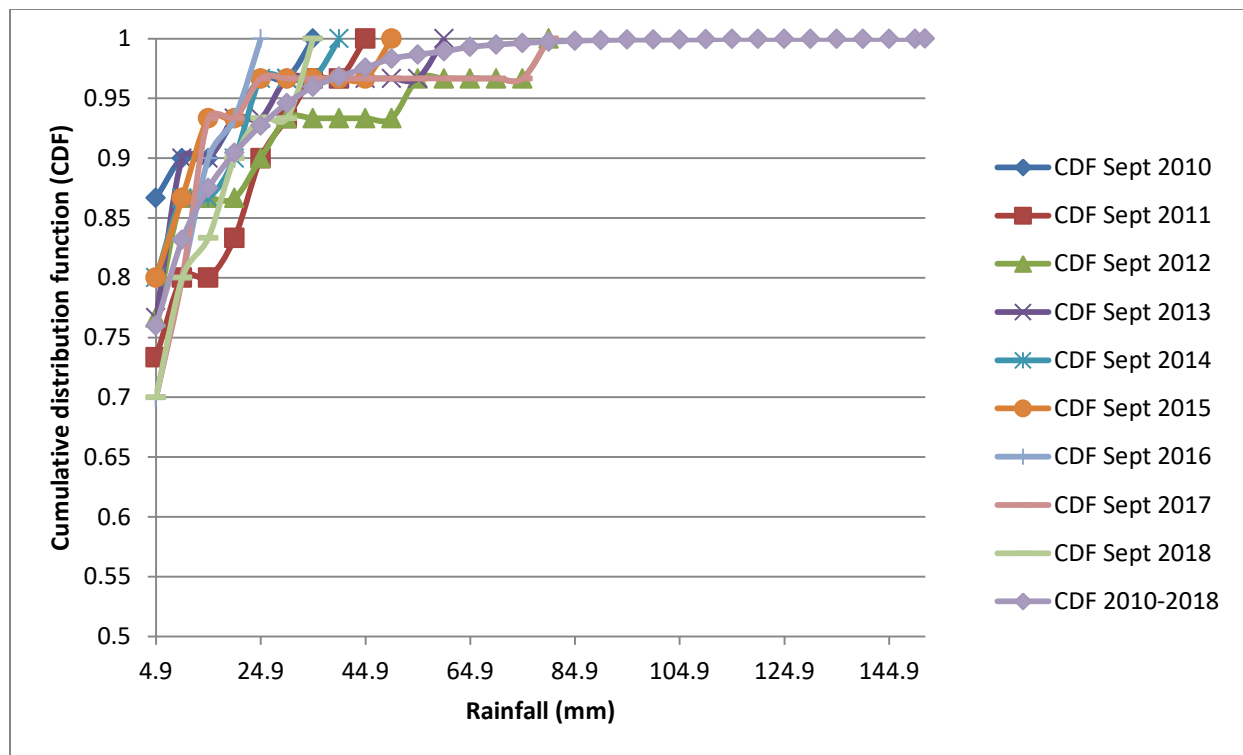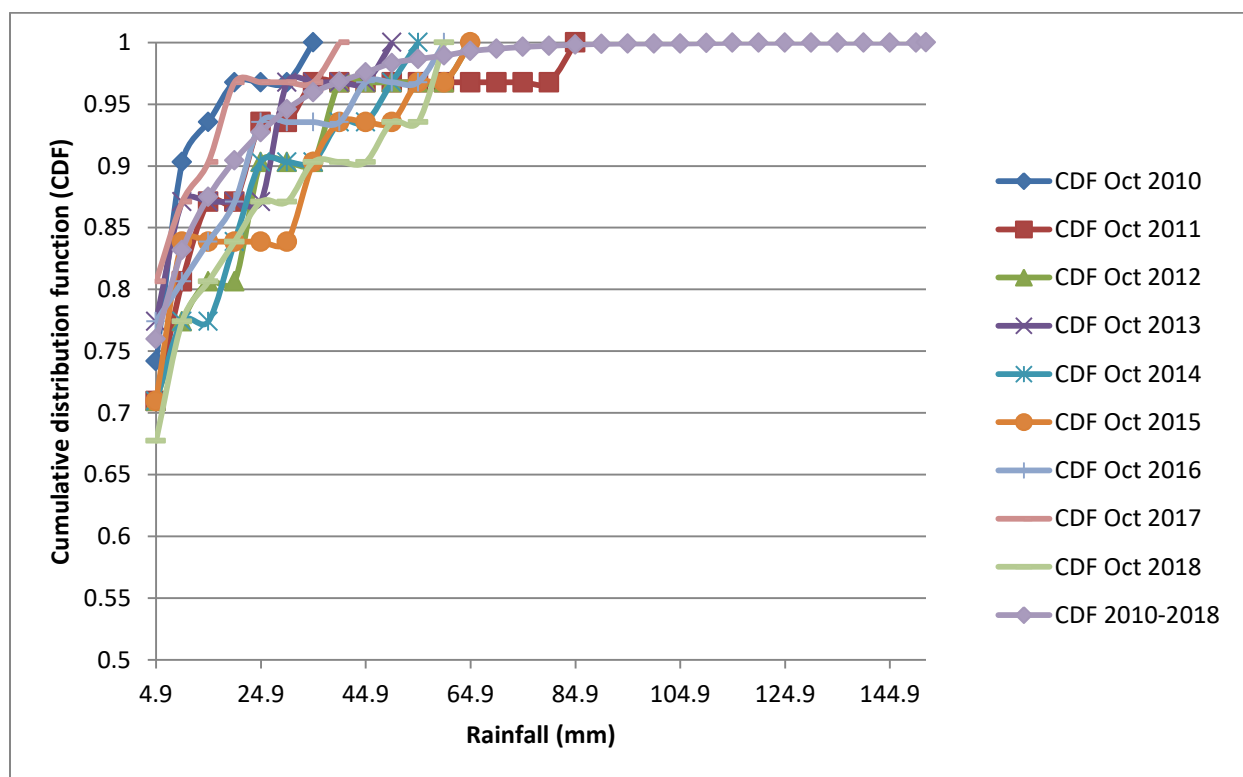

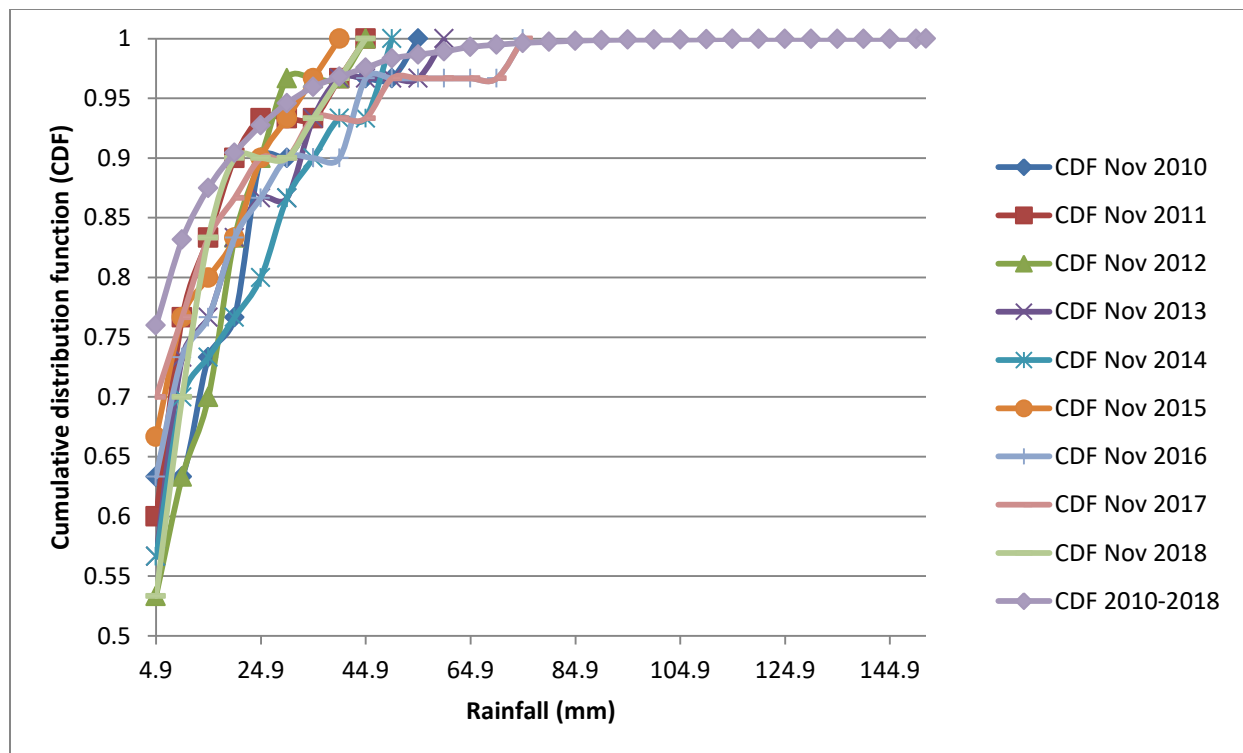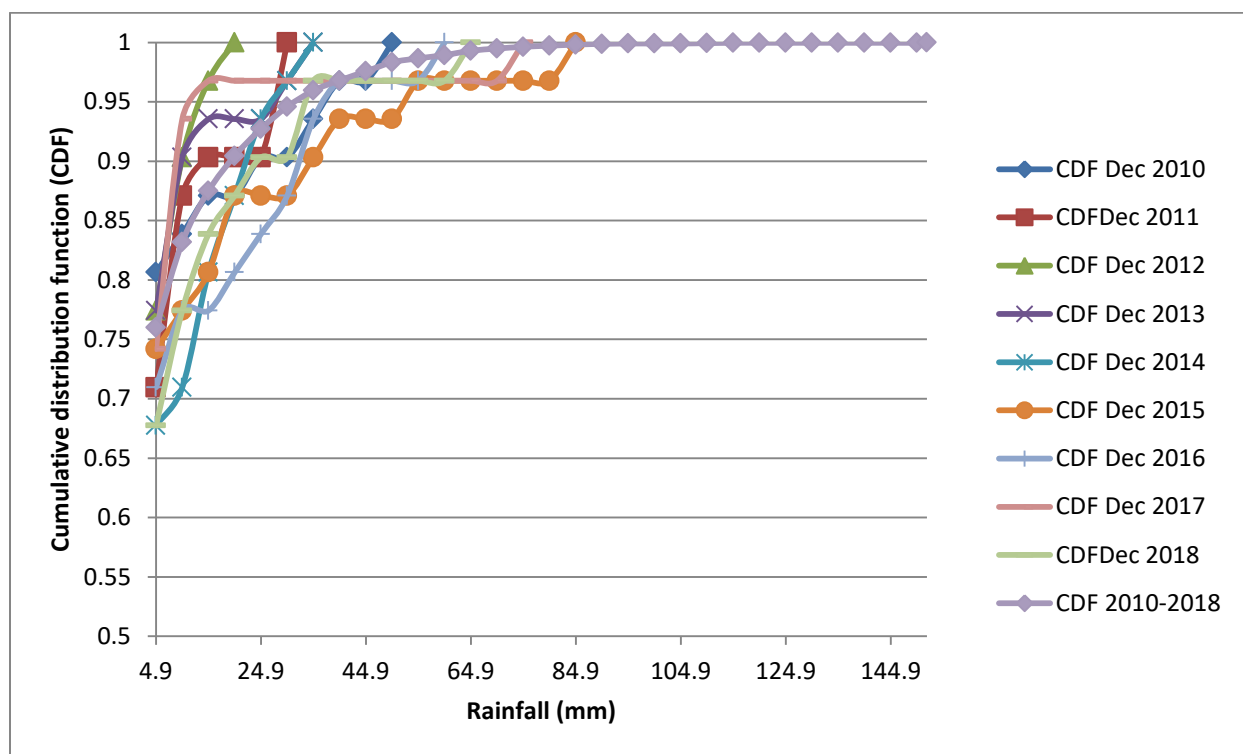

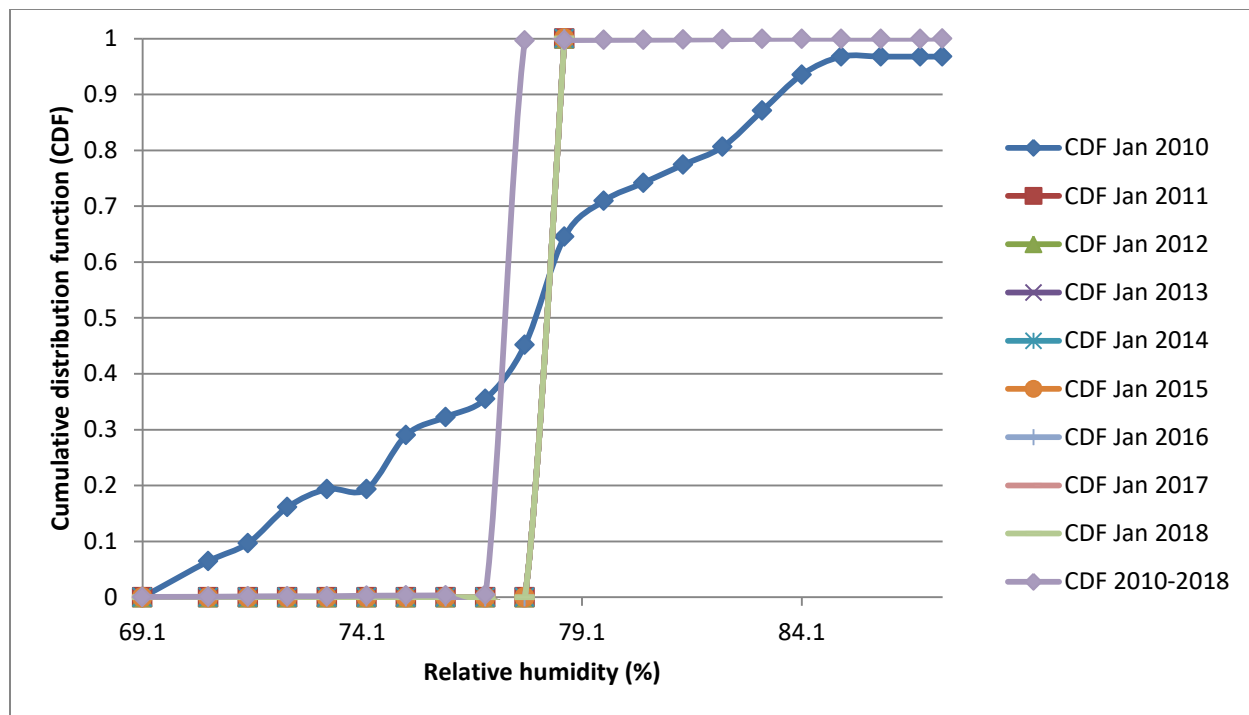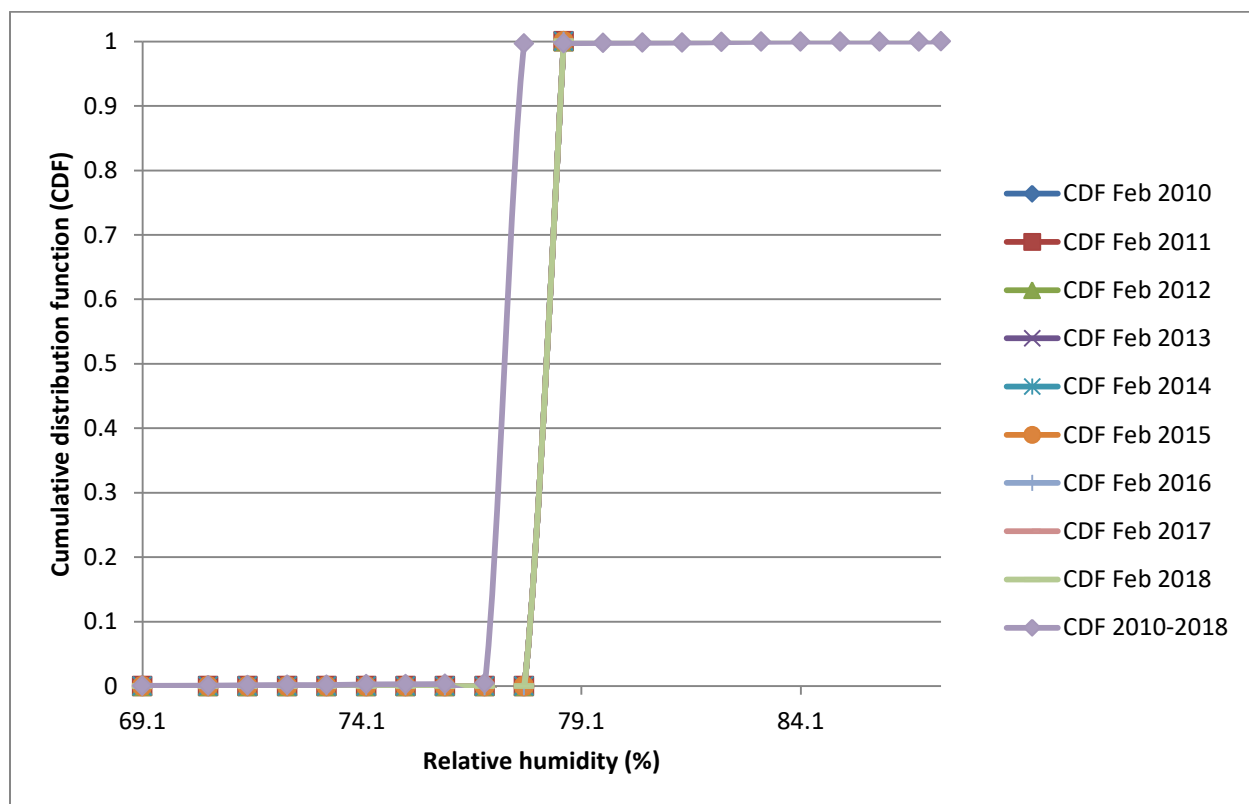



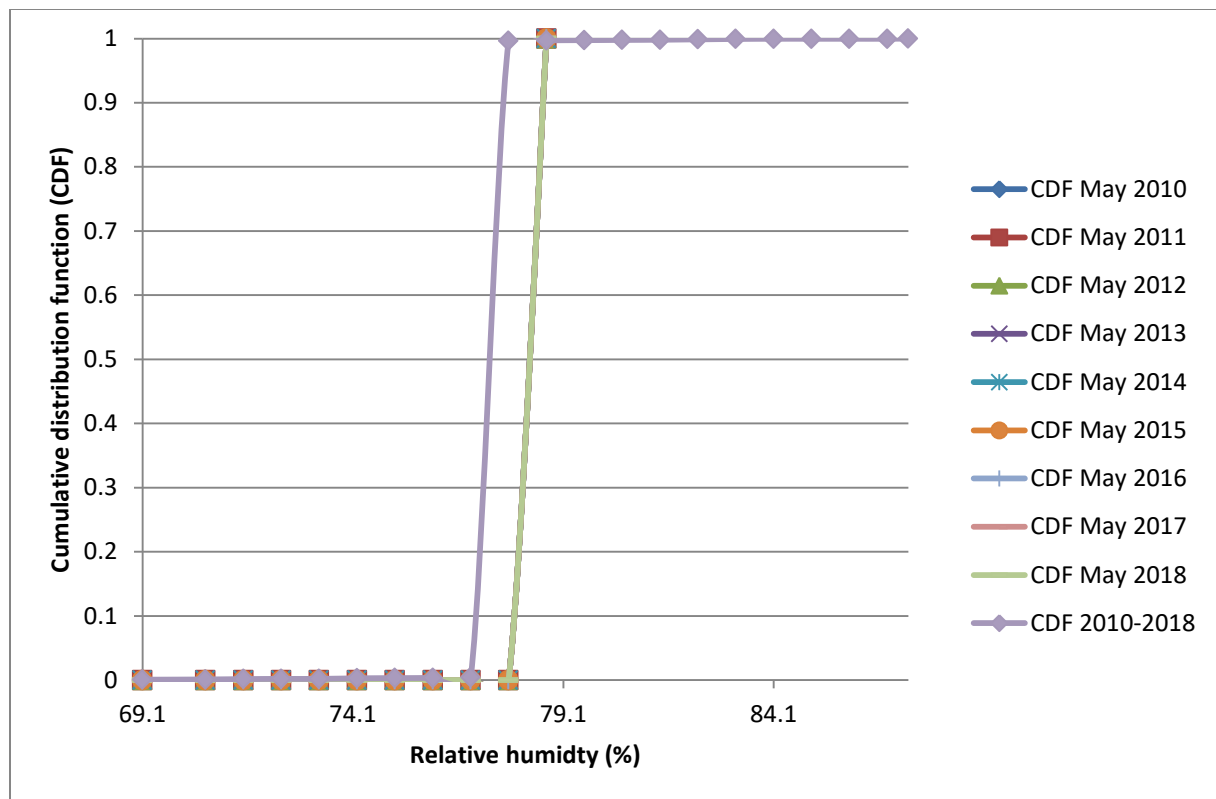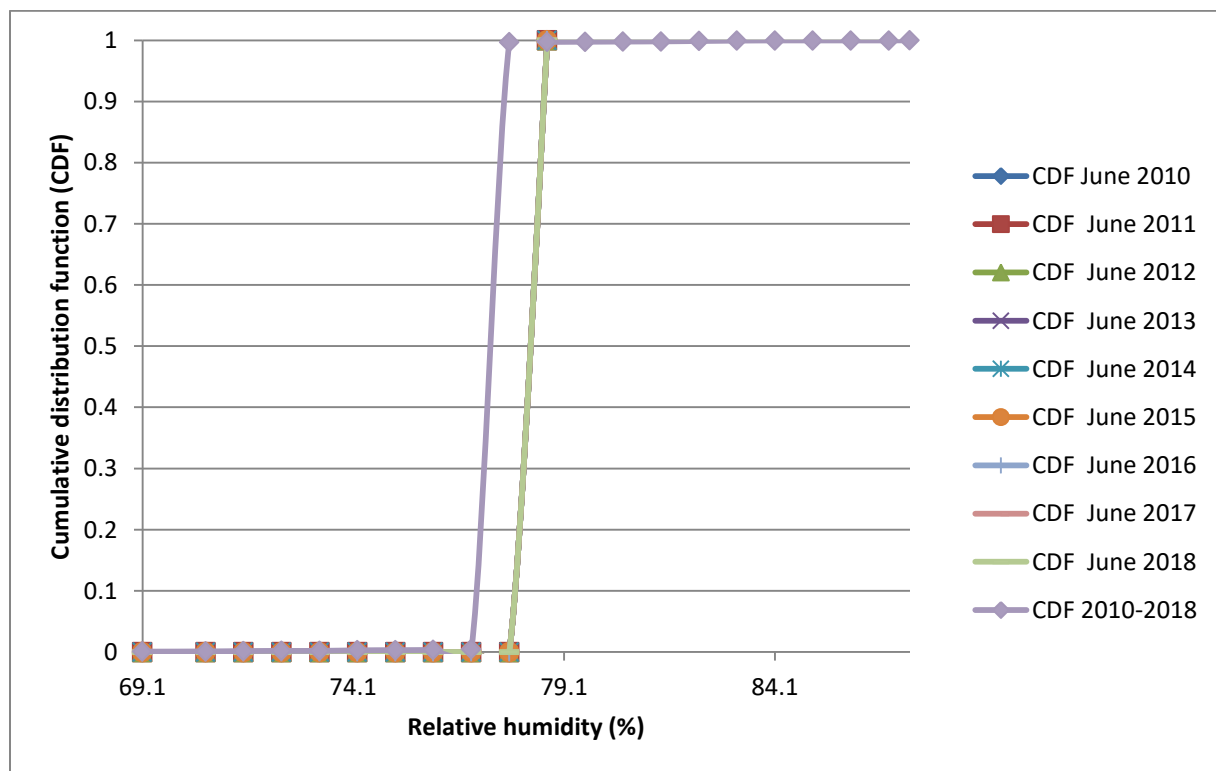



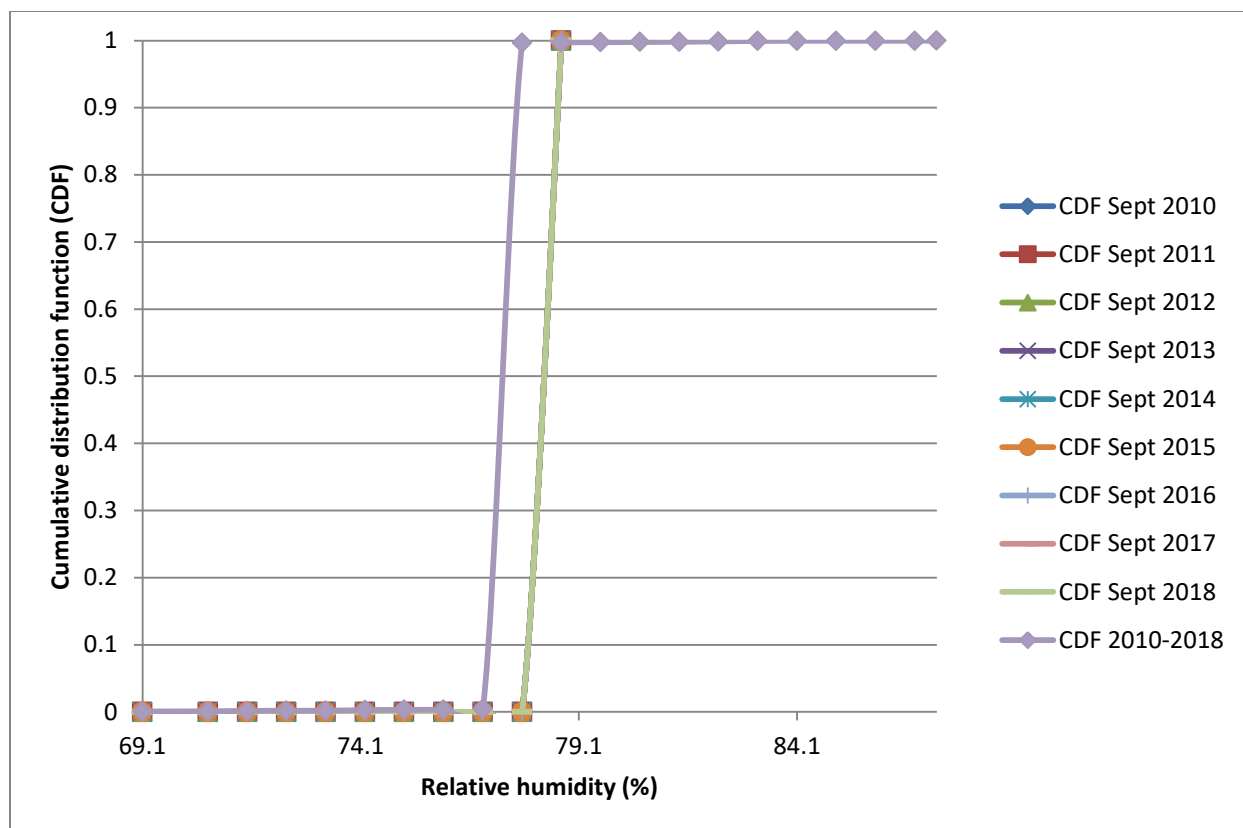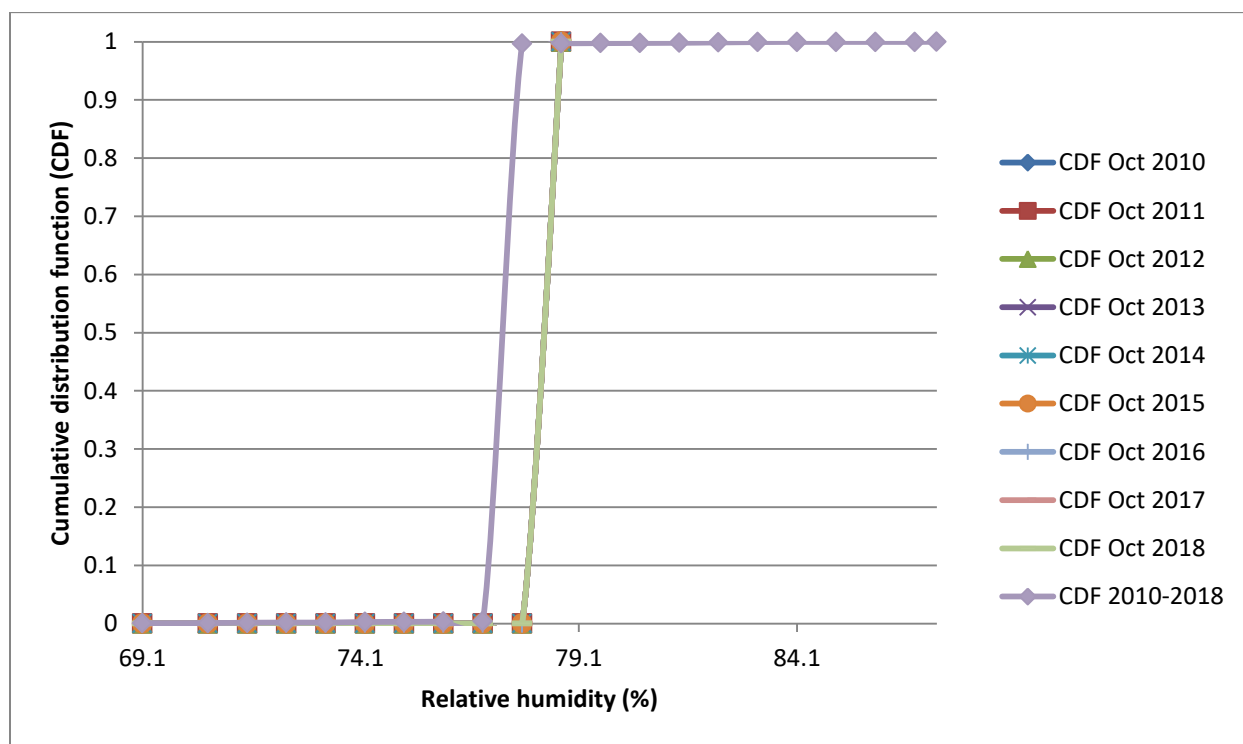

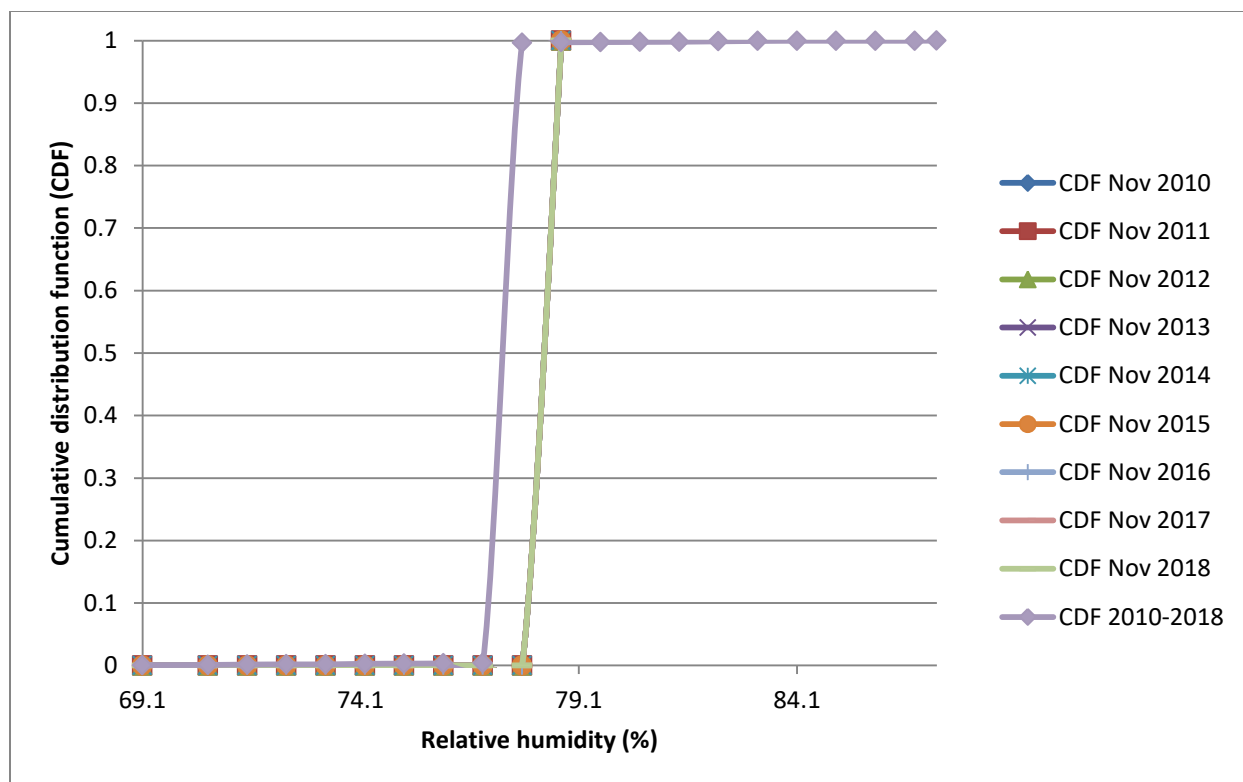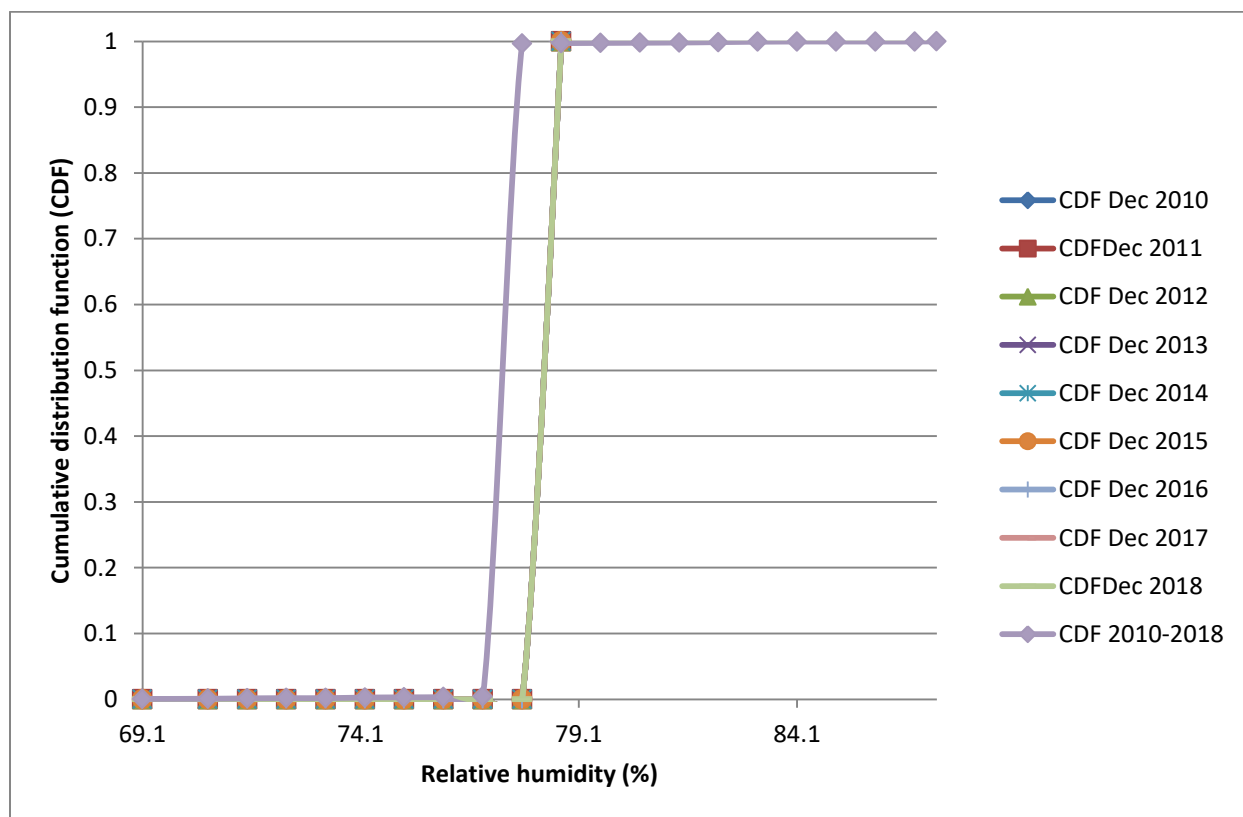

Supplement: Supplementary file 1 [file mmc1.zip › graph cdf compile.pdf]

CDF Electricity

| Electricity consumption (kWh) | CDF 2010 | CDF 2011 | CDF 2012 | CDF 2013 | CDF 2014 | CDF 2015 | CDF 2016 | CDF 2017 | CDF 2018 | CDF 2010-2018 |
|-------------------------------|----------|----------|----------|----------|----------|----------|----------|----------|----------|---------------|
| 989502                        | 0        | 0        | 0        | 0        | 0        | 0        | 0.166667 | 0        | 0        | 0.01852       |
| 1054999                       | 0        | 0        | 0        | 0        | 0        | 0        | 0.166667 | 0        | 0        | 0.01852       |
| 1089999                       | 0        | 0        | 0        | 0        | 0        | 0        | 0.166667 | 0        | 0        | 0.01852       |
| 1124999                       | 0        | 0        | 0        | 0        | 0        | 0        | 0.166667 | 0.083333 | 0        | 0.02778       |
| 1159999                       | 0        | 0        | 0        | 0        | 0        | 0.083333 | 0.166667 | 0.166667 | 0        | 0.0463        |
| 1194999                       | 0        | 0        | 0        | 0        | 0        | 0.166667 | 0.166667 | 0.166667 | 0        | 0.05556       |
| 1229999                       | 0        | 0        | 0        | 0        | 0        | 0.166667 | 0.166667 | 0.166667 | 0        | 0.05556       |
| 1264999                       | 0        | 0        | 0        | 0.083333 | 0        | 0.166667 | 0.5      | 0.25     | 0.25     | 0.13889       |
| 1299999                       | 0        | 0.083333 | 0        | 0.083333 | 0        | 0.166667 | 0.666667 | 0.416667 | 0.25     | 0.18519       |
| 1334999                       | 0        | 0.083333 | 0        | 0.166667 | 0.166667 | 0.166667 | 0.666667 | 0.583333 | 0.25     | 0.23148       |
| 1369999                       | 0        | 0.083333 | 0        | 0.166667 | 0.166667 | 0.25     | 0.833333 | 0.583333 | 0.416667 | 0.27778       |
| 1404999                       | 0.25     | 0.166667 | 0.083333 | 0.166667 | 0.166667 | 0.416667 | 0.833333 | 0.583333 | 0.416667 | 0.34259       |
| 1439999                       | 0.25     | 0.166667 | 0.083333 | 0.166667 | 0.25     | 0.5      | 1        | 0.666667 | 0.5      | 0.39815       |
| 1474999                       | 0.333333 | 0.25     | 0.166667 | 0.166667 | 0.25     | 0.666667 |          | 0.833333 | 0.5      | 0.46296       |
| 1509999                       | 0.416667 | 0.416667 | 0.166667 | 0.166667 | 0.333333 | 0.666667 |          | 0.916667 | 0.5      | 0.50926       |
| 1544999                       | 0.416667 | 0.5      | 0.333333 | 0.25     | 0.333333 | 0.833333 |          | 0.916667 | 0.666667 | 0.58333       |
| 1579999                       | 0.416667 | 0.666667 | 0.5      | 0.25     | 0.416667 | 0.833333 |          | 0.916667 | 0.75     | 0.63889       |
| 1614999                       | 0.416667 | 0.75     | 0.583333 | 0.333333 | 0.583333 | 0.916667 |          | 0.916667 | 0.75     | 0.7037        |
| 1649999                       | 0.583333 | 0.75     | 0.75     | 0.333333 | 0.75     | 1        |          | 0.916667 | 0.75     | 0.75926       |
| 1684999                       | 0.833333 | 0.916667 | 0.75     | 0.833333 | 0.916667 |          |          | 1        | 0.833333 | 0.89815       |
| 1719999                       | 0.833333 | 0.916667 | 0.833333 | 0.916667 | 1        |          |          |          | 1        | 0.92593       |
| 1754999                       | 0.833333 | 1        | 0.916667 | 0.916667 |          |          |          |          |          | 0.96296       |
| 1786162                       | 1        |          | 1        | 1        |          |          |          |          |          | 1             |

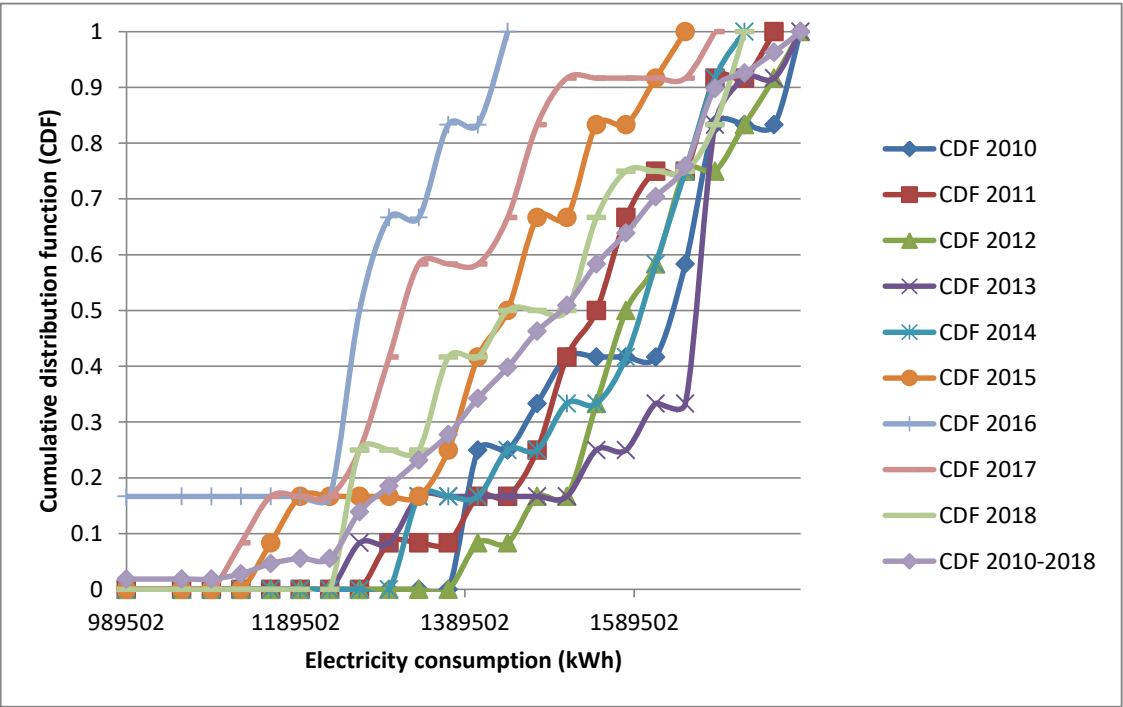

Supplement: Supplementary file 2 [file mmc2.zip › 2)CDF electricity.pdf]
