# Supplementary material for: Data Assessment on the relationship between typical weather data and electricity consumption of academic building in Melaka
Source: Data Brief. 2021 Feb 1;35:106797. doi: 10.1016/j.dib.2021.106797 (PMC7881228; doi:10.1016/j.dib.2021.106797)
Supplement: Supplementary file 2 [file mmc2.zip › 1)best and worst CDF temperature humidity rainfall CDF.pdf]

Best and Worst CDF Temperature, Humidity and Rainfall CDF

	Best Jan 2016	Worst Jan 2011	2010-2018
22.6	0	0	0.00056561
23.1	0	0	0.00056561
23.5	0	0.032258065	0.00113122
23.9	0	0.064516129	0.00367647
24.3	0	0.096774194	0.00593891
24.7	0	0.129032258	0.00961538
25.1	0	0.290322581	0.01923077
25.5	0	0.451612903	0.03054299
25.9	0	0.548387097	0.06419683
26.3	0.032258065	0.64516129	0.12471719
26.7	0.032258065	0.806451613	0.21125566
27.1	0.064516129	0.935483871	0.32352941
27.5	0.096774194	1	0.46804299
27.9	0.161290323		0.61877828
28.3	0.322580645		0.7550905
28.7	0.64516129		0.87386878
29.1	0.903225806		0.9428733
29.5	1		0.97822398
29.9			0.99236425
30.3			0.99802036
30.7			1

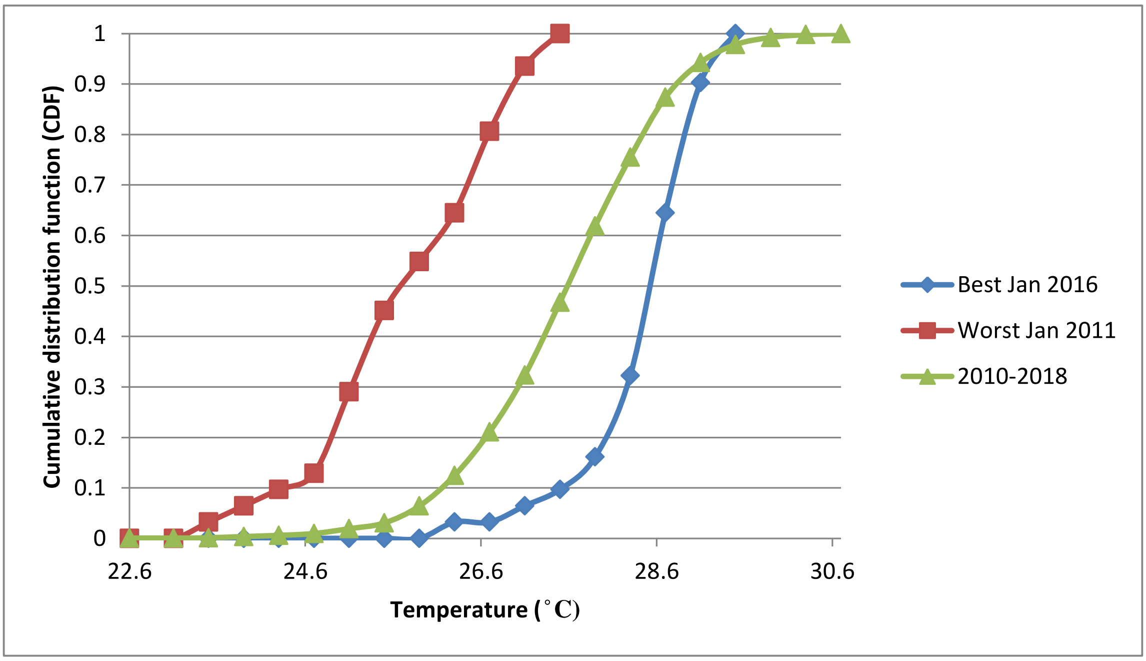

	Best Jan 2010	Worst Jan 2011	2010-2018
69.1	0.064516129	0	0.00060846
70.6	0.096774194	0	0.00091269
71.5	0.161290323	0	0.00152114
72.4	0.193548387	0	0.00182537
73.3	0.193548387	0	0.00182537
74.2	0.290322581	0	0.00273806
75.1	0.322580645	0	0.00304229
76	0.35483871	0	0.00334652
76.9	0.451612903	0	0.0042592
77.8	0.64516129	0	0.99665348
78.7	0.709677419	1	0.99726194
79.6	0.741935484		0.99756617
80.5	0.774193548		0.9978704
81.4	0.806451613		0.99817463
82.3	0.870967742		0.99878308
83.2	0.935483871		0.99939154
84.1	0.967741935		0.99969577
85	0.967741935		0.99969577
85.9	0.967741935		0.99969577
86.8	0.967741935		0.99969577
87.3	1		1

Best and Worst CDF Te

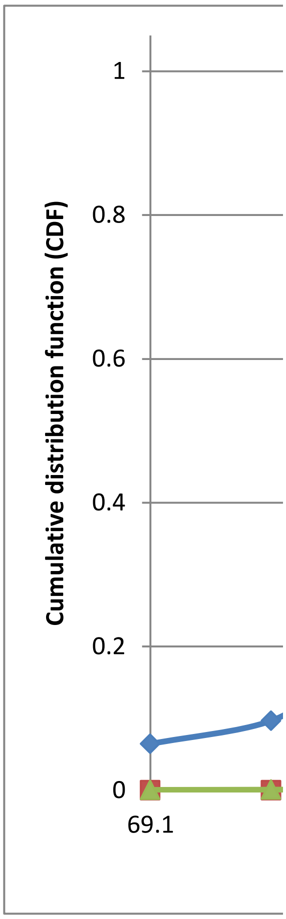

emperature, Humidity and Rainfall CDF

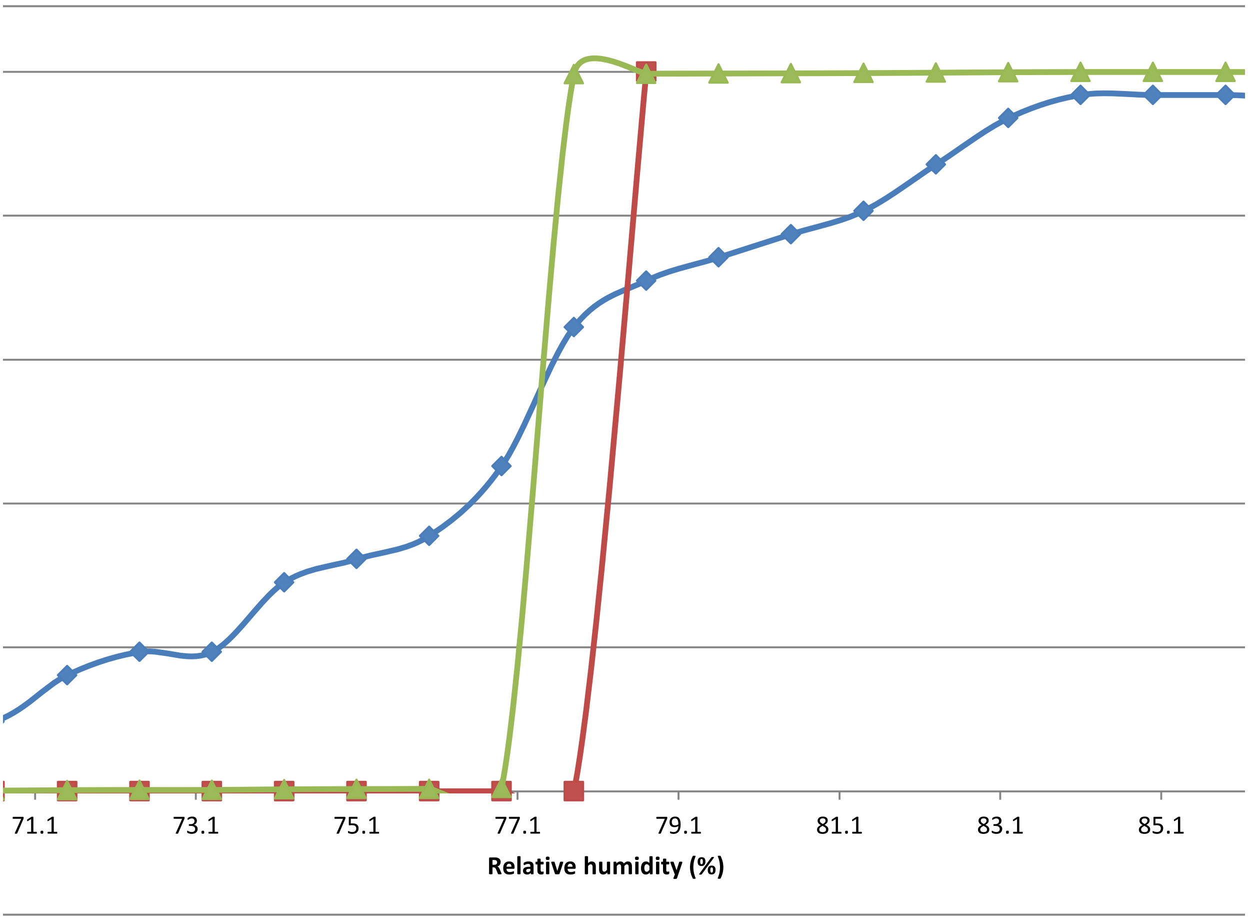

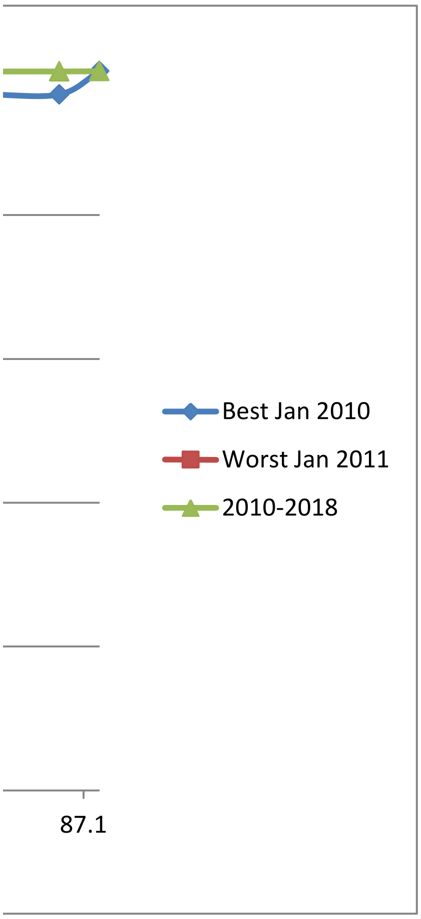
87.1
Best Jan 2010
Worst Jan 2011
2010-2018

	Best Jan 2011	Worst Jan 2014	2010-2018
4.9	0.741935484	0.903225806	0.75996349
9.90	0.774193548	0.935483871	0.83176148
14.9	0.806451613	1	0.87496197
19.9	0.806451613		0.90447216
24.9	0.870967742		0.92728932
29.9	0.903225806		0.94584728
34.9	0.903225806		0.95953757
39.9	0.903225806		0.96805598
44.9	0.967741935		0.9756617
49.9	0.967741935		0.98326742
54.9	0.967741935		0.98661393
59.9	0.967741935		0.98935199
64.9	0.967741935		0.99300274
69.9	0.967741935		0.99482811
74.9	0.967741935		0.99634925
79.9	0.967741935		0.99726194
84.9	0.967741935		0.99817463
89.9	1		0.99878308
94.9			0.99908731
99.9			0.99908731
104.9			0.99908731
109.9			0.99939154
114.9			0.99969577
119.9			0.99969577
124.9			0.99969577
129.9			0.99969577
134.9			0.99969577
139.9			0.99969577
144.9			0.99969577
149.9			0.99969577
151.8			1

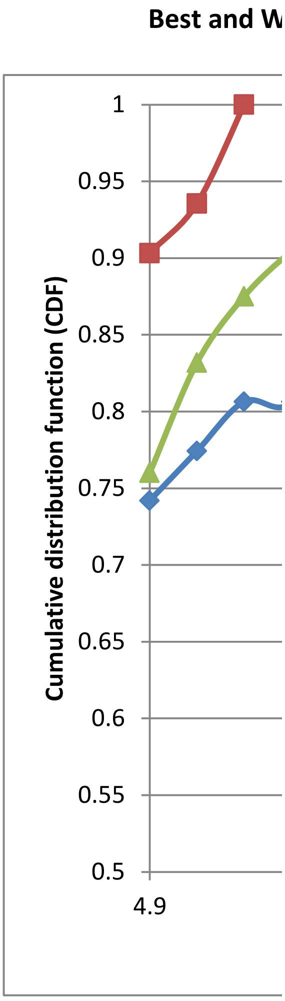

/orst CDF Temperature, Humidity and Rainfall CDF

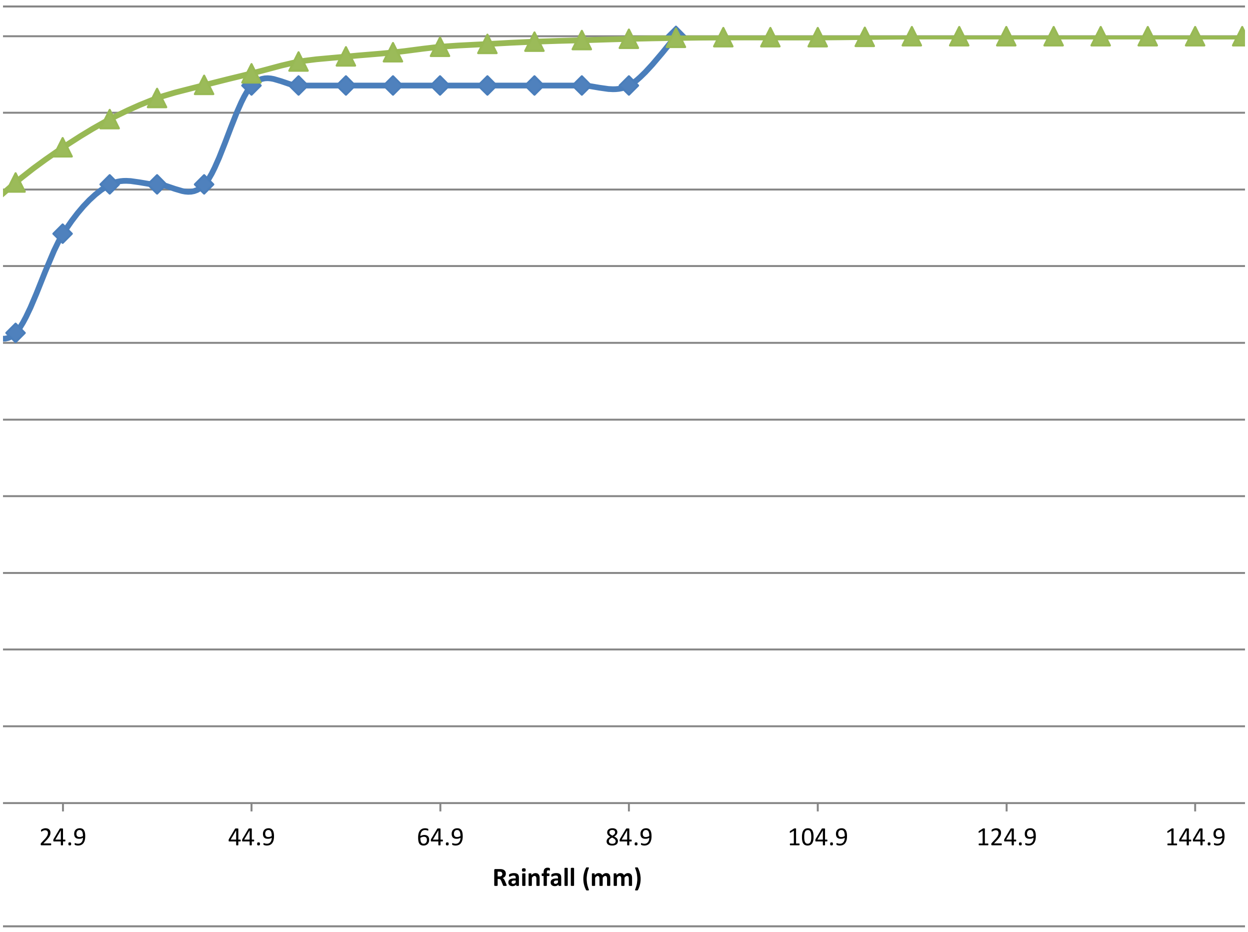

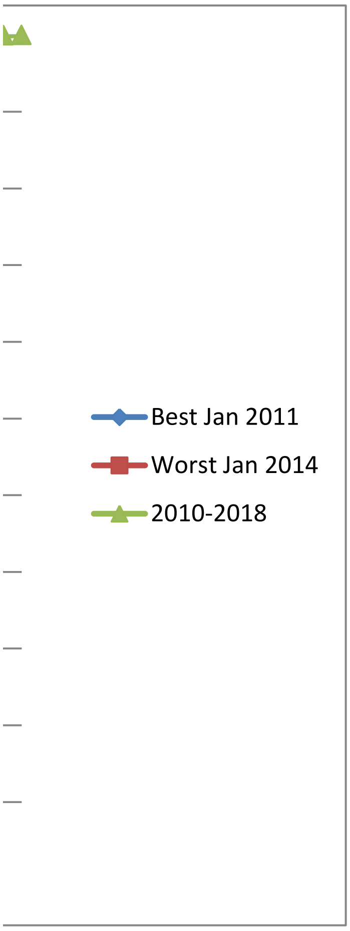
Best Jan 2011
Worst Jan 2014
2010-2018
