# Supplementary material for: Data Assessment on the relationship between typical weather data and electricity consumption of academic building in Melaka
Source: Data Brief. 2021 Feb 1;35:106797. doi: 10.1016/j.dib.2021.106797 (PMC7881228; doi:10.1016/j.dib.2021.106797)
Supplement: Supplementary file 2 [file mmc2.zip › 3)Melaka 2010-2018 test reference year TRY weather data.pdf]

Melaka 2010-2018 test Reference

Day	24 Hour Mean Temperature (˚C)
1	26
2	27.5
3	28.2
4	28.6
5	29
6	29.2
7	28.4
8	28.8
9	28.8
10	28.7
11	29
12	29.4
13	28.9
14	28.6
15	27.7
16	28.1
17	28
18	28.6
19	28
20	28.6
21	28.7
22	28.8
23	29.2
24	29
25	28.4
26	27.1
27	28.1
28	28.4
29	27.7
30	29
31	28.5
1	28.9
2	28.8
3	28
4	27.5
5	26.6
6	27.2
7	27
8	27
9	27.7
10	27.2
11	28.2
12	28.7
13	28.1
14	27.6
15	28.8

16	28.6
17	28
18	26.7
19	25.9
20	27.2
21	27.6
22	27.9
23	28.5
24	28.6
25	28.6
26	28.3
27	28.3
28	28.3
29	29
1	28.2
2	28.4
3	28.4
4	27.9
5	28
6	28
7	28.1
8	28
9	27.9
10	28.2
11	29
12	28.8
13	28.5
14	28.7
15	28.4
16	26.7
17	26.3
18	26.2
19	27.2
20	26.8
21	27.3
22	28.3
23	28.4
24	28.7
25	29.2
26	29.4
27	29.6
28	29
29	26.7
30	28.2
31	28.6
1	28
2	27.3

3	27.3
4	26.5
5	28
6	26.6
7	26.7
8	26.7
9	26.3
10	27.5
11	27.7
12	27.1
13	28.4
14	26.7
15	27.6
16	27.3
17	27.8
18	28.5
19	26.8
20	28.3
21	27.3
22	28.5
23	28.2
24	26
25	27.5
26	28.6
27	28.1
28	29.6
29	28.9
30	29.2
1	26.9
2	28.4
3	28.1
4	27.9
5	27.2
6	28.2
7	27.5
8	28.7
9	28.5
10	29.4
11	28.6
12	28.7
13	29.1
14	29.1
15	29.2
16	28.3
17	27.3
18	28.7
19	28.8

20	29.3
21	28.9
22	29.4
23	30
24	27.5
25	28.2
26	27.7
27	28.4
28	27.7
29	27.4
30	27.7
31	27.4
1	28.3
2	28.7
3	29.8
4	28.8
5	27.8
6	29
7	28.7
8	29.1
9	29.3
10	26.9
11	28.1
12	27.9
13	27
14	27.4
15	27.7
16	28.3
17	28.1
18	27.2
19	28.9
20	28
21	26.7
22	27.9
23	26.3
24	27.4
25	27.1
26	26.6
27	26.4
28	28.3
29	26.7
30	28.2
1	27.9
2	28.3
3	28.1
4	27
5	28.5

6	28.6
7	27.9
8	28.2
9	29.6
10	30.2
11	29.9
12	28.4
13	27.3
14	28
15	28.8
16	28.9
17	27.9
18	28.9
19	28.5
20	28.6
21	27.1
22	28.2
23	28.8
24	28.5
25	28.5
26	26.8
27	27.7
28	28.1
29	28.3
30	28.5
31	26.9
1	27.5
2	28.3
3	26.8
4	26.5
5	28.2
6	25.5
7	27.2
8	27.7
9	27.8
10	27.7
11	27.4
12	27.1
13	28.7
14	29.1
15	29.3
16	28.4
17	28.4
18	29.1
19	29
20	28.6
21	29.5

22	29.6
23	28.1
24	27.6
25	27.5
26	27.3
27	28.2
28	29.2
29	29.2
30	29.1
31	28.5
1	26
2	27.4
3	26.8
4	27.4
5	26.3
6	27.2
7	26.2
8	25.9
9	25.1
10	25.6
11	27
12	27.2
13	26.8
14	27
15	25.6
16	27.3
17	27.1
18	27.9
19	27.6
20	28
21	27.9
22	28.7
23	28.4
24	29.1
25	28
26	27.5
27	28.5
28	29.2
29	28.2
30	28.2
1	28.3
2	28
3	27.9
4	28.3
5	27.8
6	28.2
7	27.6

8	27.9
9	29.2
10	29.1
11	28.6
12	29.7
13	28.5
14	28.7
15	28.5
16	28.6
17	28.3
18	28.7
19	28.7
20	28.2
21	27.7
22	27.8
23	26.3
24	26.4
25	26.4
26	27.6
27	26.3
28	27.8
29	27.2
30	26.4
31	27.4
1	27.1
2	27.9
3	29.7
4	28.1
5	29.1
6	27.4
7	26.2
8	26.4
9	27.7
10	28.4
11	26.1
12	26.2
13	26.8
14	25.9
15	26
16	26.6
17	25.3
18	27.4
19	28.2
20	27.4
21	28
22	27.2
23	27.6

24	28.3
25	26.1
26	26
27	27.7
28	26.7
29	26
30	25.9
1	25.7
2	25.7
3	26.9
4	28.4
5	28.6
6	29.6
7	28.8
8	28.4
9	27.1
10	26.6
11	24.9
12	27.2
13	27.6
14	27.9
15	27.6
16	28.8
17	27.9
18	26.6
19	27.6
20	27.7
21	28.4
22	27
23	27.4
24	27.4
25	26.8
26	26
27	26.5
28	26.5
29	27.4
30	27.4
31	26

Year TRY Weather Data

Day	24 Hour Mean Relative Humidity (%)	**Melaka 2010-2018 tes**
1	77.5	
2	87.3	
3	80.8	
4	82.1	
5	78.6	
6	78.7	
7	83.1	
8	83.3	
9	75.7	
10	73.7	
11	76.4	
12	73.6	
13	74.1	
14	74.7	
15	69.5	
16	69.1	
17	70.8	
18	70.1	
19	81.6	
20	80.1	
21	82.4	
22	79.3	
23	76.1	
24	72.4	
25	71.1	
26	76.5	
27	77.3	
28	77.3	
29	77.3	
30	77.3	
31	77.3	
1	77.3	
2	77.3	
3	77.3	
4	77.3	
5	77.3	
6	77.3	
7	77.3	
8	77.3	
9	77.3	
10	77.3	
11	77.3	
12	77.3	
13	77.3	
14	77.3	
15	77.3	
16	77.3	
17	77.3	
18	77.3	

19	77.3
20	77.3
21	77.3
22	77.3
23	77.3
24	77.3
25	77.3
26	77.3
27	77.3
28	77.3
1	77.3
2	77.3
3	77.3
4	77.3
5	77.3
6	77.3
7	77.3
8	77.3
9	77.3
10	77.3
11	77.3
12	77.3
13	77.3
14	77.3
15	77.3
16	77.3
17	77.3
18	77.3
19	77.3
20	77.3
21	77.3
22	77.3
23	77.3
24	77.3
25	77.3
26	77.3
27	77.3
28	77.3
29	77.3
30	77.3
31	77.3
1	77.3
2	77.3
3	77.3
4	77.3
5	77.3
6	77.3
7	77.3
8	77.3
9	77.3

10	77.3
11	77.3
12	77.3
13	77.3
14	77.3
15	77.3
16	77.3
17	77.3
18	77.3
19	77.3
20	77.3
21	77.3
22	77.3
23	77.3
24	77.3
25	77.3
26	77.3
27	77.3
28	77.3
29	77.3
30	77.3
1	77.3
2	77.3
3	77.3
4	77.3
5	77.3
6	77.3
7	77.3
8	77.3
9	77.3
10	77.3
11	77.3
12	77.3
13	77.3
14	77.3
15	77.3
16	77.3
17	77.3
18	77.3
19	77.3
20	77.3
21	77.3
22	77.3
23	77.3
24	77.3
25	77.3
26	77.3
27	77.3
28	77.3
29	77.3

30	77.3
31	77.3
1	77.3
2	77.3
3	77.3
4	77.3
5	77.3
6	77.3
7	77.3
8	77.3
9	77.3
10	77.3
11	77.3
12	77.3
13	77.3
14	77.3
15	77.3
16	77.3
17	77.3
18	77.3
19	77.3
20	77.3
21	77.3
22	77.3
23	77.3
24	77.3
25	77.3
26	77.3
27	77.3
28	77.3
29	77.3
30	77.3
1	77.3
2	77.3
3	77.3
4	77.3
5	77.3
6	77.3
7	77.3
8	77.3
9	77.3
10	77.3
11	77.3
12	77.3
13	77.3
14	77.3
15	77.3
16	77.3
17	77.3
18	77.3

19	77.3
20	77.3
21	77.3
22	77.3
23	77.3
24	77.3
25	77.3
26	77.3
27	77.3
28	77.3
29	77.3
30	77.3
31	77.3
1	77.3
2	77.3
3	77.3
4	77.3
5	77.3
6	77.3
7	77.3
8	77.3
9	77.3
10	77.3
11	77.3
12	77.3
13	77.3
14	77.3
15	77.3
16	77.3
17	77.3
18	77.3
19	77.3
20	77.3
21	77.3
22	77.3
23	77.3
24	77.3
25	77.3
26	77.3
27	77.3
28	77.3
29	77.3
30	77.3
31	77.3
1	77.3
2	77.3
3	77.3
4	77.3
5	77.3
6	77.3

7	77.3
8	77.3
9	77.3
10	77.3
11	77.3
12	77.3
13	77.3
14	77.3
15	77.3
16	77.3
17	77.3
18	77.3
19	77.3
20	77.3
21	77.3
22	77.3
23	77.3
24	77.3
25	77.3
26	77.3
27	77.3
28	77.3
29	77.3
30	77.3
1	77.3
2	77.3
3	77.3
4	77.3
5	77.3
6	77.3
7	77.3
8	77.3
9	77.3
10	77.3
11	77.3
12	77.3
13	77.3
14	77.3
15	77.3
16	77.3
17	77.3
18	77.3
19	77.3
20	77.3
21	77.3
22	77.3
23	77.3
24	77.3
25	77.3
26	77.3

27	77.3
28	77.3
29	77.3
30	77.3
31	77.3
1	77.3
2	77.3
3	77.3
4	77.3
5	77.3
6	77.3
7	77.3
8	77.3
9	77.3
10	77.3
11	77.3
12	77.3
13	77.3
14	77.3
15	77.3
16	77.3
17	77.3
18	77.3
19	77.3
20	77.3
21	77.3
22	77.3
23	77.3
24	77.3
25	77.3
26	77.3
27	77.3
28	77.3
29	77.3
30	77.3
1	77.3
2	77.3
3	77.3
4	77.3
5	77.3
6	77.3
7	77.3
8	77.3
9	77.3
10	77.3
11	77.3
12	77.3
13	77.3
14	77.3
15	77.3

16	77.3
17	77.3
18	77.3
19	77.3
20	77.3
21	77.3
22	77.3
23	77.3
24	77.3
25	77.3
26	77.3
27	77.3
28	77.3
29	77.3
30	77.3
31	77.3

t Reference Year TRY Weather Data

Melaka 2010-2018 test Reference Year TRY Weather Data

Day	Rainfall (08-08 MST) (mm)
1	0
2	0
3	0
4	0
5	22.6
6	0
7	0.09
8	0
9	0.8
10	42.6
11	40.2
12	25.2
13	3.8
14	0.09
15	0.4
16	0.2
17	1.6
18	0
19	0
20	0
21	0
22	0
23	0
24	0
25	7.2
26	13.4
27	0.09
28	0
29	22.8
30	86.2
31	3.6
1	0
2	0
3	0
4	0
5	1.6
6	0.4
7	88
8	0
9	2.6
10	8
11	0
12	0
13	0
14	0
15	0
16	0

17	0.4
18	0.6
19	0
20	0
21	0.09
22	0.09
23	0.6
24	0.09
25	5.2
26	0
27	0
28	0
1	5
2	9.2
3	7
4	0
5	14.2
6	6.8
7	0
8	0
9	0
10	0
11	0
12	0
13	0
14	0.09
15	0
16	0.2
17	0
18	15.2
19	2.2
20	2
21	0.2
22	0
23	0
24	65
25	0
26	44.8
27	0
28	6.4
29	6.2
30	11.4
31	0
1	0.4
2	0
3	0
4	0
5	0
6	0
7	0

8	0
9	0
10	0
11	0.2
12	0.4
13	21
14	2
15	1.6
16	15.2
17	0.6
18	0
19	26
20	77.6
21	21.2
22	24.8
23	2.8
24	2.8
25	1.4
26	0
27	6.6
28	0
29	0
30	3.6
1	0
2	1.2
3	0.2
4	26.6
5	5
6	1
7	5.2
8	0.09
9	1.4
10	2
11	0.4
12	0.4
13	0.8
14	0.4
15	20
16	0
17	0
18	0
19	0.09
20	10
21	1.2
22	0.09
23	14.6
24	5.2
25	41.8
26	0
27	65.4

28	4.2
29	0
30	4
31	0
1	0.4
2	11.4
3	0
4	0
5	0
6	0
7	1.2
8	0.09
9	0
10	0
11	69
12	52
13	0
14	3.4
15	2.8
16	114.8
17	1.8
18	0
19	11.8
20	0
21	3.6
22	0.09
23	1.6
24	0
25	65
26	1.4
27	0
28	0
29	0
30	0
1	0
2	0.4
3	0
4	1.4
5	2.6
6	0
7	60
8	3
9	0.8
10	10
11	40
12	0
13	26
14	0
15	0
16	0

17	0
18	0
19	0
20	0
21	0
22	0
23	0
24	0
25	17.6
26	0
27	7.6
28	0
29	0
30	94
31	22.6
1	41.4
2	12.2
3	0
4	0
5	14.6
6	1.8
7	0
8	9
9	0
10	151.8
11	0.8
12	64.4
13	15.8
14	46.4
15	7.6
16	17.2
17	0.09
18	0
19	0
20	0
21	0
22	0
23	0
24	1
25	0
26	19.2
27	0.09
28	45.2
29	0
30	0
31	0.2
1	0.2
2	0
3	3.2
4	0.4

5	3
6	7.4
7	0
8	21.8
9	8.2
10	1.4
11	0.8
12	0
13	8
14	11.6
15	77.2
16	0.8
17	0.4
18	10
19	1
20	1.4
21	0
22	11.2
23	2.8
24	0
25	10
26	0.2
27	0.4
28	0
29	0.8
30	3.2
1	0.2
2	1.2
3	0.2
4	0.09
5	0
6	0
7	0
8	5.2
9	0
10	84
11	0.4
12	0
13	0
14	0
15	0
16	1
17	20.6
18	0.09
19	22.6
20	0.4
21	0.09
22	0.09
23	0
24	0.8

25	11.8
26	7.4
27	32.8
28	0.6
29	6.8
30	3.8
31	11.4
1	0.2
2	17.2
3	9.4
4	11
5	3.8
6	6.4
7	71.2
8	0.2
9	0
10	0
11	0.8
12	2.2
13	30.8
14	14
15	1.6
16	3
17	21.6
18	0.2
19	0
20	0.2
21	0.2
22	0
23	49.2
24	0
25	0.09
26	0.6
27	0.6
28	1
29	0
30	0.2
1	84
2	0.2
3	0
4	0
5	4
6	0
7	0
8	0.09
9	0.09
10	15.4
11	6.6
12	50.6
13	34.2

14	0
15	0
16	0.2
17	0
18	0
19	0.2
20	19
21	4.8
22	2.6
23	0.09
24	12
25	0
26	0
27	0
28	2.2
29	0.2
30	0
31	39.4
