# Supplementary material for: Data Assessment on the relationship between typical weather data and electricity consumption of academic building in Melaka
Source: Data Brief. 2021 Feb 1;35:106797. doi: 10.1016/j.dib.2021.106797 (PMC7881228; doi:10.1016/j.dib.2021.106797)
Supplement: Supplementary file 2 [file mmc2.zip › 4)TRY ultimate best weather best electricity.pdf]

TRY Ultimately Best Weather Best Electricity

Month	Temperatu	Relative hu	Rainfall	Electricity consumption
1	28.419	76.939	8.739	1611564
2	27.89	77.3	3.845	1463966
3	28.1	77.3	6.319	1768383
4	27.633	77.3	6.94	1766189
5	28.329	77.3	6.815	1477393
6	27.887	77.3	11.346	1382741
7	28.287	77.3	9.226	1656143
8	28.132	77.3	14.477	1671783
9	27.303	77.3	6.18	1404413
10	27.939	77.3	6.825	1653637
11	27.113	77.3	8.19	1625158
12	27.303	77.3	8.899	1393575
