# Supplementary material for: Data Assessment on the relationship between typical weather data and electricity consumption of academic building in Melaka
Source: Data Brief. 2021 Feb 1;35:106797. doi: 10.1016/j.dib.2021.106797 (PMC7881228; doi:10.1016/j.dib.2021.106797)
Supplement: Supplementary file 2 [file mmc2.zip › 5)TRY weather data vs electricity.pdf]

TRY Weather Data VS Electricity

	Month	Temperatu	Relative hu	Rainfall	Electricity Consumption
2010	January	28.419	76.939	8.739	1611564
	February	27.89	77.3	3.845	1463966
	March	28.1	77.3	6.319	1768383
	April	27.633	77.3	6.94	1766189
	May	28.329	77.3	6.815	1477393
	June	27.887	77.3	11.346	1382741
	July	28.287	77.3	9.226	1656143
	August	28.132	77.3	14.477	1671783
	September	27.303	77.3	6.18	1404413
	October	27.939	77.3	6.825	1653637
	November	27.113	77.3	8.19	1625158
	December	27.303	77.3	8.899	1393575
2011	January	28.419	76.939	8.739	1528600
	February	27.89	77.3	3.845	1395482
	March	28.1	77.3	6.319	1734956
	April	27.633	77.3	6.94	1651776
	May	28.329	77.3	6.815	1581785
	June	27.887	77.3	11.346	1456533
	July	28.287	77.3	9.226	1499569
	August	28.132	77.3	14.477	1297746
	September	27.303	77.3	6.18	1501514
	October	27.939	77.3	6.825	1673928
	November	27.113	77.3	8.19	1558433
	December	27.303	77.3	8.899	1578540
2012	January	28.419	76.939	8.739	1539977
	February	27.89	77.3	3.845	1444873
	March	28.1	77.3	6.319	1558944
	April	27.633	77.3	6.94	1616455
	May	28.329	77.3	6.815	1739242
	June	27.887	77.3	11.346	1697811
	July	28.287	77.3	9.226	1533023
	August	28.132	77.3	14.477	1372868
	September	27.303	77.3	6.18	1619114
	October	27.939	77.3	6.825	1781660
	November	27.113	77.3	8.19	1576230
	December	27.303	77.3	8.899	1611308
2013	January	28.419	76.939	8.739	1717005
	February	27.89	77.3	3.845	1238072
	March	28.1	77.3	6.319	1683266
	April	27.633	77.3	6.94	1666782
	May	28.329	77.3	6.815	1668865
	June	27.887	77.3	11.346	1666473
	July	28.287	77.3	9.226	1517540
	August	28.132	77.3	14.477	1314058
	September	27.303	77.3	6.18	1592058
	October	27.939	77.3	6.825	1786162
	November	27.113	77.3	8.19	1663784

	December	27.303	77.3	8.899	1651823
2014	January	28.419	76.939	8.739	1493724
	February	27.89	77.3	3.845	1409565
	March	28.1	77.3	6.319	1594135
	April	27.633	77.3	6.94	1662174
	May	28.329	77.3	6.815	1671457
	June	27.887	77.3	11.346	1600936
	July	28.287	77.3	9.226	1306621
	August	28.132	77.3	14.477	1327291
	September	27.303	77.3	6.18	1640025
	October	27.939	77.3	6.825	1617286
	November	27.113	77.3	8.19	1561161
	December	27.303	77.3	8.899	1703293
2015	January	28.419	76.939	8.739	1434815
	February	27.89	77.3	3.845	1155469
	March	28.1	77.3	6.319	1512537
	April	27.633	77.3	6.94	1614452
	May	28.329	77.3	6.815	1511777
	June	27.887	77.3	11.346	1456892
	July	28.287	77.3	9.226	1194836
	August	28.132	77.3	14.477	1358746
	September	27.303	77.3	6.18	1457974
	October	27.939	77.3	6.825	1621564
	November	27.113	77.3	8.19	1401026
	December	27.303	77.3	8.899	1391534
2016	January	28.419	76.939	8.739	1254646
	February	27.89	77.3	3.845	1015821
	March	28.1	77.3	6.319	1435090
	April	27.633	77.3	6.94	1369935
	May	28.329	77.3	6.815	1363603
	June	27.887	77.3	11.346	1234053
	July	28.287	77.3	9.226	989502
	August	28.132	77.3	14.477	1256682
	September	27.303	77.3	6.18	1284336
	October	27.939	77.3	6.825	1250492
	November	27.113	77.3	8.19	1407571
	December	27.303	77.3	8.899	1291312
2017	January	28.419	76.939	8.739	1127583
	February	27.89	77.3	3.845	1119332
	March	28.1	77.3	6.319	1452876
	April	27.633	77.3	6.94	1322393
	May	28.329	77.3	6.815	1422925
	June	27.887	77.3	11.346	1232941
	July	28.287	77.3	9.226	1308429
	August	28.132	77.3	14.477	1274299
	September	27.303	77.3	6.18	1284743
	October	27.939	77.3	6.825	1501696
	November	27.113	77.3	8.19	1656220
	December	27.303	77.3	8.899	1451225
2018	January	28.419	76.939	8.739	1412975

February	27.89	77.3	3.845	1358098
March	28.1	77.3	6.319	1679658
April	27.633	77.3	6.94	1738358
May	28.329	77.3	6.815	1552301
June	27.887	77.3	11.346	1234851
July	28.287	77.3	9.226	1261872
August	28.132	77.3	14.477	1237152
September	27.303	77.3	6.18	1360173
October	27.939	77.3	6.825	1727495
November	27.113	77.3	8.19	1514314
December	27.303	77.3	8.899	1513593
